# Nurse-Led Strategy to Improve Blood Pressure and Cholesterol Level Among People With HIV: A Randomized Clinical Trial

**DOI:** 10.1001/jamanetworkopen.2023.56445

**Published:** 2024-03-05

**Authors:** Christopher T. Longenecker, Kelley A. Jones, Corrilynn O. Hileman, Nwora Lance Okeke, Barbara M. Gripshover, Angela Aifah, Gerald S. Bloomfield, Charles Muiruri, Valerie A. Smith, Rajesh Vedanthan, Allison R. Webel, Hayden B. Bosworth

**Affiliations:** 1 University of Washington School of Medicine, Seattle; 2 Duke University School of Medicine, Durham, North Carolina; 3 MetroHealth Medical Center, Cleveland, Ohio; 4 Case Western Reserve University School of Medicine, Cleveland, Ohio; 5 University Hospitals Cleveland Medical Center, Cleveland, Ohio; 6 New York University Grossman School of Medicine, New York; 7 Durham Veterans Affairs Medical Center, Durham, North Carolina; 8 University of Washington School of Nursing, Seattle

## Abstract

**Importance:**

Despite higher atherosclerotic cardiovascular disease (ASCVD) risk, people with HIV (PWH) experience unique barriers to ASCVD prevention, such as changing models of HIV primary care.

**Objective:**

To test whether a multicomponent nurse-led strategy would improve systolic blood pressure (SBP) and non–high-density lipoprotein (HDL) cholesterol level in a diverse population of PWH receiving antiretroviral therapy (ART).

**Design, Setting, and Participants:**

This randomized clinical trial enrolled PWH at 3 academic HIV clinics in the US from September 2019 to January 2022 and conducted follow-up for 12 months until January 2023. Included patients were 18 years or older and had a confirmed HIV diagnosis, an HIV-1 viral load less than 200 copies/mL, and both hypertension and hypercholesterolemia. Participants were stratified by trial site and randomized 1:1 to either the multicomponent EXTRA-CVD (A Nurse-Led Intervention to Extend the HIV Treatment Cascade for Cardiovascular Disease Prevention) intervention group or the control group. Primary analyses were conducted according to the intention-to-treat principle.

**Intervention:**

The EXTRA-CVD group received home BP monitoring guidance and BP and cholesterol management from a dedicated prevention nurse at 4 in-person visits (baseline and 4, 8, and 12 months) and frequent telephone check-ins up to every 2 weeks as needed. The control group received general prevention education sessions from the prevention nurse at each of the 4 in-person visits.

**Main Outcomes and Measures:**

Study-measured SBP was the primary outcome, and non–HDL cholesterol level was the secondary outcome. Measurements were taken over 12 months and assessed by linear mixed models. Prespecified moderators tested were sex at birth, baseline ASCVD risk, and trial site.

**Results:**

A total of 297 PWH were randomized to the EXTRA-CVD arm (n = 149) or control arm (n = 148). Participants had a median (IQR) age of 59.0 (53.0-65.0) years and included 234 males (78.8%). Baseline mean (SD) SBP was 135.0 (18.8) mm Hg and non–HDL cholesterol level was 139.9 (44.6) mg/dL. At 12 months, participants in the EXTRA-CVD arm had a clinically significant 4.2-mm Hg (95% CI, 0.3-8.2 mm Hg; *P* = .04) lower SBP and 16.9-mg/dL (95% CI, 8.6-25.2 mg/dL; *P* < .001) lower non–HDL cholesterol level compared with participants in the control arm. There was a clinically meaningful but not statistically significant difference in SBP effect in females compared with males (11.8–mm Hg greater difference at 4 months, 9.6 mm Hg at 8 months, and 5.9 mm Hg at 12 months; overall joint test *P* = .06).

**Conclusions and Relevance:**

Results of this trial indicate that the EXTRA-CVD strategy effectively reduced BP and cholesterol level over 12 months and should inform future implementation of multifaceted ASCVD prevention programs for PWH.

**Trial Registration:**

ClinicalTrials.gov Identifier: NCT03643705

## Key Points

**Question:**

Does a multicomponent strategy of nurse-led care coordination, home blood pressure monitoring, evidence-based treatment algorithms, and electronic health record tools improve systolic blood pressure and non–high-density lipoprotein (HDL) cholesterol level over 12 months in people with HIV?

**Findings:**

In this randomized clinical trial involving 297 participants with HIV receiving care at 3 academic HIV clinics in the US, randomization to the intervention arm resulted in a significantly lower systolic blood pressure and non–HDL cholesterol level at 12 months compared with randomization to the control group, which received general prevention education.

**Meaning:**

Findings of this trial suggest that nurse-led cardiovascular risk factor management in academic HIV clinics may lead to fewer cardiovascular events and should inform implementation of prevention programs for people with HIV.

## Teaser

This randomized clinical trial assesses the effectiveness of a cardiovascular risk factor management intervention supported by a dedicated nurse, evidence-based treatment algorithms, and electronic health record tools in an academic HIV clinic setting.

## Introduction

Prevention of atherosclerotic cardiovascular disease (ASCVD) is an emerging priority for people with HIV (PWH) in the era of effective antiretroviral therapy (ART). Once HIV viral replication is effectively suppressed in PWH receiving ART, an opportunity emerges to shift focus toward overall general health promotion and disease prevention.^1^ For example, the HIV treatment cascade should be extended for cardiovascular disease prevention by appropriately diagnosing, managing, and controlling ASCVD risk factors. In the US and throughout the world, there are unique barriers to ASCVD prevention care, such as changing models of HIV primary care and mistrust of non–HIV care practitioners.^2,3^

Implementation of strategies to overcome these barriers is needed to increase risk factor control and ultimately reduce clinical ASCVD events. On a population level, high blood pressure (BP) and high cholesterol level are 2 leading factors in myocardial infarction among PWH in North America,^4^ yet appropriate management of these conditions is suboptimal and lags behind management in the general population.^5,6,7^ Using a human-centered design approach,^8^ our team engaged PWH and their care practitioners to develop and adapt EXTRA-CVD (A Nurse-Led Intervention to Extend the HIV Treatment Cascade for Cardiovascular Disease Prevention). The premise of this strategy is that task sharing with nurses has improved and scaled-up HIV and hypertension care in Africa^9,10,11^ and can be adapted to the US context.

Based on prior experience,^9,12,13^ the multicomponent EXTRA-CVD strategy was initially conceived with 4 main components^14^: (1) nurse-led care coordination, (2) home BP monitoring, (3) evidence-based treatment algorithms, and (4) electronic health record (EHR) tools. Additionally, PWH and diverse health care practitioners were engaged in an iterative design process to tailor the strategy through prototyping, pilot testing, and feasibility or acceptability testing.^8^ In the present clinical trial, we tested whether EXTRA-CVD would improve systolic BP (SBP) and non–high-density lipoprotein (HDL) cholesterol level in a diverse population of PWH with suppressed HIV-1 viral load receiving ART.

## Methods

EXTRA-CVD is a hybrid type 1 implementation–effectiveness trial, which has been described in detail elsewhere.^14^ Its primary effectiveness outcomes are reported here. We conducted the present randomized clinical trial in 3 academic medical centers that provide HIV care across 2 US states. We planned to enroll 300 PWH with hypertension and hyperlipidemia (100 per site) and randomize them 1:1 to the intervention or control arm stratified by site. The University Hospitals Cleveland Medical Center Institutional Review Board, with reliant review at MetroHealth Medical Center and Duke Health, approved the trial. All participants signed written informed consent. An independent Data and Safety Monitoring Board reviewed the study progress, outcomes, and safety every 6 months. The trial protocol and statistical analysis plan are provided in Supplement 1. We followed the Consolidated Standards of Reporting Trials (CONSORT) reporting guideline.

Inclusion criteria were (1) age 18 years or older, (2) confirmed HIV-positive diagnosis, (3) undetectable HIV viral load (<200 copies/mL) within past year, (4) hypertension (SBP>130 mm Hg on ≥2 visits in past 12 months or prescribed an antihypertensive medication^15^), and (5) hyperlipidemia (non–HDL cholesterol level >130 mg/dL or prescribed a cholesterol-lowering medication; to convert to millimoles per liter, multiply by 0.0259). Patients were excluded if they were receiving antihypertensive medication for a nonhypertension reason or cholesterol-lowering medication with evidence of premedication non–HDL cholesterol level less than 100 mg/dL.

### Recruitment and Retention

Enrollment began in September 2019 and continued through January 2022 (eFigure 1 in Supplement 2), with final follow-up completed in January 2023. We used the EHR at the 3 medical centers to identify potential participants on the basis of age, HIV diagnosis, viral load, and hypertension and hyperlipidemia status. Potential participants were initially contacted via a mailed letter or EHR message or approached at their in-person clinic visit. Those who did not opt out were contacted for participant screening and scheduled for an in-person baseline visit conducted by a trained research assistant and a prevention nurse.

Participants completed 4 study visits: baseline and follow-up visits at 4, 8, and 12 months. At each visit, participants completed self-reported outcome surveys, a fasting blood draw, and outcome BP measurement. During the early phase of the COVID-19 pandemic (March to August 2020), new enrollments were paused. For participants already enrolled during this period, parts of the follow-up visits were conducted remotely; however, BP and cholesterol outcome measures were obtained in person.

### EXTRA-CVD Intervention and Education Control Arms

During the trial, the 4 key components of EXTRA-CVD were delivered by a prevention nurse (hired at each site specifically for this trial) who communicated frequently with participants and interacted directly with participants’ health care practitioners. The components included specific type and frequency of nurse-led communication, home BP monitoring guidance, and evidence-based algorithms for BP and cholesterol management. The EHR tools, another component, included a dashboard function to help the prevention nurse with managing patients in the panel and pending orders for physicians. At a minimum, the prevention nurse met with participants assigned to the EXTRA-CVD intervention arm 2 weeks after baseline and at the midpoint between in-person visits every 4 months. Additional telephone check-ins up to every 2-weeks occurred as necessary to coordinate clinical care and titrate medications. Fidelity to intervention delivery was monitored quarterly using checklists. Quarterly boot camps for prevention nurses were held via online conferences (Zoom Video Communications) to discuss lessons learned across sites and to learn about topics in BP and cholesterol management. All prevention nurses were trained in motivational interviewing^16^ and underwent quarterly feedback and coaching sessions with research staff with motivational interviewing training.

Participants assigned to the control arm received general prevention education from the prevention nurse at each of the 4 in-person visits. All participants were given a copy of the book *Living a Healthy Life With HIV*.^17^ Education sessions for control participants were based on a topic from the book chosen by the participant.

### Randomization and Measures

**Figure 1.**
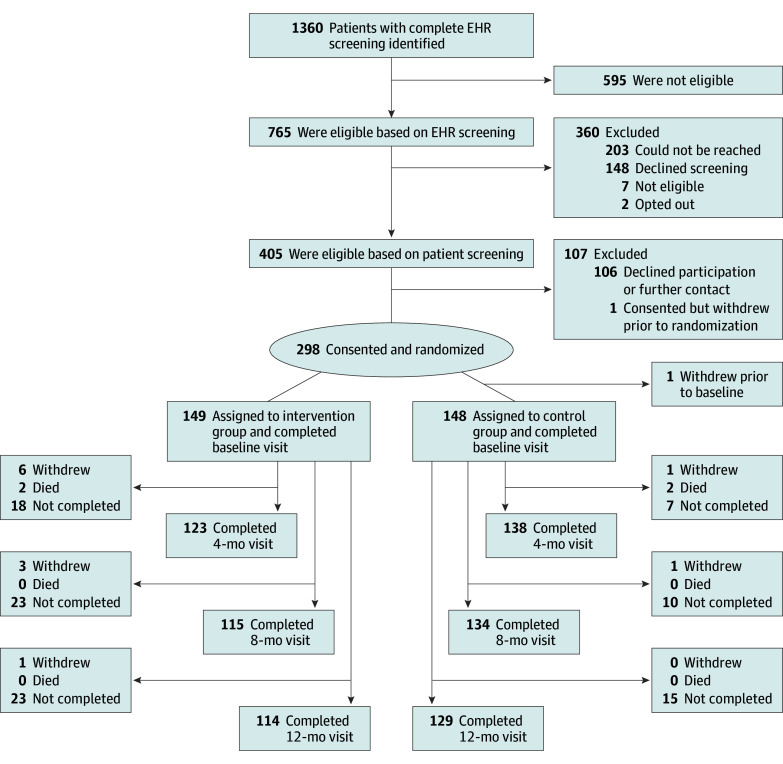
Flow Diagram of Study Participants *Not completed* indicates that no data were collected because the visit was missed entirely. Some participants missed a visit but subsequently came to future visits. Therefore, the total number of participants included in the analyses at each time point was equal to the total number of prior withdrawals and deaths plus the total number of “not completed” for that time point. EHR indicates electronic health record.

The study statistician (K.A.J.) created a 1:1 blocked randomization allocation stratified by trial site. Research assistants were blinded to the allocation scheme. Figure 1 shows the flow of participants.

Patient characteristics (eg, age, sex at birth, and race and ethnicity) were self-reported by participants and obtained from each site’s EHR. Race categories included African American or Black, White, and other (Arab, Asian, American Indian or Alaska Native, Native Hawaiian or Other Pacific Islander, and multiracial); these categories were collapsed into African American or Black, White, and other race categories due to small sample size. Prespecified ethnicity categories included Hispanic or Latino or non-Hispanic or Latino. The EHR data abstraction was performed by research staff. Self-reported race and ethnicity data were collected to calculate 10-year ASCVD risk.

Outcome assessments were conducted at each visit (baseline, 4, 8, and 12 months) by research staff who were blinded to the treatment arm. Systolic blood pressure, the primary outcome, was calculated as the mean of 2 SBP measurements obtained 1 minute apart while the participant rested with feet flat on the floor. Non–HDL cholesterol, the secondary outcome, was calculated as total cholesterol minus HDL cholesterol. Additional lipid fractions were included as exploratory outcomes (total cholesterol, triglycerides, HDL, and low-density lipoprotein [LDL]).

Other prespecified exploratory outcomes included hypertension and hypercholesterolemia treatment cascade metrics, which were defined using EHR and outcome data as 1 of the following mutually exclusive categories: undiagnosed (not listed in EHR problem list), appropriately diagnosed (listed in EHR problem list), appropriately managed (at least 1 medication prescribed for hypertension or hypercholesterolemia), and at treatment goal. Undiagnosed and appropriately diagnosed were combined for the models due to small cell counts. Hypertension treatment goal was defined as SBP lower than 130 mm Hg.^15^ Cholesterol treatment goal was established prior to randomization. Individual goals were generally based on 10-year or lifetime ASCVD risk^18^ but tailored to individual participants at the discretion of the treating clinicians. Study recommended non–HDL cholesterol goal was lower than 130 mg/dL for most participants with low to moderate risk. This recommendation was based on published HIV-specific recommendations from the National Lipid Association,^19^ which were current when we designed the trial. We decided that the recommendation was still reasonable after the publication of the 2018 joint Guideline on the Management of Blood Cholesterol, which defined HIV as a risk enhancer.^20^ A smaller group of high-risk participants, including those with a history of an ASCVD event, were assigned more aggressive treatment goals (<100 or <80 mg/dL).

### Safety and Power

All participants were asked about interim adverse events (AEs) at each study visit, and the prevention nurse reviewed the EHR for any additional information regarding clinical encounters for AEs. Information was uploaded to REDCap (Vanderbilt University) and reviewed by the site-designated clinical co-investigator within 48 hours. Severity and study-relatedness of AEs were adjudicated by the reviewer according to the Division of AIDS Table for Grading the Severity of Adult and Pediatric Adverse Events (corrected version 2.1).

We calculated power using 1000 simulated datasets based on the following assumptions: mean (SD) SBP at baseline of 145 (17) mm Hg, 1-mm Hg decrease in SBP in the control group by 12 months, 15% attrition, and 0.4 within-individual correlation over time. For non–HDL cholesterol, we assumed a mean (SD) baseline of 132 (41) mg/dL and 0.7 within-individual correlation over time. Baseline SBP and non–HDL cholesterol assumptions were based on historical data from the 3 sites. We analyzed the simulated datasets with linear mixed models (LMMs) for each outcome using 2-sided tests and α = .05. We found that given 300 participants, we had over 80% power to detect a greater decrease in SBP of 6 mm Hg and over 90% power to detect a 15-mg/dL reduction in the intervention arm at 12 months compared with the control arm.

### Statistical Analysis

We reported baseline characteristics and outcome values stratified by consent status and treatment status. We used proportions and frequencies for categorical variables and medians and IQRs (quartile 1-quartile 3) for continuous variables. Baseline SBP and non–HDL cholesterol level were reported as means (SDs). Retention was reported overall, by baseline characteristics, and by treatment arm.

Primary analyses were conducted according to the intention-to-treat principle using all available data. We used LMMs to test for differences over time by treatment arm. Models included study visit indicators for each follow-up visit and study-visit-by-treatment-arm interaction terms, which were interpreted as the intervention effect at each follow-up. Site was included as a fixed effect and patient as a random effect to account for within-patient correlation among repeated measurements over time. The treatment cascade outcomes were analyzed using unordered multinomial generalized LMMs with the same fixed and random effects.

While LMMs provide reliable estimates when data are missing at random, we conducted a sensitivity analysis to address differential attrition. We used a generalized LMM to predict attrition at each study visit using baseline characteristics. We then repeated the primary models, including variables that were associated with attrition as additional covariates. Intervention effect estimates were compared with the primary results.

In prespecified exploratory analyses, we examined 3 possible moderators: sex at birth (male or female), ASCVD risk (high or low), and trial site (A, B, or C). For each moderator, we repeated the primary models with the addition of the moderator, moderator-by-study-visit, and moderator-by-study-visit-by-treatment-arm interaction terms. The joint test of the moderator-by-study-visit-by-treatment-arm interaction term was used to identify significant moderating effects. We graphed the sex-specific intervention effects on SBP and non–HDL cholesterol at each time point.

Because some co-investigators were also treating clinicians at the sites, the Data and Safety Monitoring Board requested an analysis of whether having a treating clinician who was also a study investigator moderated the treatment effects. For this analysis, we repeated the primary models stratified by treating clinician status and descriptively compared the results.

Statistical significance was set to α = .05, and clinically significant changes, specified a priori, were considered as a 5-point change for SBP^21^ and a 15-point change for non–HDL cholesterol.^22^ SAS, version 9.4 (SAS Institute Inc) was used for all analyses.

## Results

Participant flow through the trial is described in Figure 1. Of the 405 participants confirmed to meet eligibility criteria after an initial telephone screening, 106 declined to participate and 1 withdrew before randomization. Compared with those who participated, those who declined were younger (mean [SD] age, 53.4 [11.4] vs 57.3 [9.6] years) and less likely to be of Hispanic or Latino ethnicity (1 [0.9%] vs 17 [5.7%]) (eTable 1 in Supplement 2). At each follow-up time point, there was modestly higher attrition observed in the intervention vs control arm.

Baseline characteristics of the 297 randomized participants are described in Table 1 and were well balanced between treatment groups. Overall, participants had a median (IQR) age of 59.0 (53.0-65.0) years; included 62 females (20.9%), 234 males (78.8%), and 1 individual (0.3%) with unreported sex at birth; and identified as being of African American or Black (176 [59.3%]), White (101 [34.0%]), or other (including Arab, Asian, and Native American; 9 [3.0%]), or multiracial (11 [3.7%]) race. One hundred eighty-one participants (60.9%) had public health insurance, and only 100 (33.7%) were currently working. By design, HIV was well treated, with all participants having suppressed HIV-1 viral load and normal or near-normal CD4 T-cell counts. At baseline, 50.2% of the participants (n = 149) were already prescribed 2 or more antihypertensive drugs and 68.0% (n = 202) were taking a statin. Mean (SD) baseline SBP was 135.0 (18.8) mm Hg, and mean (SD) baseline non–HDL cholesterol was 139.9 (44.6) mg/dL (eTable 2 and eFigure 2 in Supplement 2).

**Table 1.** Baseline Patient Characteristics Overall and by Treatment Arm

Variable	Patients, No. (%)		
	Overall (N = 297)	Intervention group (n = 149)	Control group (n = 148)
Enrollment site			
Site A	99 (33.3)	49 (32.9)	50 (33.8)
Site B	100 (33.7)	50 (33.6)	50 (33.8)
Site C	98 (33.0)	50 (33.6)	48 (32.4)
Age, median (IQR), y	59.0 (53.0-65.0)	59.0 (53.0-65.0)	59.5 (52.5-64.0)
Gender identity			
Female	62 (20.9)	35 (23.5)	27 (18.2)
Male	233 (78.5)	113 (75.8)	120 (81.1)
Transgender male, transman, or FTM	2 (0.7)	1 (0.7)	1 (0.7)
Sex at birth			
Female	62 (20.9)	35 (23.5)	27 (18.2)
Male	234 (78.8)	114 (76.5)	120 (81.1)
Unreported	1 (0.3)	0	1 (0.7)
Race^a^			
African American or Black	176 (59.3)	79 (53.0)	97 (65.5)
White	101 (34.0)	60 (40.3)	41 (27.7)
Other race^b^	9 (3.0)	4 (2.7)	5 (3.4)
Multiracial	11 (3.7)	6 (4.0)	5 (3.4)
Hispanic or Latino ethnicity^a^			
Hispanic or Latino	21 (7.1)	14 (9.4)	7 (4.7)
Non-Hispanic or Latino	276 (92.9)	135 (90.6)	141 (95.3)
Marital status			
Married or partnered	68 (22.9)	38 (25.5)	30 (20.3)
Formerly married	54 (18.2)	26 (17.4)	28 (18.9)
Single	172 (57.9)	84 (56.4)	88 (59.5)
Unreported	3 (1.0)	1 (0.7)	2 (1.4)
Sexual orientation			
Gay or lesbian (homosexual)	118 (39.7)	62 (41.6)	56 (37.8)
Bisexual	32 (10.8)	16 (10.7)	16 (10.8)
Straight (heterosexual)	119 (40.1)	55 (36.9)	64 (43.2)
Other sexual orientation^c^	5 (1.7)	3 (2.0)	2 (1.4)
Unreported	23 (7.7)	13 (8.7)	10 (6.8)
Housing status			
No permanent housing	7 (2.4)	2 (1.3)	5 (3.4)
Owns home	121 (40.7)	62 (41.6)	59 (39.9)
Rents home	142 (47.8)	72 (48.3)	70 (47.3)
Staying with friend or family member	19 (6.4)	11 (7.4)	8 (5.4)
Other housing^d^	5 (1.7)	1 (0.7)	4 (2.7)
Unreported	3 (1.0)	1 (0.7)	2 (1.4)
Insurance status			
No insurance	14 (4.7)	9 (6.0)	5 (3.4)
Public insurance	181 (60.9)	85 (57.0)	96 (64.9)
Private insurance	72 (24.2)	41 (27.5)	31 (20.9)
Public and private insurance	25 (8.4)	12 (8.1)	13 (8.8)
Insurance, unknown type	2 (0.7)	1 (0.7)	1 (0.7)
Unreported	3 (1.0)	1 (0.7)	2 (1.4)
Employment status			
Working	100 (33.7)	52 (34.9)	48 (32.4)
Not working	28 (9.4)	16 (10.7)	12 (8.1)
Retired	68 (22.9)	32 (21.5)	36 (24.3)
Disabled (permanently or temporarily)	93 (31.3)	45 (30.2)	48 (32.4)
Student	1 (0.3)	1 (0.7)	0
Other employment^e^	4 (1.3)	2 (1.3)	2 (1.4)
Unreported	3 (1.0)	1 (0.7)	2 (1.4)
Clinical characteristics			
Non–HDL cholesterol target			
80 mg/dL	3 (1.0)	3 (2.0)	0 (0)
100 mg/dL	69 (23.2)	28 (18.8)	41 (27.7)
130 mg/dL	225 (75.8)	118 (79.2)	107 (72.3)
Involved clinician at any point in study period	69 (23.2)	36 (24.2)	33 (22.3)
Most recent CD4 count, median (IQR)	714.0 (510.0-972.0)	696.0 (475.0-915.0)	730.5 (539.0-1001.0)
Most recent viral load, median (IQR)	20.0 (20.0-20.0)	20.0 (20.0-20.0)	20.0 (20.0-20.0)
CD4 nadir, median (IQR)	229.5 (64.5-386.0)	200.5 (49.5-356.5)	248.0 (110.0-440.0)
Antihypertensive medications			
None	48 (16.2)	26 (17.4)	22 (14.9)
Thiazide diuretic	88 (29.6)	45 (30.2)	43 (29.1)
ACE inhibitor	120 (40.4)	53 (35.6)	67 (45.3)
ARB	39 (13.1)	21 (14.1)	18 (12.2)
β-Blocker	95 (32.0)	51 (34.2)	44 (29.7)
Dihydropyridine CCB	98 (33.0)	46 (30.9)	52 (35.1)
Non-dihydropyridine CCB	2 (0.7)	1 (0.7)	1 (0.7)
α2-Blocker	4 (1.3)	1 (0.7)	3 (2.0)
α-Blocker	1 (0.3)	1 (0.7)	0
Hydralazine	6 (2.0)	3 (2.0)	3 (2.0)
Aldosterone antagonist	10 (3.4)	6 (4.0)	4 (2.7)
Other antihypertensive medications^f^	17 (5.7)	10 (6.7)	7 (4.7)
No. of antihypertensive drugs			
0	48 (16.2)	26 (17.4)	22 (14.9)
1	100 (33.7)	47 (31.5)	53 (35.8)
2	85 (28.6)	45 (30.2)	40 (27.0)
3	48 (16.2)	24 (16.1)	24 (16.2)
4	14 (4.7)	6 (4.0)	8 (5.4)
5	2 (0.7)	1 (0.7)	1 (0.7)
Cholesterol-lowering medications			
None	86 (29.0)	46 (30.9)	40 (27.0)
Statin	202 (68.0)	101 (67.8)	101 (68.2)
Ezetimibe	7 (2.4)	3 (2.0)	4 (2.7)
Other cholesterol-lowering medications^g^	24 (8.1)	10 (6.7)	14 (9.5)
ASCVD history			
Prior MI	38 (12.8)	21 (14.1)	17 (11.5)
Stroke or TIA	20 (6.7)	9 (6.0)	11 (7.4)
Other ischemic heart disease	48 (16.2)	26 (17.4)	22 (14.9)
PAD	16 (5.4)	7 (4.7)	9 (6.1)
10-y ASCVD risk score, aged ≥40 y only			
Low	14 (7.3)	10 (9.8)	4 (4.4)
Borderline	15 (7.8)	10 (9.8)	5 (5.6)
Intermediate	98 (51.0)	53 (52.0)	45 (50.0)
High	65 (33.9)	29 (28.4)	36 (40.0)
Other medical history			
Diabetes	89 (30.0)	45 (30.2)	44 (29.7)
Heart failure	20 (6.7)	9 (6.0)	11 (7.4)
Current smoker	85 (28.6)	36 (24.2)	49 (33.1)
Mental health disorder	158 (53.2)	85 (57.0)	73 (49.3)

Abbreviations: ACE, angiotensin-converting enzyme; ARB, angiotensin receptor blocker; ASCVD, atherosclerotic cardiovascular disease; CCB, calcium channel blocker; FTM, female to male; HDL, high-density lipoprotein; MI, myocardial infarction; PAD, peripheral artery disease; TIA, transient ischemic attack.

SI conversion factor: To convert HDL cholesterol to millimoles per liter, multiply by 0.0259.

^a^ Race and ethnicity, along with other characteristics, were self-reported by participants and obtained from each site’s electronic health record.

^b^ Other race included Arab, Asian, and Native American.

^c^ Other sexual orientation included asexual and pansexual.

^d^ Other housing included group home or communal living arrangement.

^e^ Other employment included unpaid household labor and semi-retired.

^f^ Other antihypertensive medications included potassium-sparing diuretic and neprilysin inhibitor.

^g^ Other cholesterol-lowering medications included PCSK9 inhibitor, icosapent ethyl, fibrate, omega-3 fatty acids, fish oil, and bempedoic acid.

**Figure 2.**
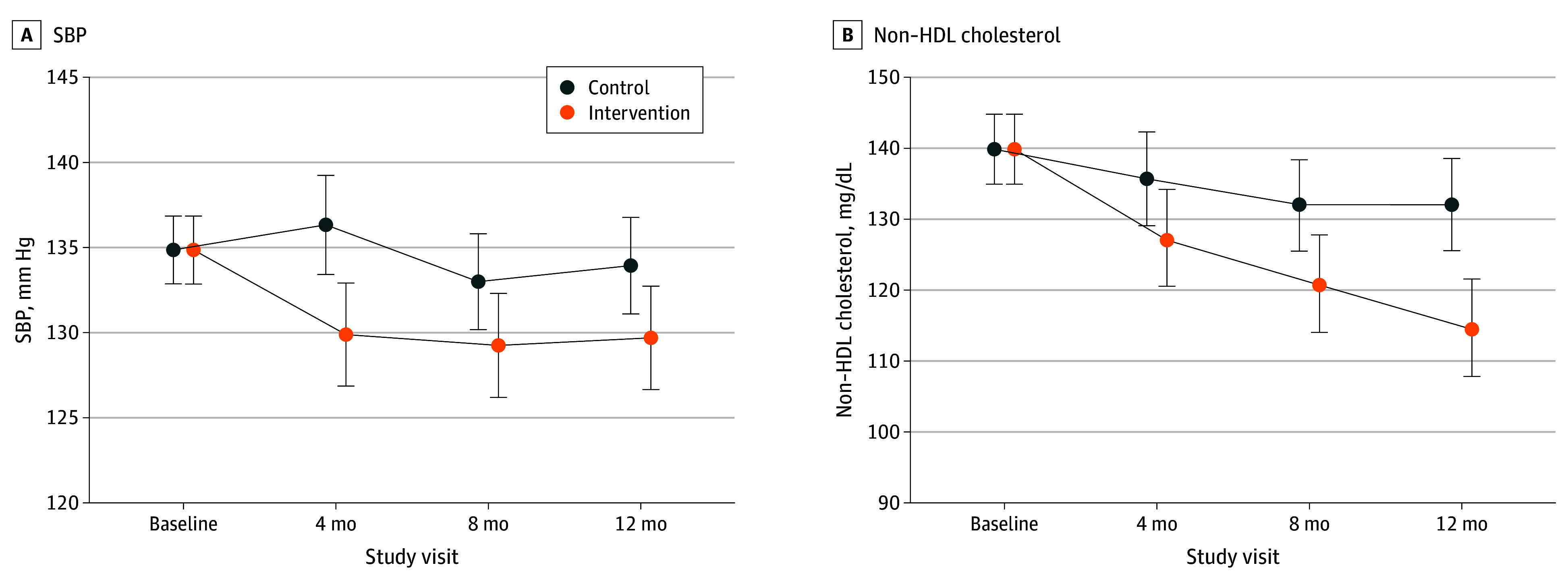
Model-Estimated Systolic Blood Pressure (SBP) and Non–High-Density Lipoprotein (HDL) Cholesterol and Associated 95% CIs of the Effects of the Intervention vs Control Error bars represent 95% CIs.

At 12 months, participants randomized to the EXTRA-CVD intervention arm had 4.2-mm Hg (95% CI, 0.3-8.2 mm Hg; *P* = .04) lower SBP and 16.9-mg/dL (95% CI, 8.6-25.2 mg/dL; *P* < .001) lower non–HDL cholesterol level (Figure 2, Table 2) compared with participants randomized to the control arm. Compared with cholesterol, SBP changed more rapidly, with a 6.4-mm Hg (95% CI, 2.4-10.4 mm Hg; *P* = .002) difference already evident at 4 months. Differences in non–HDL cholesterol steadily widened between groups over the 12-month period.

**Table 2.** Intervention Effects on Primary, Secondary, and Tertiary Outcomes

Outcome	Intervention effect					
	4 mo		8 mo		12 mo	
	Mean difference (95% CI)	*P* value	Mean difference (95% CI)	*P* value	Mean difference (95% CI)	*P* value
**Primary outcome**						
SBP, mm Hg	−6.4 (−10.4 to −2.4)	.002	−3.7 (−7.7 to 0.2)	.06	−4.2 (−8.2 to −0.3)	.04
**Secondary outcome**						
Non–HDL cholesterol, mg/dL	−8.1 (−16.4 to 0.3)	.06	−10.4 (−18.6 to −2.1)	.01	−16.9 (−25.2 to −8.6)	<.001
**Tertiary outcomes**						
Hypertension treatment cascade, OR (95% CI)						
Treated	0.9 (0.3 to 2.6)	.85	1.6 (0.4 to 5.8)	.46	1.3 (0.4 to 4.0)	.61
At treatment goal	1.6 (0.6 to 4.4)	.38	3.0 (0.9 to 10.6)	.08	2.9 (1.0 to 8.3)	.05
Hypercholesterolemia treatment cascade, OR (95% CI)						
Treated	1.2 (0.4 to 4.1)	.74	1.3 (0.4 to 4.9)	.68	1.1 (0.3 to 4.5)	.84
At treatment goal	1.8 (0.6 to 5.1)	.30	3.2 (1.0 to 10.2)	.05	7.3 (2.3 to 23.3)	<.001
**Exploratory outcomes**						
Total cholesterol, mg/dL	−7.3 (−15.8 to 1.2)	.09	−10.0 (−18.4 to −1.5)	.02	−15.5 (−23.9 to −7.0)	<.001
HDL cholesterol, mg/dL	0.9 (−1.3 to 3.2)	.43	1.7 (−0.6 to 3.9)	.15	0.9 (−1.3 to 3.2)	.43
LDL cholesterol, mg/dL	−7.4 (−23.4 to 8.6)	.36	−10.3 (−26.0 to 5.5)	.20	−9.6 (−25.5 to 6.3)	.24
Triglycerides, mg/dL	−12.2 (−36.5 to 12.1)	.33	−34.1 (−58.2 to −10.1)	.006	−29.5 (−53.7 to −5.3)	.02

Abbreviations: HDL, high-density lipoprotein; LDL, low-density lipoprotein; OR, odds ratio; SBP, systolic blood pressure.

SI conversion factor: To convert HDL, LDL, and total cholesterol to millimoles per liter, multiply by 0.0259; triglycerides to millimoles per liter, multiply by 0.0113.

Intervention effects on other tertiary and exploratory effectiveness outcomes are shown in Table 2. Although the intervention arm tended to show improvements in both the hypertension and cholesterol treatment cascades across all time points, the 95% CIs for the point estimates were wide. Compared with the control arm, odds of control in the intervention were only significant for cholesterol at 12 months (odds ratio [OR], 7.3; 95% CI, 2.3-23.3; *P* < .001). Non–HDL cholesterol change was driven by a reduction of 29.5 mg/dL (95% CI, −53.7 to −5.3 mg/dL; *P* = .02) in triglycerides, whereas LDL change was not significant (−9.6 mg/dL; 95% CI, −25.5 to 6.3 mg/dL; *P* = .24). Estimated outcome means and SEs or proportions by treatment arm and time point are available in eTable 3 in Supplement 2.

**Figure 3.**
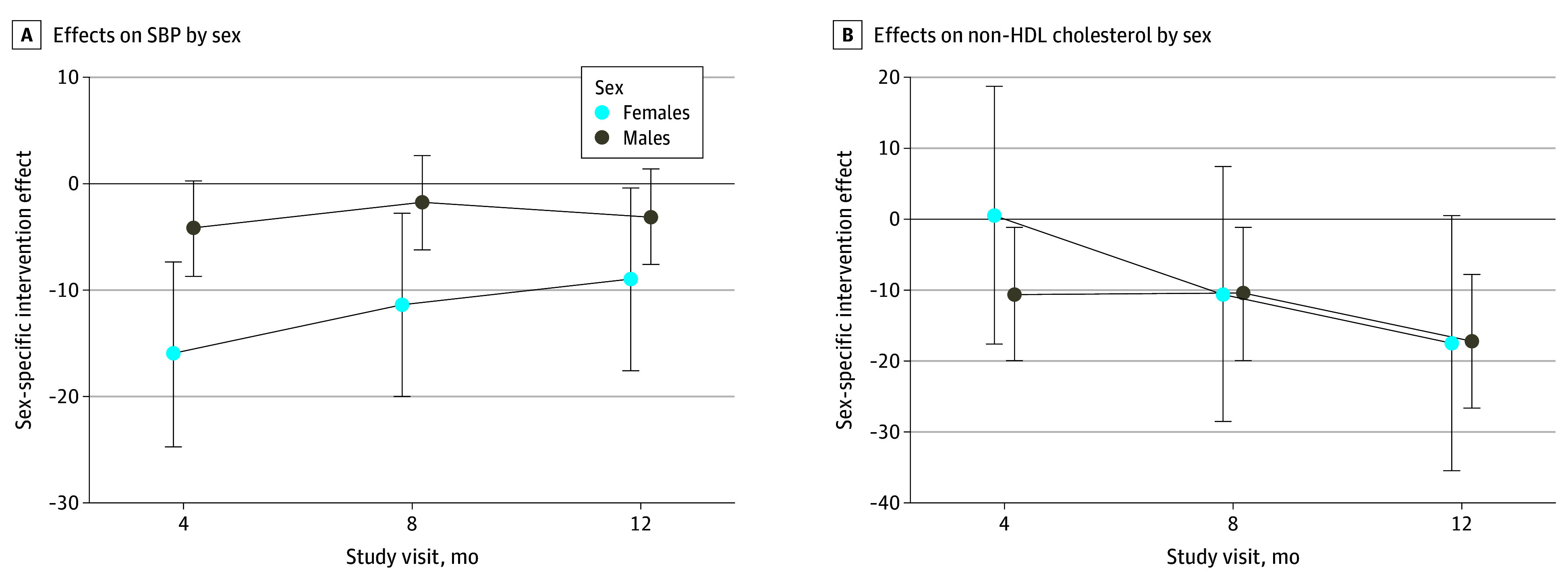
Intervention Effects on Systolic Blood Pressure (SBP) and Non–High-Density Lipoprotein (HDL) Cholesterol by Sex Error bars represent 95% CIs.

In prespecified moderation analyses, we observed a clinically meaningful but not statistically significant difference in SBP effect in females compared with males (11.8–mm Hg greater difference at 4 months, 9.6 mm Hg at 8 months, and 5.9 mm Hg at 12 months; overall joint test *P* = .06) (Figure 3). Other intervention effects were similar by sex (eTable 4 in Supplement 2). Similarly, intervention effects were not consistently different by baseline ASCVD risk or site (eTables 5 and 6 in Supplement 2). Treatment effects were not consistently different for participants whose treating clinician was a study investigator compared with participants with a treating clinician who was not (23% of sample) (eTable 7 in Supplement 2).

There were 232 AEs among 115 participants, similarly distributed by treatment arm (eTable 8 in Supplement 2). Of these AEs, 225 (97.0%) were adjudicated to be unrelated or unlikely to be related to the study. There were 2 unrelated deaths in each arm (2 due to COVID-19, 1 due to sepsis, and 1 due to metastatic cancer). There were 7 possibly, probably, or definitely related AEs, 4 of which occurred in the intervention arm. These AEs included emergency department visits or hospitalizations for high home BP (n = 3), hypotension associated with mild acute kidney injury (n = 1), possible angioedema associated with lisinopril (n = 1), myalgias after restarting statin (n = 1), and an emergency department visit for chest pain (n = 1).

Because of differential attrition between treatment arms, we conducted a post hoc sensitivity analysis in which we adjusted the intervention effect models for factors associated with attrition (eTable 9 in Supplement 2). After adjustment for these factors (sex at birth, race and ethnicity, smoking status, mental health history, insurance status, and employment status), the point estimates for the primary, secondary, tertiary, and exploratory outcomes remained similar to the primary analysis, although 95% CIs were wider and the intervention effect was no longer significant for SBP at 12 months (−3.6 mm Hg; 95% CI, −7.5 to 0.4 mm Hg; *P* = .08).

## Discussion

In this randomized clinical trial, the multicomponent EXTRA-CVD strategy significantly reduced SBP and non–HDL cholesterol level among a diverse population of PWH at 3 HIV clinics. Based on meta-analyses of BP and cholesterol drug trials,^21,22^ a 4.2-mm Hg reduction in SBP and 16.9-mg/dL reduction in non–HDL cholesterol level may be associated with a 14% and 9% decrease, respectively, in clinical ASCVD events.

In the era of more aggressive BP treatment guidelines,^15^ EXTRA-CVD was effective at overcoming clinical inertia to further lower SBP even when the mean BP at study entry was already less than 140 mm Hg, considerably lower than in some BP implementation trials.^23,24,25^ Although providing home BP monitors alone may modestly reduce BP, adding a prevention nurse to facilitate evidence-based and protocolized care in response to home BP measurements further augments the effect, as previously reported in the Take Control of Your Blood Pressure trial^13^ and among PWH in the EXTRA-CVD trial.^14^

In the REPRIEVE (Evaluating the Use of Pitavastatin to Reduce the Risk of Cardiovascular Disease in HIV-Infected Adults) trial, pitavastatin reduced major adverse cardiac events by 35% compared with placebo among PWH with low to moderate ASCVD risk.^26^ As guidelines consider expanding the indication for statin therapy in this population, innovative strategies, such as EXTRA-CVD, will become even more relevant to the implementation of ASCVD prevention care in HIV clinics.

The barriers and facilitators of ASCVD prevention care for PWH, which EXTRA-CVD was designed to address, have been examined.^2,27,28,29,30^ An important barrier in the US is that some HIV clinicians provide comprehensive primary care, whereas other HIV clinicians focus on ART management and other HIV-related care. Evidence suggests that achieving treatment goals may depend on who is managing the BP or cholesterol medications (ie, primary care clinician vs infectious disease specialist).^27^ Fragmented care may lead to poor communication between HIV and non–HIV care practitioners, further fueling PWH’s mistrust in non–HIV clinicians^30^ and leading PWH to value adherence to ART over BP and cholesterol medications.^28^ Yet, there are also opportunities to enhance ASCVD prevention care in US HIV clinics, which may enhance the scalability of EXTRA-CVD. For example, specific funding streams through the federal Ryan White Program may make nurse-led or other ancillary staff–led interventions more financially viable than other primary care settings.^29^

The finding that EXTRA-CVD may be more effective at lowering BP for females compared with males is important given recent epidemiologic evidence that the HIV-associated risk of ASCVD is greater in females than in males.^31^ The reasons for this higher residual risk may be related to both traditional and nontraditional risk factors, such as inflammation and immune activation.^31^ With EXTRA-CVD, females may have been more likely to respond to the frequent communication and additional support from prevention nurses practicing with a holistic health care lens. Gender concordance may have also played a role since all of the prevention nurses in this trial were females. More research is needed to understand why EXTRA-CVD worked particularly well for females and what other prevention approaches are needed to address their residual risk.

Although the EXTRA-CVD intervention was limited to BP and cholesterol, nurse-led case management might be beneficial for a range of other primary care conditions in HIV clinics. If HIV clinics choose to implement EXTRA-CVD, they might consider adding staff trained in other chronic comorbidities and/or health promotion activities. Existing nursing staff may be used or nurses dedicated to non-AIDS comorbidity management may be hired depending on the staffing models, available funding, and local priorities of the clinic.

Just as there may be subgroups of PWH who respond favorably to the EXTRA-CVD strategy, this trial found indirect evidence that some PWH do not respond to and may even disengage from such care. There was a higher rate of attrition in the intervention arm compared with the control arm, and attrition was associated with several demographic factors and social determinants of health. Adjustment for these factors did not substantially change the intervention effect sizes in the models. However, further exploration of these nonresponders is warranted to inform future implementation of EXTRA-CVD or similar care models in HIV clinics.

### Limitations

We acknowledge the limitations of this trial. First, it was conducted at well-resourced, major academic HIV clinics; thus, the results may not be widely generalizable to other populations, such as smaller community-based clinics or HIV care outside the US. Second, the differential attrition in the intervention arm may have introduced bias that may not be fully accounted for in the sensitivity analyses. Although the BP effect size was modestly attenuated in adjusted models, the magnitude of the non–HDL cholesterol effect size was large enough that it was highly unlikely to be primarily due to bias.

## Conclusions

In this trial, the nurse-led multicomponent strategy of care coordination, home BP monitoring, evidence-based treatment algorithms, and EHR tools effectively reduced SBP and non–HDL cholesterol level among PWH with hypertension and high cholesterol at 3 academic medical centers in the US. This finding should inform implementation of multifaceted ASCVD prevention programs for PWH. Future research should explore the most effective components, dose, and mediators of these effects.

## Notes

Supplement 1.
Trial Protocol and Statistical Analysis Plan

Supplement 2. **eFigure 1.** Study Accrual **eTable 1.** Characteristics of People with HIV Who Consented to Participate in EXTRA-CVD Compared to Those Who Declined Participation After an Initial Phone Screen **eTable 2.** Patient Blood Pressure and Cholesterol Values at Baseline, Overall and by Treatment Arm **eFigure 2.** Stacked Bar Chart of Blood Pressure Values at Baseline, by Anti-Hypertensive Treatment Status **eTable 3.** Model-Estimated Outcome Means and Standard Errors or Proportions by Study Arm and Time Point **eTable 4.** Testing Sex as a Moderator of the Intervention Effects on Outcomes by Including an Interaction Term Between Study Arm, Study Visit, and Sex, With Male Sex Compared to Female (Reference Group) **eTable 5.** Testing ASCVD Risk as a Moderator of the Intervention Effects on Outcomes by Including an Interaction Term Between Study Arm, Study Visit, and ASCVD Risk, With High ASCVD Risk Compared to Low/Moderate Risk (Reference Group) **eTable 6.** Testing Site as a Moderator of the Intervention Effects on Outcomes by Including an Interaction Term Between Study Arm, Study Visit, and Site, With Sites 2 and 3 Compared to Site 1 (Reference Group) **eTable 7.** Intervention Effects Stratified by Treating Physician (EXTRA-CVD Investigator or Not) **eTable 8.** Adverse Events by Treatment Arm **eTable 9.** Sensitivity Analysis of Intervention Effects on Primary, Secondary, and Tertiary Outcomes, Adjusted for Baseline Covariates Associated With Attrition

Supplement 3.
Data Sharing Statement
