# Supplementary material for: Nurse-Led Strategy to Improve Blood Pressure and Cholesterol Level Among People With HIV: A Randomized Clinical Trial
Source: JAMA Netw Open. 2024 Mar 5;7(3):e2356445. doi: 10.1001/jamanetworkopen.2023.56445 (PMC10915684; doi:10.1001/jamanetworkopen.2023.56445)
Supplement: Supplement 1. — Trial Protocol and Statistical Analysis Plan [file jamanetwopen-e2356445-s001.pdf]

A nurse-led intervention to extend the HIV treatment cascade for cardiovascular disease prevention (EXTRA-CVD)

Protocol

Version Date: September 7, 2022

ClinicalTrials.gov Identifier:

NCT03643705

Protocol Modifications (more recent changes listed first)

Date	Summary of Changes
**9/7/2022**	• Modified Aim 3 qualitative data collection to specify that the prevention nurses at each site will be recruited to participate in healthcare worker interviews (pp. 46-47).<br>• Revised Aim 3 qualitative interview guide. Added additional questions for prevention nurses regarding motivational interviewing.<br>• Added a protocol to re-engage the design team to inform the development of a composite measure of dose (pp. 31-32 and Appendix T).<br>• Added a new consent form for the new activities related to design team re-engagement.
**10/13/2021**	• Given Dr. Longenecker's physical transition to the University of Washington (UW), Dr. Barbara Gripshover, MD will assume the role as University Hospitals' site PI (p. 7)<br>• Dr. Longenecker's contact information will be replaced by Dr. Gripshover's for UH patient communications and nurse-patient communications of clinically significant information (p. 54), recruitment letters and follow-up letters (local site documents), and informed consent forms (p. 57)<br>• Recruitment goals for AAIM-High (Arm 4) will be updated from 25 per site (75 total) to 35 per site (110 total) (pp. 20-21)
**7/9/2021**	• Added AAIM-High Observation Recording Script to obtain verbal consent for recording visits to later be observed by study staff (p. 43)<br>• Modified approach to Aim 3 qualitative data collection from PLHIV (n-36) and healthcare team members (n=36) to allow for telephone interviews instead of in-person interviews.<br>• Appendix S: Key Informant Interview Guides for PLHIV and Clinicians (p. 117)
**4/12/2021**	• Added Phone Script for Duke Health to obtain verbal consent for access to patient EMR to schedule baseline visit in EMR to allow for better clinic workflow (pp. 22-23)<br>• Added Follow-Up Letter to re patients who are lost to follow up via telephone methods (pp. 31)<br>• Added Appendix Q: AAIM High-specific Process Evaluation Checklist to ensure fidelity of intervention and standardization of study procedures (p. 110)<br>• Added Appendix R: AAIM High clinic context form (p. 114)
**2/12/2021**	• Added plan to enroll subset of aim 2 participants into aim 4 (p. 20)<br>• Added inclusion criteria to aim 4 inclusion/exclusion table (p. 20)<br>• Added virtual consent procedure for aim 4 implementation effectiveness trial, which applies only to participants who were enrolled in aim 2 (p. 23)

	• Added descriptions of finalized aim 4 adaptations decided upon by the design team (p. 46) with forms added as appendices O and P (pp. 106, 107)<br>• Added "Sharing of Results with Research Participants" (pp. 54-55)
**10/29/2020**	• Added a 5th study objective (pp. 4, 12)<br>• Added aim 4 qualitative research methods by study population to table 3 (p. 13)<br>• Added aim 4 to study design (p. 13)<br>• Added enrollment information for aim 4 (p. 18)<br>• Added consent procedures for aim 4 (p. 22)<br>• Added study visit procedures for aim 4 (p. 45)<br>• Added timeline for aim 4 (p. 47)
**6/18/2020**	• Added protocol for motivational interviewing component of nurse clinical skill building into Aim 3<br>• Added language concerning audio recordings for motivational interviewing purposes and patient confidentiality<br>• Added Appendix M: Behavior Change Counseling Index (BECCI) for Measuring Practitioner Motivational Interviewing Skills<br>• Clarification that the window for study procedures during the COVID pandemic (visits, surveys, outcome BP/cholesterol) is +/- 21 days from the "target visit date". The target visit date is calculated as 4-, 8-, or 12-months from the baseline visit (p. 37)
**4/17/2020**	• Added COVID-19 instruments to remote visit procedures<br>• Added table of COVID-19 survey instruments<br>• Added COVID-19 symptom screening and referral to needed services<br>• Added semi-structured interviews for impacts of COVID-19 on self-management of cardiovascular health among PLWH<br>• Added COVID-19 key informant interview guide in Appendix C<br>• Submitted COVID-19 key informant interview telephone script with confirmation of verbal informed consent to participate
**03/24/2020**	• Added protocol for virtual visits during the novel coronavirus outbreak through secure phone calls and/or site-specific HIPAA-compliant online platforms (p. 36)<br>• Submitted three site-specific letters to participants to inform them of changes in study visit format and methods to contact study team during novel coronavirus outbreak
**10/29/2019**	• Added 2 week window for date of in person visits<br>• Added table of BP and cholesterol diagnosis terms for determining cascade category<br>• Added clarification of how many attempts will be made to reach potentially eligible patients by phone<br>• Added additional language on recruitment strategies

	• Added checklist for observation of study visit to ensure fidelity
**9/16/2019**	• Minor changes to statistical analysis in response to NIH review
**9/9/2019**	• Updated BP and Cholesterol treatment protocols<br>• Incorporated process oriented design team recommendations about intervention adaptation.<br>• Added additional detail regarding the aim 3 process evaluation, including the assessment of provider trust and communication ties<br>• Added verbal consent script to obtain age, sex, gender identity, and race/ethnicity from persons who are screened but who decline to participate in the study<br>• Added a recruitment letter and MyChart message (Duke only). Added a telephone script for the follow-up screening call.
**2/7/2019**	• Added additional detail to design team process (sub-aim 1.1)<br>• Added 3 additional consents forms for the design team process<br>o Design team members (focus group and survey)<br>o PLHIV pilot participants (focus groups/pilot intervention)<br>o Feasibility testing among health workers (interview)
**10/29/2018**	• Added clinic variables checklist (p. 18 and Appendix D)<br>• Minor change to wording of inclusion criteria #5 and exclusion criteria #1 related to a simplification of the definition of hyperlipidemia<br>• Minor change to cholesterol treatment algorithm (p. 22) clarifying how the prevention nurse will determine non-HDL goal for the purposes of treatment recommendations.<br>• Added scripts for Aim 1 phone consent for (a) healthcare providers and (b) PLHIV (Duke site)<br>• Added recruitment letter for Aim 1 (Duke site)<br>• Added clinicaltrials.gov registration number
**8/6/2018**	• Added adherence surveys for PLHIV focus group and clinical trial participants

**Title**	**A nurse-led intervention to extend the HIV treatment cascade for cardiovascular disease prevention (EXTRA-CVD)**
**Principal Investigators**	Christopher Longenecker, MD<br>Allison Webel, RN, PhD<br>Hayden Bosworth, PhD
**Grant Support**	National Heart, Lung, and Blood Institute
**Phase**	4
**Population**	Adults living with HIV on antiretroviral therapy with co-morbid hypertension and hyperlipidemia
**Sites**	**1. University Hospitals Cleveland Medical Center**, Special Immunology Clinic, 11100 Euclid Avenue, Cleveland, Ohio, 44106<br>**2. MetroHealth Medical Center**, Infectious Disease Clinic, 2500 Metrohealth Dr., Cleveland, OH, 44109<br>**3. Duke Health,** Infectious Disease Clinic, 40 Duke Medicine Circle, Durham, NC 27710<br>**4. University of Washington,** 1959 NE Pacific St., Seattle, WA 98195
**Duration**	12 months
**Agent or Intervention**	Multi-component health care delivery intervention<br>1. Nurse-led care coordination<br>2. Nurse-managed medication protocols and adherence support<br>3. Home blood pressure monitoring<br>4. Electronic medical records (EMR) support tools
**Objectives**	1. Conduct a baseline assessment of ASCVD preventive care and perceptions of ASCVD risk in the HIV specialty clinic environment.<br>2. Adapt the EXTRA-CVD intervention components to the HIV specialty clinic context with key stakeholder input and data from the baseline assessments<br>3. Evaluate the 12-month efficacy of the EXTRA-CVD intervention to improve BP and cholesterol control in PLHIV<br>4. Conduct a process evaluation of the EXTRA-CVD intervention<br>5. Evaluate the 12-month efficacy of an adapted virtual intervention to improve blood pressure in PLHIV
**Inclusion criteria**	1. **Age ≥18 years**<br>2. **Confirmed HIV+ diagnosis**<br>3. **Undetectable HIV viral load:** defined as the most recent HIV viral load <200 copies/mL, checked within the past year (assessed via chart abstraction)<br>4. **Hypertension**: defined as systolic BP >130 mmHg on ≥ 2 occasions in the past 12 months or on an antihypertensive medication (assessed via chart abstraction), and<br>5. **Hyperlipidemia**: defined as a non-HDL cholesterol >130 mg/dL or on cholesterol lowering medication

**Exclusion criteria**	1. On lipid-lowering medication solely for secondary prevention of ASCVD events with evidence of pre-medication non-HDL which was already below 100mg/dL<br>2. On anti-hypertensive medications solely for a non-hypertension indication (e.g. systolic heart failure),<br>3. Severely hearing or speech impaired, or other disability that would limit participation in the intervention components, and<br>4. In a nursing home and/or receiving in-patient psychiatric care.<br>5. Terminal illness with life expectancy < 4 months<br>6. No reliable access to a telephone<br>7. Pregnant, breast-feeding, or planning a pregnancy during the study period<br>8. Planning to move out of the area in the next 12 months<br>9. Non-English speaking
**Endpoints**	**Primary Endpoints**<br>• 12-month change in systolic blood pressure<br>**Secondary Endpoints**<br>• 12-month change in non-HDL cholesterol<br>• Change in the extended cascade categories [(1) % appropriately diagnosed, (2) % appropriately managed, and (3) % at treatment goal]

Key Roles

Co-Principal Investigator	Chris Longenecker, MD<br>University of Washington<br>ctlongen@uw.edu
Site Principal Investigator	Barbara Gripshover, MD<br>Professor of Medicine<br>Case Western Reserve University<br>University Hospitals Cleveland Medical Center<br>10100 Euclid Avenue, Cleveland Ohio 44106<br>(216)844-5876<br>Barbara.Gripshover@uhhospitals.org
Co-Principal Investigator	Allison R. Webel, RN, PhD<br>University of Washington<br>awebel@uw.edu
Co-Principal Investigator	Hayden Bosworth, PhD<br>Professor of Medicine<br>Duke Department of Population Health Sciences<br>411 West Chapel Hill St, Suite 600, Durham, NC 27701<br>919-286-6936<br>boswo001@duke.edu
Co-Investigators	Corrilynn Hileman, MD<br>MetroHealth Medical Center<br>Cleveland, OH<br><br>Rajesh Vedanthan, MD<br>New York University<br>New York, NY<br><br>Gerald Bloomfield, MD<br>Duke University School of Medicine<br>Durham, NC<br><br>Lance Okeke, MD<br>Duke University School of Medicine<br>Durham, NC
Lead Statistician	Valerie Smith<br>Duke University School of Medicine<br>Durham, NC

1. Background

The HIV/AIDS treatment cascade model was developed to assess how people living with HIV infection (PLHIV) access care and treatment. The model includes sequential steps in care including—(1) diagnosis, (2) prescription of appropriate antiretroviral therapy (ART), and (3) suppression of detectable HIV virus in the blood. These metrics are familiar to HIV-providers and integral to continuous quality improvement initiatives at HIV specialty clinics across the United States, where most PLHIV receive care[1].

PLHIV are known to have a 1.5-2x higher risk of atherosclerotic cardiovascular disease (ASCVD) compared to uninfected individuals, a risk that persists despite viral suppression on ART[2,3]. Thus, once PLHIV achieve the final step of the HIV treatment cascade, providers have an important opportunity to focus on preventing ASCVD and other non-AIDS comorbidities. We envision extending the treatment cascade for high blood pressure (BP) and high cholesterol, which account for much of the population-level ASCVD risk in PLHIV[4], as follows: Step 1, appropriate screening and diagnosis; Step 2, appropriate treatment; and Step 3, achievement of guideline-based treatment targets. Currently, PLHIV are sub-optimally treated for high BP and cholesterol[5-7], possibly due to low perceived risk for ASCVD[8] or challenges in primary care coordination between HIV specialists and non-HIV providers[9]. Non-physician led approaches may address these barriers. Our team has experience testing non-physician led ACSVD prevention interventions in the general population[10-15], including a nurse-led intervention supported by home BP monitoring that lowered systolic BP by 6 mmHg compared to usual care[12].

Our overarching goal is to improve the BP and cholesterol treatment cascade for PLHIV on suppressive ART to reduce ASCVD risk. Guided by a RE-AIM framework (Reach x Efficacy—Adoption, Implementation, Maintenance), and using a mixed-methods clinical effectiveness trial design, our experienced multi-disciplinary team will test a contextually adapted *ASCVD prevention nurse-led intervention (EXTRA-CVD)* to reach guideline-based BP and cholesterol targets. The study will be conducted in three racially and ethnically diverse clinic contexts [University Hospitals (Cleveland, OH), MetroHealth (Cleveland, OH) and Duke Health (Durham, NC)] that are broadly representative of HIV specialty care in the US.

2. Significance

What is the HIV/AIDS treatment cascade? The HIV/AIDS treatment cascade model was developed to assess how people living with HIV infection (PLHIV) access care and treatment. The model examines sequential steps including—(1) diagnosis, (2) prescription of appropriate antiretroviral therapy (ART), and (3) suppression of the HIV virus in the blood. This model led to the ambitious Joint United Nations Programme on HIV/AIDS (UNAIDS) 90-90-90 initiative,[16] with large scale implementation research projects worldwide aiming to achieve these outcomes: 90% of PLHIV who know their status, 90% of those on ART, and 90% of those on ART being virally-suppressed. The cascade metrics are familiar to HIV-providers as mandated core performance measures of the Health Resources and Services Administration (HRSA) of the US Department of Health & Human Services,[17] and are a focus of quality improvement initiatives in HIV specialty clinics across the US. Additionally, supporting research to improve

the HIV cascade is a priority of the NIH as reflected, for example, in the recently released PA-17-194 "Targeted Implementation Science to Achieve 90/90/90 Goals for HIV/AIDS Prevention and Treatment (R01)"[18].

Why extend the treatment cascade for ASCVD prevention? For those who have achieved durable viral suppression (75-80% of patients in our settings), the focus of care should include prevention of non-AIDS comorbidity including ASCVD. PLHIV are known to have a 1.5-2x higher risk of ASCVD compared to uninfected individuals independent of known confounders such as high cholesterol and smoking[2,3]. Although HIV-specific factors play a role, traditional risk factors account for the vast majority of risk on a population level. In an analysis of nearly 30,000 PLHIV from the North American AIDS Cohort Collaboration on Research and Design (NA-ACCORD) study, the top two risk factors with the greatest population level impact on myocardial infarction risk were (1) high cholesterol and (2) high blood pressure, with population attributable risks far exceeding low CD4+ T-cell count or elevated viral load[4]. Unfortunately, uptake of guideline-based therapies for high BP and cholesterol is sub-optimal among PLHIV[5-7].

We envision extending the HIV treatment cascade for two key CVD risk factors—blood pressure and cholesterol—to improve uptake of guideline-based ASCVD prevention therapies. **Step 1**, PLHIV should have their blood pressure and cholesterol screened, and abnormal values should be appropriately diagnosed as hypertension and hypercholesterolemia in the medical record; **Step 2**, those with hypertension or hypercholesterolemia should be prescribed appropriate guideline-based therapies; and **Step 3**, all should achieve guideline-based treatment goals. Developing the HIV treatment cascade for prevention of non-AIDS comorbidities is a logical downstream extension of the treatment cascade paradigm, just as the HIV prevention cascade is extending the treatment cascade further upstream[19] (**Figure 1**).

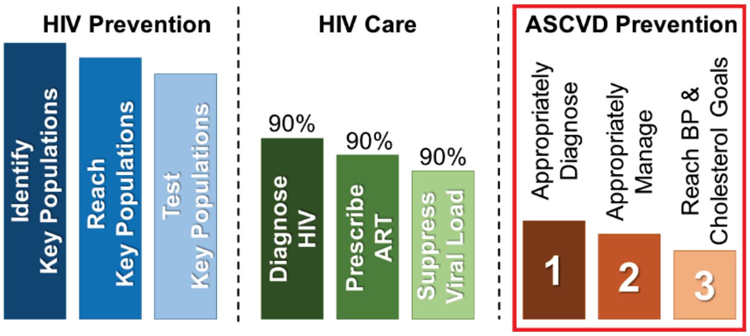

Figure 1: **The extended HIV treatment and prevention cascade**

Blood pressure and cholesterol targets matter. To our knowledge, no HIV-specific blood pressure guidelines exist; however, international guidelines generally agree that treating to a target systolic blood pressure of 140mmHg for most patients is appropriate, while acknowledging that certain groups may merit more aggressive targets (e.g. <130 systolic for diabetes and chronic kidney disease)[20,21]. The most recent AHA/ACC guidelines go further to recommend both pharmacologic and non-pharmacologic strategies to achieve a treatment target of <130mmHg for most patients[22]; although these recent guidelines have yet to achieve widespread

Table 1: **NLA treatment goals for PLHIV.**

Risk Category	Criteria	NLA goal Non-HDL-C LDL-C
Low	N/A*	N/A*
Moderate	2 major risk factors (i.e. HIV + high BP only)	<130 mg/dl<br><100 mg/dl
High	≥ 3 major risk factors	<130 mg/dl<br><100 mg/dl
Very High	Known ASCVD **OR** Diabetes + ≥2 major risk factors *	<100 mg/dl<br><70 mg/dl

Major risk factors include: HIV, Age >45 men or >55 women, family history of early CAD, smoking, hypertension, low HDL-C.
* By design, all participants in our study will be > low risk and those with diabetes will be very high risk.

implementation. To achieve these targets, many will require more than one drug and a lifetime of titrating medication. Improving self-management is thus a critical component to successful treatment of blood pressure over time.[23]

For cholesterol management, HIV-specific guidelines exist[24,25]; although, the National Lipid Association (NLA) recommendations[25] are the only current guidelines from the modern ART treatment era. In 2015, the NLA Expert Panel on HIV recommended the NLA approach to risk stratification and target non-HDL-C and LDL-C goals[26] (**Table 1**), with the additional recommendation that HIV infection may be counted as a major ASCVD risk factor for the purposes of risk stratification (Grade B recommendation, moderate quality evidence). Non-HDL-C (total cholesterol – HDL-C) is recommended by the NLA as a surrogate measure of total atherogenic cholesterol and appropriate treatment target.[26] Critics of cholesterol treatment targets refer to the 2013 ACC/AHA statin treatment guidelines[27] that recommend moderate or high dose statins for four proven statin-benefit groups, citing limited randomized controlled trial evidence for non-statin therapies or cholesterol treatment goals. Subsequent clinical trials, however, have demonstrated that adding certain non-statin treatments such as ezetimibe[28] or PCSK9 inhibitors[29] to statins reduces clinical events, supporting the notion that reductions in risk are generally proportional to reductions in atherogenic cholesterol.[30,31]

The HIV workforce is changing. Unfortunately, achieving ASCVD prevention targets in HIV specialty clinics is challenging in the face of increased clinical demands and a changing HIV workforce. Over the past 10 years, the HIV Medicine Association (HIVMA)[32] and the Institute of Medicine[33] have been warning of looming shortages of HIV specialists. Currently, 58% of HIV providers fit the HIVMA definition of HIV specialist[1], and numbers are projected to decrease due to high levels of dissatisfaction, just as attempts to improve the HIV treatment cascade bring larger numbers of patients into care[1]. Primary care providers (PCPs) may be able to fill the gap, but feel inadequately trained in HIV care.[34] Similarly, HIV-specialists are often uncomfortable providing primary care, including high blood pressure and cholesterol management.[9] Furthermore, HIV-providers who plan to leave practice in the next 5 years are more likely to provide primary care (90%) compared to those who do not plan to leave practice (83%) and those who just recently entered practice (77%, p=0.02).[1] Treating higher numbers of HIV patients is associated with better HIV management and lower overall mortality[35], but is not associated with better cholesterol treatment for ASCVD prevention.[36]

Models that promote shared responsibilities between non-HIV providers and HIV-specialists exist, but their effect on primary care and non-AIDS outcomes such as ASCVD has not been rigorously studied.[37] Undoubtedly, changing patterns of care (i.e. shifting more non-HIV prevention care to non-HIV providers) may require shifts in patient-provider trust and communication. Because of longstanding relationships, many HIV patients fiercely trust their HIV provider for comprehensive care.[9,38] Eighty-four percent of patients preferred having their HIV provider be their PCP[9], and two-thirds of those with an outside PCP only had one because of an insurance company requirement. The impact of patient-provider trust and communication networks on ASCVD prevention efforts needs to be more formally evaluated among PLHIV.

"I always thought I would die of AIDS". Is low perceived risk a barrier to high-quality ASCVD preventive care? Before effective combination antiretroviral therapy, most people with HIV infection died of AIDS-related causes, and cardiovascular disease prevention was not a priority for most patients and providers. Yet, there is some evidence that low perceived cardiovascular risk persists even in the ART treatment era. For example, perceived

CVD risk poorly correlated with Framingham risk scores (r=0.24) among PLHIV with longstanding infection (mean 15 years since HIV diagnosis) and history of lower nadir CD4+ count (mean nadir CD4+ 195)[8]. The reasons for this poor correlation and the influence of perceived risk on ASCVD prevention behaviors are not known.

Nurse-led interventions are highly effective in high-risk populations in the general population. The use of non-physician providers (e.g. registered nurses, nurse practitioners, and physician assistants) is expanding in the US, a trend that is also true in HIV-specialist care.[1,39] The quality of HIV care provided by these non-physician specialists is comparable to physician specialists[40], but the quality of and comfort level with ASCVD preventive care is poorly understood. Our experiences in other US populations suggests that nurse-led management of cardiovascular risk factors is highly effective.[10-12,14] Key features of our prior interventions include: (1) care coordination, (2) nurse-managed protocols and medication adherence counseling, (3) home blood pressure monitoring; and (4) integrated use of information technology tools such as EMR support. For example, home BP monitoring + behavioral counseling led to a 6mmhg reduction in systolic BP in one of our prior studies.[12] Further, a meta-analysis of nurse-managed protocols showed clinically significant 4mmHg reduction in systolic blood pressure and 10-12 mg/dL reduction in cholesterol.[41] In a meta-analysis of adherence interventions, reductions in cholesterol were even higher (15-20mg/dL).[42]

3. Setting and Conceptual Framework

The proposed study will be conducted at three Ryan White Program federally-funded academic medical centers that provide HIV specialty care for racially and ethnically diverse PLHIV that are broadly representative of the US HIV+ population (**Table 2**). The MetroHealth site is primarily urban, while 21% of UH patients and 28% of Duke Health patients are from rural counties. There is minimal overlap in HIV specialty care between Cleveland sites, with preliminary data suggesting <1% receive outpatient HIV care at both sites in a given calendar year. Case Western and Duke have Centers for AIDS Research (CFARs) and considerable support services to conduct HIV research. The Duke and University Hospitals sites are also AIDS Clinical Trials Group sites. Since 2013, PI Longenecker has run an HIV cardiology clinic twice monthly at the University Hospitals Special Immunology Unit HIV clinic, but his patient panel is focused on those with established CVD rather than prevention and risk factor management, highlighting a need for the proposed intervention.

Table 2: Demographics of PLHIV engaged in care at the three academic HIV-specialty clinic sites selected for this study

	Total patients	Age (IQR)	% Female	% Black	% Hispanic
**MetroHealth (Cleveland, OH)**	1759	47 (35-55)	24%	50%	13%
**Duke Health (Durham, NC)**	1890	50 (40-58)	28%	59%	4%
**University Hospitals (Cleveland, OH)**	1101	51 (40-58)	23%	64%	4%

Study Team: Our team is highly qualified to carry out the aims of this study. The three co-Pis represent diverse disciplines and unique research expertise. Co-PI Longenecker is a cardiologist and K23-funded early stage investigator (Year 4) in transition to independence and has an

established track record of research on cardiovascular disease in HIV. Co-PI Bosworth is a senior and awarded PhD psychologist with extensive experience testing interventions to improve cardiovascular risk in vulnerable populations. Co-PI Webel is a PhD nurse scientist and mixed methods researcher who studies self-management of HIV and cardiovascular health among PLHIV. *Our multiple PI structure combines the experience of a senior implementation scientist with the enthusiasm of two promising early stage HIV investigators*. We also incorporate HIV specialists (Hileman and Okeke), cardiologists (Vedanthan and Bloomfield), and statistics and data management support (Smith). Our team has longstanding collaborations and a track record

Figure 2: Conceptual framework for the EXTRA-CVD study

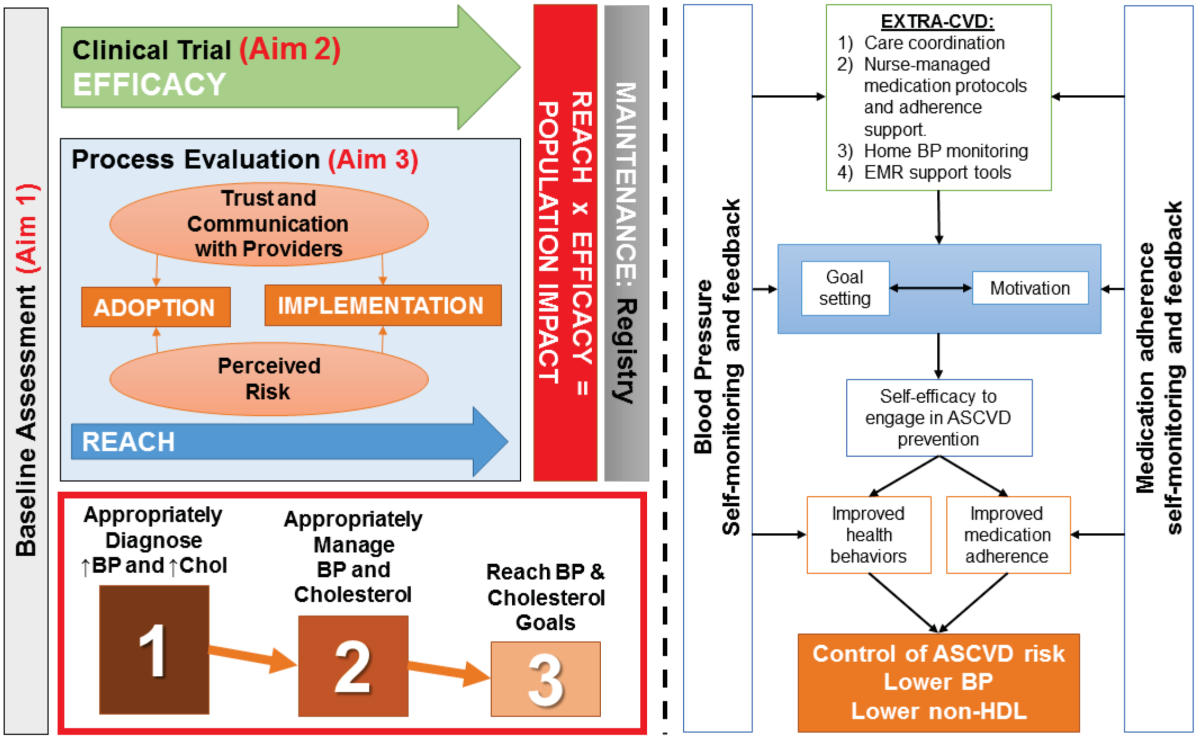

of publication together.[43-54]

Conceptual Framework: Our study utilizes the RE-AIM implementation framework (**Figure 2**). As originally conceived by Glasgow et al[55], RE-AIM stands for **R**each X **E**fficacy—**A**doption, **I**mplementation, **M**aintenance, and captures the five factors that contribute to the public health impact of an intervention. Abrams et al defined population impact as Reach X Efficacy[56], and RE-AIM added the 3 additional components to further describe contextual factors that influence the reach and efficacy of an intervention. We believe that if proven effective for ASCVD risk factor control, the concept of a prevention nurse specialist may be scaled-up to address a broad range of preventive care services for PLHIV, thus increasing its population impact. Our model may be especially relevant in the context of a changing HIV specialty workforce that will increasingly rely on non-physician providers and increased coordination with non-HIV primary care providers and specialists. Finally, the EXTRA-CVD intervention itself is grounded in two models of behavior change: (1) the information-motivation-behavioral skills model and (2) self-regulatory theory.[57-61] These models explain how health behavior change is mediated through self-monitoring (lifestyle change and medication adherence) and acknowledge the central role

that self-efficacy plays in sustained behavior change[62-64] (**Figure 2 sidebar**).

4. Objectives

1. Conduct a baseline assessment of ASCVD preventive care and perceptions of ASCVD risk in the HIV specialty clinic environment. (**Aim 1**)
2. Adapt the EXTRA-CVD intervention components to the HIV specialty clinic context with key stakeholder input and data from the baseline assessments (**Sub-Aim 1.1**)
3. Evaluate the 12-month efficacy of the EXTRA-CVD intervention to improve BP and cholesterol control in PLHIV (**Aim 2**)
4. Conduct a process evaluation of the EXTRA-CVD intervention (**Aim 3**)
5. Evaluate the 12-month efficacy of an adapted virtual intervention to improve blood pressure in PLHIV

5. Hypothesis

We hypothesize that our prevention nurse-led intervention will lead to a statistically significant reduction in systolic BP (primary) and non-HDL cholesterol (secondary) over 12 months compared to those receiving general prevention education only.

6. Study Design.

<u>Aims 1</u>: **Mixed-methods**

There are two groups of participants for this aim

- **PLHIV (n=60)**
- **Healthcare team members (n=36)**.

This mixed-methods aim will have two complimentary areas of focus:

- **Perceptions of ASCVD risk**
- **Barriers to and facilitators of high-quality ASCVD preventive care**

We will recruit up to **60 PLHIV** (*n*=20 from each site) to participate in a mixed-methods study. We will enroll until we achieve data saturation, where no new themes or explanations emerge. Based on previous work[49,65-68], this sample size more than sufficient to reach saturation. All PLHIV will have suppressed HIV viral load (<200 copies/ml), hypertension (SBP >130mmHg on two occasions in the EMR within the last year and/or on anti-hypertensive medication), <u>AND</u> hypercholesterolemia (non-HDL > NLA target (see **Table 1**) and/or on cholesterol medication). Additionally, up to **36 healthcare team members** (3 HIV MDs, 3 PCPS, 3 RNs and 3 support staff members from each site) will complete a key informant interview. PLHIV and care team members will be recruited by purposive and snowball sampling from the clinics in which they receive care and work. All subjects will be consented prior to enrollment.

<u>Sub-Aim 1.1</u>: **Intervention adaptation**

We will use a participatory, iterative design process[69-71] with a "design team" at Cleveland

(combined UH/Metro Design Team; ~12 members) and Duke (~6 members). Design team members may include research team members, HIV providers, primary care providers, nurses, PLHIV with hypertension and hypercholesterolemia, information technology experts, and any other key stakeholders TBD. The design process will involve three main phases: brainstorming, conceptualization, and creation described below in study procedures. Additional acceptability and feasibility testing will occur before and after a 6 week pilot intervention. These data will inform two additional design team meetings to iteratively refine the intervention.

Aim 2: Randomized controlled trial

HIV+ adult participants (n=300) on suppressive ART with both hypertension (confirmed systolic BP >130 mmHg and/or on treatment) & hypercholesterolemia (non-HDL cholesterol > National Lipid Association targets and/or on treatment) will be stratified by 3 clinic sites and randomized 1:1 to intervention vs. education control.

Aim 3: Descriptive Process Evaluation

We will conduct a process evaluation[72] of the intervention focusing on the RE-AIM domains of ***reach***, ***adoption***, and ***implementation***. We will evaluate key implementation process measures across the following domains: fidelity (quality), dose delivered (completeness), dose received (exposure and satisfaction), recruitment, reach (participation rate), and context with both PLHIV and health care team participants[73,74]

**Table 3: Summary of qualitative research methods by study population**		
Population	Target N	Methods
Aim 1: PLHIV	60	Focus group or interviews*
Aim 1: Healthcare Workers	36	Interviews
Aim 1.1: PLHIV Pilot participants	9	Focus group or interviews*
Aim 1.1: Feasibility Testing (Healthcare Workers)	9	Interviews
Aim 1.1: Design Team Members	20	Focus group
Aim 3: Process Evaluation (PLHIV)	30	Interviews
Aim 3: Process Evaluation (Healthcare Workers)	36	Interviews
Aim 4: Design Team Members	20	Focus group or design meeting transcripts
Aim 4: PLHIV	24	Interviews

Aim 4: Virtual Intervention Adaptation and Hybrid Implementation-Effectiveness Study

Intervention Adaptation

The adaptation process will be similar to that described above for sub-aim 1.1, however, will be condensed into three sessions: one at-home preliminary activity and two Zoom videoconference meetings. At these meetings, several suggested virtual enhancements will be presented and design team members will engage in abbreviated exercises to (1) brainstorm, (2) conceptualize, and (3) create new virtual enhancements to the intervention. Small working groups will be assigned to further develop the enhancements into final products offline, using iterative

processes consistent with human-centered design principles

Implementation-Effectiveness Trial

In this hybrid type 3 implementation study, we will enroll (n=75) adult PLWH on suppressive ART with high BP whom are otherwise ineligible for the parent trial because they do not also have high cholesterol or because they are unwilling or unable to participate in the in-person trial. Participants will be stratified by the 3 clinic sites. Implementation outcomes based on a RE-AIM framework[8] will be compared to parent trial participants: **reach** (% agreeing to participate), **effectiveness** (change in home systolic BP), **adoption** (frequency of home BP use), **implementation** (qualitative assessment of feasibility/acceptability), and **maintenance** (qualitative).

7. Study Enrollment and Withdrawal

Aim 1 Enrollment:

There are two groups of participants for Aim 1, PLHIV and Healthcare Team Members. We will recruit PLHIV participants using an IRB-approved flyer describing the study and a number to call for information as well as by presenting at staff meetings and retreats. In addition, the Duke site will additionally use an IRB-approved recruitment letter. We will recruit Healthcare team members at each study site by email and personal invitation, attempting to obtain a representative sample. We will enroll 3 HIV MDs, 3 PCPS, 3 RNs and 3 support staff members from each site (see table)

After a brief screening, potential subjects not meeting inclusion criteria will be thanked for their time and excluded from enrollment. Those meeting criteria (see table below) will be scheduled for the enrollment visit and interview.

* Our inclusion criteria for PLHIV participating in aim 1 matches the enrollment criteria of PLHIV for aim 2, with the idea that we would like to recruit these same participants to enroll in the clinical trial portion of the study; however, this aim 1 study will be temporally separated from aim 2 and subjects will not be required to participate in both aims of the study. The reason for this is to gather data on a broadly representative and generalizable sample of PLHIV with hypertension and hypercholesterolemia prior to intervention adaptation and implementation.

* Our hypertension inclusion criteria reflect the definition of hypertension from the recently updated AHA/ACC Guideline for the Prevention, Detection, Evaluation, and Management of High Blood Pressure in Adults[22].

	Inclusion Criteria	Exclusion Criteria
**PLHIV (n=60)**	1. Age ≥18 years<br>2. Confirmed HIV+ diagnosis<br>3. Undetectable HIV viral load: defined as the most recent HIV viral load <200 copies/mL, checked within the past year (assessed via chart abstraction)<br>4. Hypertension: defined as systolic BP >130 mmHg on ≥ 2 occasions in the past 12 months or on an antihypertensive medication (assessed via chart abstraction)<br>5. Hyperlipidemia: defined as a non-HDL cholesterol >130 mg/dL or on cholesterol lowering medication	1. On lipid-lowering medication solely for secondary prevention of ASCVD events with evidence of pre-medication non-HDL which was already below 100mg/dL<br>2. On anti-hypertensive medications solely for a non-hypertension indication (e.g. systolic heart failure)<br>3. Severely hearing or speech impaired, or other disability that would limit participation in the intervention components<br>4. In a nursing home and/or receiving in-patient psychiatric care<br>5. Terminal illness with life expectancy < 4 months<br>6. No reliable access to a telephone<br>7. Pregnant, breast-feeding, or planning a pregnancy during the study period<br>8. Non-English Speaking
**Healthcare team members (n=36)**	1. HIV specialist MD (generalist or infectious disease) with at least 1 full day of outpatient clinic/week, OR<br>2. Primary care provider who cares for >5 HIV+ patients in his/her panel, OR<br>3. Registered nurse whose primary appointment is in an HIV specialty clinic, OR<br>4. Support staff member at an HIV specialty clinic including but not limited to: medical assistant, receptionist, dietician, social worker, or pharmacist.	1. PI or co-investigator on the study<br>2. Non-English Speaking

Sub-Aim 1.1 Enrollment:

There will be 2 design teams—one in Cleveland (~10-12 participants; combined UH and Metro) and one at Duke (~6-8 participants). Each design team will be composed of willing participants recruited from the local clinics and may include research team members, HIV providers, primary care providers, nurses, PLHIV with hypertension and hypercholesterolemia, information technology experts, and any other key stakeholders. The intervention design team process is not a research study; however, participants will be asked to participate in a research evaluation of the design process. This will consist of a brief survey and a focus group discussion. Potential participants for the survey and focus group discussions will be recruited at the design team meetings, but participation in the research component will not be a requirement for participation in the design team process.

In addition, we will recruit up to 3 PLHIV at each clinic site (up to 9 total) to participate in a 6-week pilot study of the intervention (all will be assigned to the intervention assessments as described in aim 2 for this pilot). In order to provide feedback that will help refine the intervention further, these pilot participants will be asked to participate in focus groups and/or interviews before and after the pilot study. These PLHIV will fulfill the same inclusion/exclusion criteria as for the randomized trial described in Aim 2. They will be recruited ***in person*** from the patient panels of study investigators or HIV-specialty clinic colleagues (with their permission) at the time of their usual clinic appointments. Because we are recruiting such a small number of subjects, we expect that we will not need any additional recruitment materials for this part of the study.

We will also involve our local HIV Community Advisory Boards (CAB) at UH and Duke to provide feedback on our study during the design team process. These CABS are routinely utilized in many HIV studies. At the CAB meetings, our study staff will be taking notes about content themes and specific suggestions but the sessions will not be recorded and none of the recorded data will be associated with CAB member identifiers. Because there is no identifiable data being collected, we are asking for a waiver of consent for these CAB meetings.

Finally, we will also conduct interviews of up to 18 health workers recruited from the HIV clinic sites (6 per site) who may have come into contact with the prevention nurse or pilot participants' care during the course of the 6 week pilot. Examples of such health workers may include: (a) a primary HIV doctor who is asked by the prevention nurse to add a new blood pressure medication, (b) an HIV nurse who interacts with the prevention nurse to coordinate care between HIV provider and PCP over a cholesterol management issue. Potential participants in this part of the pilot evaluation will be identified through the course of the pilot intervention by the site prevention nurse and will be recruited from the clinics by the prevention nurse. At all three sites, we intend to recruit these persons from clinic through in-person invitations to participate in a feasibility testing interview. Once recruited, arrangements for an in-person consent and interview will be made.

Aim 2 Enrollment:

Pre-screening: We will use the electronic medical records at our three sites to identify potential subjects for our study according to the following inclusion/exclusion criteria (same as

aim 1 PLHIV, except for addition of exclusion criteria #8). Potential subjects will initially be mailed a recruitment letter signed by his or her primary HIV provider (or sent a message through MyChart at Duke site only, see below). Potential subjects will have the opportunity to opt out of the study by calling a toll-free number. We have used these same strategies in our previous studies.

	Inclusion Criteria	Exclusion Criteria
**PLHIV (n=300; n=100 per site)**	1. Age ≥18 years<br>2. Confirmed HIV+ diagnosis<br>3. Undetectable HIV viral load: defined as the most recent HIV viral load <200 copies/mL, checked within the past year (assessed via chart abstraction)<br>4. Hypertension: defined as systolic BP >130 mmHg on ≥ 2 occasions in the past 12 months or on an antihypertensive medication (assessed via chart abstraction), and<br>5. Hyperlipidemia: defined as a non-HDL cholesterol >130 mg/dL or on cholesterol lowering medication	1. On lipid-lowering medication solely for secondary prevention of ASCVD events with evidence of pre-medication non-HDL which was already below 100mg/dL<br>2. On anti-hypertensive medications solely for a non-hypertension indication (e.g. systolic heart failure)<br>3. Severely hearing or speech impaired, or other disability that would limit participation in the intervention components<br>4. In a nursing home and/or receiving in-patient psychiatric care<br>5. Terminal illness with life expectancy < 4 months<br>6. No reliable access to a telephone<br>7. Pregnant, breast-feeding, or planning a pregnancy during the study period<br>8. Planning to move out of the area in the next 12 months<br>9. Non-English Speaking

MyChart is the patient portal of the electronic medical record at Duke (Epic). For patients that utilize Epic MyChart messaging at Duke, a Maestro Care MyChart recruitment invitation will replace the recruitment letter. The DOCR Maestro Care Analyst team is involved in implementing this process. Potential subjects who fit basic inclusion criteria are identified by a computer algorithm within the electronic medcial record (Epic) based on diagnoses, recent lab values, demographic information, and blood pressures. No person will review the medical record and no information will be shared with the research team prior to this message being sent. If the

message is received and read by the patient, he/she will indicate if they are interested or not interested and the study coordinator will be sent an In basket message (Maestro Care internal message) indicating the response. Only key personnel who are delegated the task of patient identification and recruitment will have access to the In basket messages. Patients who express interest or patients who do not respond will be contacted by telephone following the screening process described below.

Screening: A research assistant will contact all subjects who do not opt out. Following a telephone script, the research assistant will describe the study in detail, ensure the patient is eligible and willing to participate, and schedule a baseline study visit at the next clinical visit with an HIV provider where they will be enrolled following the informed consent process described below. Study staff will make up to 3 attempts at telephone contact before marking a pre-screen eligible subject as "unable to contact." In person contacts may be made at scheduled clinic visits. We will also seek to ensure that the contact information on file is correct by cross-referencing other sources in the electronic health systems at each site.

Finally, some patients may become aware of the study through their care team or word of mouth prior to receiving a pre-screening letter. These patients will be encouraged to contact study staff for screening (same script as above). In addition, a flyer will be posted at the Duke Infectious Diseases clinic as an additional avenue of recruitment. Additional across the board or site-specific strategies may be considered in the future if recruitment is a challenge.

We have previously estimated (2016 data; see table) that 900+ subjects in care at the 3 study sites would meet our inclusion criteria and would be eligible for enrollment. If we have trouble recruiting, we have access to additional community sites associated with our academic medical centers with similar demographics. Minority populations will be enrolled. While this group is considered a vulnerable population, the study team has considerable experience enrolling these participants and adapting interventions that are culturally sensitive. Given the increased prevalence of ASCVD risk factors in these populations, it is important that they are not excluded from participation in this study.

Table 4: Estimated eligible subjects at each clinic site based on 2016 data.

	HIV viral load <200	Hypertension*	Hypercholesterolemia*	Both*
MetroHealth	1500 (85% of all HIV+)	491	501	**286**
Duke	1349 (71%)	605	397	**291**
University Hospitals	975 (89%)	550	485	**334**

* Defined here as a billing code/chart diagnosis OR on anti-hypertensive or cholesterol medication. The numbers for hypertension and hypercholesterolemia reflect ONLY HIV patients with HIV viral load <200 copies/ml.

Aim 3 Enrollment:

Using the same methods as in Aim 1 (IRB-approved flyer and personalized invitations), random subset of *n*=30 intervention participants (10 from each of the three sites) and *n*=36 healthcare team members will be asked to complete key informant interviews. We will target the same 36 care team members who completed aim 1 interviews but, due to staff turnover, it is possible we may enroll those who did not participate in Aim 1.

An important process measure of Aim 3 is intervention dose, yet the total dose of complex multi-component interventions is often difficult to conceptualize. As part of a Diversity Supplement of the parent grant awarded to Dr. Angela Aifah, we will re-engage the design team members that have participated in sub-aim 1.1 and aim 4 intervention adaptations to participate in a 2-part process of constructing a composite measure of total dose as described below and in Appendix T.

Aim 4 Enrollment:

Intervention Adaptation

Participants in the adaptation design team have already been identified as described in sub-aim 1.1, and have participated in the adaptation of the aim 2 trial. All original 20 design team members agreed to be contacted for future research, and will therefore be invited to participate in the aim 4 intervention adaptation process. Those members who agree to participate in aim 4 will be consented virtually and sent an invitation to participate in two zoom meetings, as described in the study procedures section.

Implementation-Effectiveness Trial

The aim 4 implementation study will enroll 110 participants (n=35 at each site) in the virtual arm. We have previously estimated the number of eligible participants at each site (Table 5). These numbers demonstrate the feasibility of our proposed new enrollment (~10x the enrollment goal).

Table 5: Estimated eligible participants at each clinic site.

	High BP	High Cholesterol	Both*	High BP *only**
MetroHealth	491	501	286	**205**
Duke	605	397	291	**314**
University Hospitals	550	485	334	**216**

* Those with both conditions are eligible for the parent trial, and those with high BP only are eligible for the virtual arm.

PLWH on ART with suppressed HIV viral load and high BP will be eligible to enroll in the virtual intervention arm if they are otherwise not eligible for the parent trial because they do not also have high cholesterol. In addition, any patient who would otherwise be eligible for the parent trial, but who is unwilling or unable to come to the clinic site for in-person visits would be offered an opportunity to enroll in the virtual arm. These data will be captured in our detailed screening and enrollment form. The inclusion and exclusion criteria will otherwise remain the same as the parent trial, including access to a telephone. Access to a computer or smart phone with video camera teleconferencing capabilities will not be required, but will be closely

tracked and high levels of support for using these technologies will be provided as described below. Data from our sites indicate that over two-thirds of participants will have access to videoconferencing technology.

Participants for this virtual intervention arm of aim 4 will be screened and recruited through the same methods described in aim 2 if they meet aim 2 eligibility criteria 1-4. In addition, those participants identified as eligible for aim 2 but are unable or unwilling to enroll in the parent study due to issues related to access, will also be invited to enroll in this virtual arm through the same methods described in aim 2.

Additionally, since this trial is focused on implementation outcomes, each site will include a subset of interested parent trial (aim 2) participants. Each site will aim to enroll at least five but no more than ten participants who have completed the aim 2 trial. The goal is to gather enough data to determine if the aim 4 virtual intervention provides support to participants in maintaining and perhaps improving upon their blood pressure management achieved in the parent trial.

	**Inclusion Criteria**	**Exclusion Criteria**
**PLHIV (n=110; n=35 per site)**	1. Age ≥18 years<br>2. Confirmed HIV+ diagnosis<br>3. Undetectable HIV viral load: defined as the most recent HIV viral load <200 copies/mL, checked within the past year (assessed via chart abstraction), and<br>4. Hypertension without hyperlipidemia (both defined in parent trial), or<br>5. Hypertension and hyperlipidemia but unable/unwilling to enroll in the parent trial.<br>6. Previously enrolled in the parent trial and interested in improving hypertension management through a virtual format.	**Same as parent trial criteria** outlined in aim 2.

<u>Withdrawal:</u>

Throughout all phases of our study, subjects will be encouraged to complete the full course of the study assessments. However, it is understood that a subject may discontinue study

participation at any time for any reason. The reason for early withdrawal must be documented in the subject's case file and in the subject tracking document.

Reasons for Withdrawal: Subjects are free to withdraw from the study at any time for any reason. Subjects should normally be withdrawn from the trial if a serious adverse event (SAE) occurs. Subjects **must** be withdrawn from the trial if:

1. They withdraw their consent;
2. The investigator considers it in the best interest of the subject that he or she is withdrawn;

The reason for any subject's discontinuation and the date of withdrawal will be recorded in the subject's case file. The subject's case file, which will be completed up to the point of withdrawal, will be retained for three years. The study report will include reasons for subjects' withdrawals as well as details relevant to the subjects' withdrawals. Any subject withdrawn from the trial prior to completion will undergo all procedures indicated in this protocol as being scheduled to occur at discharge or upon early withdrawal. Any subject withdrawn due to an adverse event (whether serious or non-serious) or any clinically significant abnormal laboratory test value will be evaluated by the Principal Investigator or a monitor (see Key Personnel), and will be treated and followed up until the symptoms or values return to normal or acceptable levels, as judged by the Principal Investigator. Relevant post-study procedures will be performed, wherever possible, on subjects who elect to withdraw.

Handling of Withdrawal: If a subject is withdrawn from participation in the study at any time at his or her request, at the IRB or Principal Investigator's discretion, the reason(s) for discontinuation shall be documented thoroughly in the source documents and subject's case file. If a subject is discontinued because of an adverse event, this event will be followed until it is resolved or the subject is clinically stable and will also be documented in the source documents and the subject's case file.

8. Study Procedures

Informed consent:

Subjects for each aim will give informed consent to participate in the project. Research Assistants will read, review, and discuss consent forms with all potential participants prior to asking them to sign. If the candidate appears confused or indicates a lack of understanding, the interviewer will attempt to identify the misunderstanding and to explain the form again. Any candidate who still does not comprehend the form will be excluded from the study. We will ask questions to confirm understanding of the material covered in the consent procedure, both open-ended (e.g., "Could you tell me what's going to happen if you enroll in the study?") and closed (e.g. "Will you get free medications from the staff of this research study?" or "what will happen to your medical care after the study?"). Persons who understand the consent form and agree to participate in the study will be asked to sign an authorization for the release of medical information to us. Interviewers will witness and date the signed forms and complete the corresponding. An informed consent checklist will be used to document the participant's understanding of the informed consent process. Consent procedures will take place in a private room or office. Consent forms will be kept in a locked file cabinet within a locked room.

For sub-aim 1.1, we will consent pilot participants once prior to acceptability testing

interview/group discussion and this consent will cover the entire pilot process (acceptability testing, pilot trial procedures, and feasibility testing). For this sub-aim 1.1, we will also be soliciting feedback on the trial design from local HIV Community Advisory Boards (CAB) at UH and Duke as described above. Because there is no identifiable data being collected, we are asking for a waiver of consent for these CAB meetings.

For aim 1 and sub-aim 1.1, some interviews at the Duke site will be conducted by telephone in order to reduce barriers to enrollment and therefore obtain the most representative sample possible. These phone consents will be scripted (see separate IRB-approved scripts for Aim 1 and sub-aim 1.1), adhering to the same general consent process described above. Phone consents will NOT be performed at UH or MetroHealth sites, because travel is less of a barrier for these clinic populations.

For aim 2, a partial waiver of consent is requested for pre-screening and chart review procedures as described. Once the subject is screened and agrees to enroll, then informed consent will be obtained at the baseline visit. Prior to the baseline visit, in order to streamline data collection for the participant and study staff, the study staff will perform a chart review of the participant's medical history. Medical history obtained from chart review will be verified with the participant on the day of the baseline visit. Our rationale is that we anticipate a low risk for loss of confidentiality during pre-screening and chart review procedures. In addition, waiting to perform the chart review together with the participant on the day of the procedure would considerably lengthen the study visit, which would inconvenience the participant. If, in the rare case that a qualified participant agrees to participate and is scheduled for a baseline visit, but then fails to attend the baseline visit and decides not to reschedule prior to signing informed consent, then any information collected by chart review will be deleted from the study database.

We plan to obtain verbal consent to record the following demographic variables for all persons that agree to be screened in person or by phone, but who ultimately decline to participate: (1) age, (2) sex, (3) gender identity, (4) race/ethnicity. Thus, at the end of the trial, we will be able to document whether those who declined to participate are different demographically than the population of patients who agreed to enroll. This is important to describe the generalizability of our study findings. Verbal consent will be documented in RedCap. Verbal consent will also be obtained and documented for scheduling and documenting participants' baseline visits in participants' Electronic Medical Records at Duke Health.

For the aim 4 intervention adaptation, the 20 original design team participants who agreed to be contacted for future research in the consent form for sub-aim 1.1 will be invited to participate in the aim 4 virtual design sessions. These design team members agreed to be contacted and engage in communication with study team members via e-mail during sub-aim 1.1. To invite design team members to participate in aim 4, the study team coordinators at each site will send an e-mail to members explaining the purpose and procedures of the adaptation activities. This e-mail will include a REDCap survey link to a form that will capture the participant's interest in participating in or declining the invitation to aim 4. For those that decline, the REDCap survey will end, thanking the member for their time. For those that are interested in participating, the REDCap survey will inform the design team member that a study team member will need to contact them to complete the informed consent process, and the survey will capture the preferred communication method to schedule that virtual visit (e-mail or phone contact information). A study team member will then contact the design team member

through phone or e-mail to schedule the virtual informed consent visit and explain the process. A study team member will email a REDCap survey link to the full version of the consent document to the participant on the scheduled date. This virtual consent will capture an electronic signature and follow most of the guidelines laid out by the UH IRB in their document titled *Guidelines for Remote Electronic Consent*. An exception will be the audiovisual visit to confirm identity, as the study team has determined that this will place unnecessary burden on the design team members. All design team members have worked closely with the study team during sub-aim 1.1 and their identity is well-known to the study team. Aligning with data captured from participants during sub-aim 1.1, the study team member will ask the participant for their full name, professional title at their respective site, and their e-mail address to confirm their identity over the phone, and will then perform verbal informed consent while the participant reads along through the REDCap survey link. The participant will select in REDCap whether or not they would like to be contacted for future research, how their data may be used, and will provide their electronic signature confirming their consent to participate. The design team sessions will take place via Zoom videoconference, and these sessions will be recorded for the purpose of qualitative data analysis. Using a recording disclaimer function available in Zoom, attendees will be prompted to provide their consent to be recorded before entering the Zoom session. If the participant agrees, they will enter the Zoom session, and if they decline, they will not be able to enter the Zoom session. Neither sub-aim 1.1 nor aim 4 discuss or share confidential information through e-mail or in-person meetings. The only participant level data that will be collected from design team members is demographics, which will be collected after they consent through a REDCap survey link. That link will also include an organizational readiness assessment for those whose professional role is within the study site HIV clinic. Design team Zoom activities are centered solely on the intervention design and study implementation procedures, and therefore the Zoom videoconference will not put design team participants at risk for disclosure of PHI or other sensitive information.

For the aim 4 implementation-effectiveness trial, the consent process will be the same as that described in aim 2, save for the five to ten participants at each site who were enrolled in the aim 2 parent trial who will be able to consent virtually through REDCap, described below. The in-person consent visits for the participants who were not in the aim 2 trial, will be coordinated alongside existing clinical care at every opportunity to minimize the need for the participant to make additional trips outside of their home during the COVID pandemic. The participant will be seen only briefly during the consent visit to carry out the informed consent procedures and to provide the participant with the blood pressure cuff and education for home blood pressure monitoring. Verbal consent will be obtained and documented for scheduling and documenting participants' consent visits in participants' Electronic Medical Records at Duke Health. Pre-baseline visit medical history chart review will follow procedures described in aim 2. Missing data from chart abstraction will not be attained until the second *virtual* study visit rather than the in-person consent and baseline visit. The reason for this is to minimize the length of the in-person visit given that this is an intervention to test the effectiveness of virtual implementation of the EXTRA-CVD blood pressure management.

Consenting the former aim 2 participants virtually is preferred over in-person in order to reduce face-to-face contact during COVID-19 and optimize the study team and participant engagement experience through virtual means, which is a key element of aim 4. These

participants have consented in-person in the past for aim 2, built a relationship with study team members, and will already have the blood pressure cuffs that necessitate the in-person consent visit for other participants who were not a part of aim 2. For those aim 2 participants interested in enrolling in aim 4, the study team member will schedule a time to conduct the informed consent process with the participant using the participant's preferred remote method of communication (e.g. phone, videoconference). The study team member will offer to send a hardcopy of the consent document to the participant's mailing address if the participant would like to review the document before the virtual consent meeting. On the scheduled consent date, the participant will be sent a unique link to the full consent document in REDCap. Before the informed consent process begins on the scheduled date, the study team member will confirm the identity of the participant by one of two ways. 1. If the participant has robust Internet access and a computer device, the participant will send a picture of their government issued picture ID to the study team member's UH, Duke, or Metro email or sending the image through short message service (SMS) to a secure study cell phone (registered with the research institution). 2. If the participant can only communicate virtually through telephone without SMS or reliable Internet, the study team member will ask them to provide their full name, date of birth, and medical record number and compare the provided information to their medical record. These participants are already known to our study teams at each site, and have communicated throughout the aim 2 trial with the study nurses and coordinators. We believe this second option is more than sufficient in confirming the identity of participants with whom the study has already gained familiarity. This virtual consent will capture an electronic signature and follow most of the guidelines laid out by the UH IRB in their document titled *Guidelines for Remote Electronic Consent*, save the exception stated above (identity confirmation option 2). The participant will select in REDCap whether or not they would like to be contacted for future research, how their data may be used, and will provide their electronic signature confirming their consent to participate.

Finally, we will also use remote electronic consent procedures identical to the aim 4 intervention adaptation to re-consent Design Team participants to participate in a two part process to determine a stakeholder informed composite measure of intervention dose.

<u>Aim 1 Procedures</u>:

PLHIV (n=60): After signing informed consent, all PLHIV participants will complete the following assessments:

1. A self-reported survey consisting of demographics, HIV and medical history, perceptions of CVD Risk (Appendix A; Health Beliefs for Cardiovascular Disease Scale[75]), and Adherence to Cardiovascular Medications (Appendix B Adherence to Hypertension and Cholesterol Medication Scales);
2. A standardized interview of their family history of CVD; and
3. An in-person interview discussion on perceptions of ASCVD risk and barriers/facilitators of ASCVD preventive care (see below). Medical chart abstraction will be used to determine history of use, adherence to, and tolerance of proven CVD prevention therapies.

<u>Key Informant Interviews (PLHIV).</u> Prior to enrolling across all three sites, we will first pilot test an interview guide (see Appendix C) on 3-5 PLHIV in Cleveland and revise it accordingly. The final guide will be used to direct subsequent interviews consistently across

sites. The in-person interviews will take place at a secure, mutually agreed upon location. Questions will address the participant's perceptions of ASCVD risks associated with HIV, risks associated with HIV medications, and CVD risk reduction measures. Additionally, we will assess barriers to and facilitators of improved ASCVD prevention.

All interviews will take approximately 30 minutes, and audio recordings will later be transcribed verbatim. The interviews will be conducted by Dr. Webel or a trained qualitative researcher at the study site. Dr. Webel has over 10 years of experience designing, implementing, and analyzing data from qualitative research studies.[49,65-68] She has also successfully trained and managed qualitative data collectors and obtained high-quality data.[49,67]

Healthcare team members (n=36): After signing informed consent, all healthcare team member participants will complete the following assessments:

1. A self-reported survey consisting of demographics, training, and general practice patterns
2. An in-person interview discussion on perceptions of ASCVD risk among patients with HIV in general (see below) and barriers

Key Informant Interviews (Healthcare Team members). In a similar fashion to the PLHIV interviews, we will conduct individual in-person interviews to understand care team members' perceptions of their patients' ASCVD risk and how those perceptions influence the care they provide (i.e., decision to screen PLHIV for high cholesterol, knowledge of ASCVD prevention guidelines, when to refer to specialists, how they treat CVD risk factors, and the individual CVD risk reduction counselling they provide PLHIV). We will pilot test a key informant guide and revise it accordingly. The final guide will be used to direct the key informant discussions consistently across sites and ensure we have covered all relevant topics. These ~30 minute interviews will take place in a private room chosen by the staff member, and audio recordings will later be transcribed verbatim. Care team members will also complete a basic demographic form and information about their practice setting.

Outcomes and analysis: Quantitative data (e.g., demographics, medical history, perceptions of CVD risk) will be summarized and used to describe the study samples. After redacting all names and identifying information, verbatim transcriptions of recorded interviews will be entered into Dedoose[76], a secure, website-based analysis program to analyze qualitative data. A quality assurance protocol will be built into data management and analysis; 25% of the transcripts will be check to verify accuracy of the transcriptions and 10% will be double-coded to ensure inter-coder reliability of 80% or greater. Pooled Kappa statistics will be calculated for codes to assess inter-rater agreement.[77]

Under PI-Webel's direction, all responses will be analyzed using standard analytic techniques for qualitative data: identification of themes/domains; coding or classification of participants' responses by these themes performed independently by two team members (who have graduate-level training in qualitative coding); resolution of any coding discrepancies will be done by a third team member.[78] To ensure consistency, a codebook and dictionary will be developed to create universal definitions for each code. The codebook will contain all codes, their definition, and exemplar quotes.

For **ASCVD risk perception**, we will search for (*a priori*) codes that describe how perceptions of ASCVD risk are influenced by HIV and how that perception influences ASCVD

preventative care and behaviors. Significant inductive (emerging) codes will also be identified. Coded items will be grouped together into distinct themes. Group analysis meetings will be held to compare independently-developed codes for similarity and further direction. Once the codebook is developed and verified, all transcripts will be coded. Finally, the analytic team will work from the coded data to merge findings into a final report to aid the EXTRA-CVD intervention adaptation (sub-aim 1.1).[38,65,79] This method of data reduction encompassing a multidisciplinary team-based analysis creates a robust iterative process through which the data are thoroughly discussed and analytical consensus achieved.

For **barriers and facilitators of ASCVD preventive car**e, we will search for *a priori* codes related to ***1) Patient Facilitators***: a) Health Beliefs, b) Health Priorities, c) Social Influence and Support, and d) Care Team Factors; ***2) Patient Barriers***: a) HIV-related factors, b) Health Beliefs, and c) Health/ CVD knowledge; ***3) Care Team Facilitators***: a) Care Team Factors, b) Research/Guidelines, and c): Patient Factors; and 4***) Care Team Barriers***: a) Visit Logistics, b) Knowledge/Comfort, and c) Patient Factors. Significant inductive (emerging) codes will also be identified. Coded items will be grouped together into distinct themes and will be used to prepare a report of findings to aid in the EXTRA-CVD intervention adaptation.

* For all interviews conducted by telephone at the Duke site, the participant will respond to survey questions on risk perception and medication adherence that are read over the phone.

Clinic Variables Checklist:

During the Aim 1 baseline assessment, investigators from each study site will complete a clinic variables checklist (Appendix D) in consultation with the site clinic director. This form will characterize site-specific factors that may influence the results of all three aims of this study. These data will be used to characterize contextual aspects of effect modification by site.

Sub-Aim 1.1 Procedures:

The EXTRA-CVD Human-Centered Design process:

We will use a participatory, iterative design process with two "design teams" (one team from Duke and another team consisting of both study sites in Cleveland combined), representing key stakeholders from each geographical site. For the purposes of this study, the design process will involve three key phases: Brainstorming, Conceptualization, and Creation and two additional meetings to refine the intervention for the acceptability and feasibility before the trial begins – making it a total of 5 sessions for this iterative design process. For both Design Teams, a focus group discussion on the human-centered design experience will be conducted at the end of the final or fifth design team meeting among those who consent to participate.

Aims of the EXTRA-CVD Human-Centered Design Process

This human-centered design process has been adapted specifically for the EXTRA-CVD nursed led intervention as noted in Sub-Aim 1.1 of the study aims, i.e. to adapt the intervention within HIV clinic contexts through an iterative approach of collaborative meetings with key stakeholders. The following aims have been developed for this human-centered design phase:

Aim 1: Refine the nurse-led intervention using the human-centered design approach with key stakeholders input.

Aim 2: Evaluate the ***acceptability*** of the nurse-led intervention through semi-structured interviews or focus group discussions with PLHIV and HIV community advisory boards

Aim 3: Conduct a pilot study of the intervention and assess the ***feasibility*** as well as perceptions of the intervention with those involved in the pilot study through semi-structured interviews and focus group discussions.

Aim 4: Assess the reflections of the design team members on the human-centered design process though survey and focus group discussions.

Initial Meetings: This intervention adaptation aim or human-centered design process will involve three initial phases: brainstorming, conceptualization, and creation:

During brainstorming (Meeting #1), the design team will review the mixed-methods ***de-identified*** data obtained during the baseline assessment on perceptions of ASCVD risk and barriers to and facilitators of ASCVD preventive care. The team will discuss these findings and brainstorm ideas to refine the EXTRA-CVD intervention in response to these data. Importantly, the design team will also review all data elements to be collected during the intervention in order to conduct the comprehensive process evaluation (aim 3). Possible targets for intervention adaptation include: (a) adjusting when, where, and to whom the EMR alerts appear; (b) adapting the treatment algorithms to overcome barriers and maximize the facilitators; (c) targeting the staff training to include relevant aspects of perceived risk into the care coordination and adherence support; (d) developing and tailoring staff training to facilitate acceptance, uptake, and effectiveness; and (e) helping us to quickly identify and troubleshoot any problems with the implementation of the intervention.

- At Design Team Meeting #1, design team participants will be recruited to participate in a research study that evaluates the design process. The research assessments will be a brief survey completed at this initial meeting and a focus group discussion during meeting #5 described below.

In the conceptualization phase (Meeting #2), the team will evaluate advantages and disadvantages of ideas generated during the brainstorming, and will develop concrete changes to the intervention. For example, if team decides to include the name of the provider (PCP or HIV specialist) responsible for BP and cholesterol management on the EMR dashboard, the IT representatives will discuss its feasibility followed by a discussion of pros and cons. Final decisions will be made by majority vote of the design team; however, the study PIs may decide to veto modifications deemed to be counterproductive or an inefficient use of resources.

The creation phase (Meeting #3) will involve the creation of refined treatment protocols, manuals of procedures, and educational materials.

After the first three phases, there will be two Iteration meetings that will take place during the acceptability and feasibility phases.

Acceptability Testing: Acceptability testing[80] will be accomplished in 2 settings: (1) key informant interviews or focus group discussions with PLHIV pilot participants (2-3 per site, up to 9 total) and (2) HIV Community Advisory Board (CAB) meetings at UH and Duke.

Key informant interviews or focus group discussions with PLHIV pilot participants will take place at local clinic sites and will be conducted in a similar fashion as described in Aim 1. Each interview or focus group discussion will last for roughly 60 minutes and include participant's

perceptions and experience navigating the HIV care system as a user or a provider. The interview/discussion guide (Appendix E) will include the following elements: thoughts on HIV and CVD care and management; facilitators and barriers of implementing the nurse-led intervention within the clinic setting; and logistical factors (i.e. resources or tools at that clinic) that may or may not facilitate the integration of the intervention. Data will be analyzed in Dedoose in a similar fashion to Aim 1 described above.

Study investigators will present the intervention to a CAB meeting after the third design team meeting to further assess acceptability. The CAB meetings are held monthly at UH and at Duke; there is no CAB at MetroHealth but some MetroHealth patients attend UH CAB meetings. A research assistant will attend and take notes on the content themes and specific suggestions discussed, but there will be no personal identifying data collected.

* ***Design Team Meeting #4***: After the acceptability testing is completed, the design team will meet again (meeting #4) to discuss initial feedback on acceptability from pilot participants and CAB and to refine the intervention as needed.

Pilot Trial: A 6 week pilot of the intervention will be conducted among 2-3 participants per site (up to 9 total). The pilot trial procedures are summarized graphically as follows:

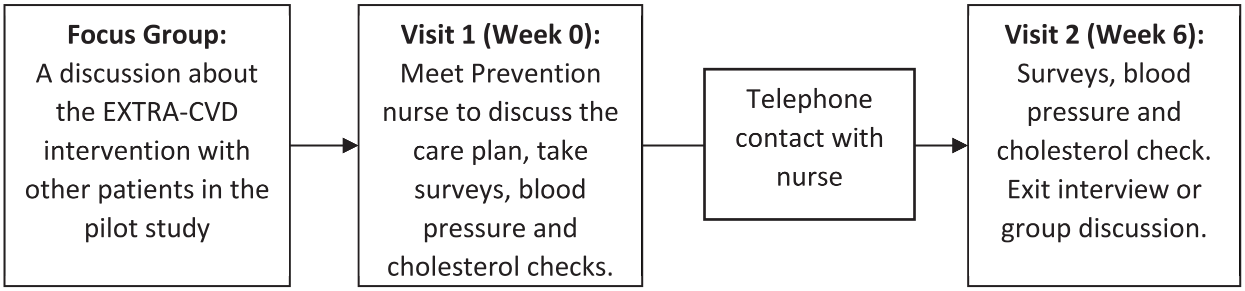

At each of the two pilot study visits (week 0 and week 6), the PLHIV pilot participant will complete all study assessments as described in Aim 2 below. In addition, at the time of the week 6 follow-up visit, the participants will participate in an exit interview or focus group discussion about the feasibility of the intervention as described below. All pilot participants will be able to keep the BP monitor after completion of the study.

The time-frame of the pilot is compressed compared to the overall trial described in Aim 2 below; however, there will be ample opportunity over 6 weeks to make sure that participants are able to use the home blood pressure monitors appropriately, that the telephone interaction with the prevention nurse is adequate, and that the medication treatment algorithms work smoothly. At a minimum, the prevention nurse will call once at the half-way point (~2-3 weeks) to "check-in" about home blood pressure values and medication issues.

Feasibility Testing: After the pilot study is completed, all PLHIV pilot participants will participate in either a focus group discussion or semi-structured interview depending on patient preference and logistical considerations (scheduling, availability of research staff, etc…). The purpose of these interviews/discussions will be to explore the feasibility of the intervention. The interview/discussion guide for PLHIV pilot participants can be found in Appendix F. The discussion will last 30-60 minutes and will focus on participants' perceptions of the nurse-led

intervention and the facilitators and barriers of the intervention that may impact the sustainability of the intervention.

Additionally, after the pilot is completed, up to 18 healthcare workers (6 per site) who have in some way been exposed to the pilot intervention will be recruited to participate in semi-structured interviews for feasibility testing. Examples of such health workers may include: (a) a primary HIV doctor who is asked by the prevention nurse to add a new blood pressure medication, (b) an HIV nurse who interacts with the prevention nurse to coordinate care between HIV provider and PCP over a cholesterol management issue. Each interview will last 30-60 minutes and will focus on the following elements: general thoughts on HIV and CVD care and management; perceptions of the nurse-led intervention; and the facilitators and barriers of the intervention that may impact the sustainability of the intervention. The interview guide for health worker feasibility testing can be found in Appendix G.

All qualitative data from this feasibility testing will be analyzed in a similar fashion as described in Aim 1.

* ***Design Team Meeting #5***: After the feasibility testing is completed, the design team will meet for a final time (meeting #5) to discuss feasibility testing results and refine the intervention as needed. At this meeting, the design team members who have given consent to participate in a focus group discussion will reflect on the design process. The discussion guide can be found in Appendix H. This discussion will be recorded and the qualitative data will be analyzed in a similar fashion as described above.

****** After the intervention adaptation design team process is fully completed, any changes to the protocol suggested by the design team will be submitted to the IRB for approval prior to starting the Aim 2 clinical trial. The August 2019 modification contains these adaptations to aim 2 procedures as described in the Aim 2 procedures section below.***

Additionally, in Q4 of 2022, the Design Teams from Cleveland and Duke will be invited to participate in a 2-part process to formulate a composite measure of intervention dose. The protocol and manual of procedures for this study was developed in detail by Dr. Angela Aifah (NYU) who will lead this part of the study with funding from her Diversity Supplement application. The full protocol is available in Appendix T. In brief, participants will participate in the following procedures:

- **Part 1: Group concept mapping**: Each participant will be sent a unique link from study investigators to create an on online account at for the GCM portal (https://groupwisdom.com/groupconceptmapping). Through this application, the participants will complete the following steps of answering questions in response to various prompts. The total duration of steps 1-4 + 6 is expected to take a total of ~1.5-2 hours and does not have to be completed all at one time.
    - Step 1 - Preparation
    - Step 2 - Generation
    - Step 3 - Structuring

- Step 4 - Representation
- Step 5 - (see below)
- Step 6 – Utilization

- **Group concept mapping Step 5 – Interpretation**: This step will be completed during a 1-hour Zoom call so that all participants will be able to work together to interpret the results of steps 1-4.

The 6 steps of group concept mapping are shown in the Figure below. The outcome of the group concept mapping phase will be a shortlist of individual variables that participants believe best represent the dose of the intervention delivered by the EXTRA-CVD and AAIM-HIGH interventions.

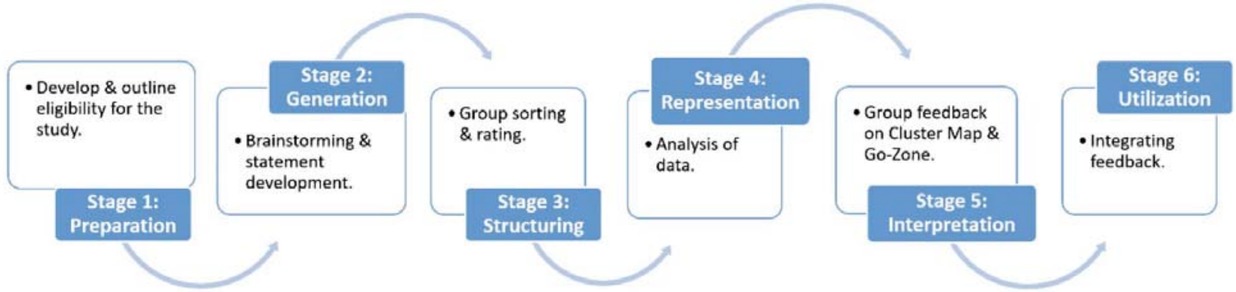

- <u>**Part 2:**</u> **Delphi Process**: This part of the study will further refine the list of variables generated during the group concept mapping phase to create a final composite measure of dose that incorporates different weights given to variables based on what stakeholders believe to be the most important aspects of the intervention. There will be 3 rounds of questionnaires to complete online and the total time required will be ~30-45 minutes.

The final composite measure of dose that results from this process will be used in analyses as a potential mediator of EXTRA-CVD outcomes, including both primary and secondary clinical outcomes (BP & cholesterol) but also implementation outcomes and other outcomes of interest (i.e. time to disengagement for those who are lost to follow-up).

<u>**Aim 2 Procedures:**</u>

We will conduct a randomized controlled trial of the EXTRA-CVD intervention vs. education control among PLHIV on suppressive ART who have both hypertension and hypercholesterolemia. Control participants will receive general prevention education. The intervention—contextually adapted during sub-aim 1.1—will consist of prevention education plus 4 additional evidence-based components: (1) **nurse-led care coordination,** (2) **EMR alerts and decision support,** (3) **home BP monitoring**, and (4) **nurse-managed medication protocols and adherence support**. The primary outcome is change in systolic BP and the secondary outcome will be change in non-HDL cholesterol. Separately for hypertension and hypercholesterolemia, we will then examine changes in the three extended treatment cascade categories [(1) % appropriately diagnosed, (2) % appropriately managed, and (3) % at treatment goal]. We chose BP as the primary outcome because the EXTRA-CVD intervention components

were designed primarily to address BP management, with cholesterol management being an important but secondary consideration. For the purposes of determining cascade level #1, we will use the following table to search for diagnosis of high cholesterol or blood pressure in the medical record.

Table: Diagnosis terms for high cholesterol and high blood pressure for the purposes of determining cascade category from chart review.

High Cholesterol	High Blood Pressure
Hyperlipidemia	Hypertension
Dyslipidemia	Essential Hypertension
Hypertriglyceridemia	Secondary Hypertension
Hypercholesterolemia	Hypertensive end-organ disease
Elevated LDL	High blood pressure
Elevated Triglycerides	
Elevated Cholesterol	
Familial Hypercholesterolemia	
**DO NOT include the following:**	**DO NOT include the following:**
Low HDL	Pulmonary hypertension
	Intracranial hypertension
	Venous hypertension
	Pre-eclampsia or Maternal Hypertension
	Portal hypertension
	Ocular hypertension

Randomization: Prior to study start, Dr. Smith will develop a 1:1 blocked randomization scheme, stratified by site. This randomization scheme will be carried out using the randomization module in RedCap. Participants will be randomized by RedCap at the time of their baseline visit to avoid randomizing participants who have not yet completed consent.

Education control group: Participants assigned to the education group will receive usual care enhanced with general prevention education delivered by the prevention nurse. This active comparator is appropriate because participants have multiple risk factors for ASCVD, and advisory committees for prior studies have recommended this for similar study populations. The prevention educational modules will be delivered at 4 in-person visits (enrollment, 4, 8, and 12 months), and will consist of evidence-based material on diet, exercise, smoking, sexually transmitted infections, and cancer prevention. As recommended by the design teams, control participants will have access to a wide range of educational materials about healthy living, but will not receive additional counseling from the prevention nurse outside of the 4 in-person visits.

All participants in both intervention and education control arms will complete the following study assessments: 1) in-office BP obtained by a trained research assistant blinded to study group using a standardized protocol[12] (0, 4, 8, 12 months); 2) lipid profile (0, 4, 8, 12 months); 3) perceived ASCVD risk survey[56] (0, 12 months); 4) provider trust and communication survey (0, 12 months). Study visits will be calculated from the date of the baseline enrollment visit in months. Per protocol, study visits must be completed +/- 14 days from the calculated visit date.

Visits done between 14 and 28 days after the calculated visit date will be considered a protocol deviation. Visits outside of the +28 day window will not be scheduled and that visit will be considered missing for that patient. The fasting lipid panel blood draw will be done at the time of study visit or within a +/- 10 day window. To limit potential bias, participants, their healthcare providers, and the prevention nurse delivering the education modules will be blinded to the in-office BP outcome measure unless a safety threshold is exceeded (systolic BP <90 or >180mmHg). BP may be re-checked separately as part of routine care.

Control participants will be encouraged to maintain participation in the study in order to minimize differential loss-to-follow-up between intervention and control group that would jeopardize the validity of study findings. As recommended by the design teams, we will conduct semi-annual participant engagement parties (in January and July) that will consist of a meal, healthy lifestyle education and games. We will report enrollment and other study related news to participants. All participants—both intervention and control—will be invited to these events. As an additional incentive, control participants will receive a BP monitor and instructions about its use after completion of the final 12-month trial visit.

EXTRA-CVD intervention: Participants randomized to the intervention will meet with the site prevention nurse on the day of enrollment. With nurse assistance, the participant will complete an initial ASCVD risk assessment using the ACC/AHA risk calculator and a risk visualization tool available at https://statindecisionaid.mayoclinic.org/. This exercise will help establish rapport between the participant and the nurse, and will help identify potential targets for intervention. The nurse will conduct a baseline medication assessment, including participant's knowledge of the purpose and side effects of each BP or cholesterol medication and current or potential adherence strategies.

Frequency of contact: All subjects will complete the same assessments at the same time-points (in-office BPs, lipid profile, perceived ASCVD risk survey, and network analysis survey). The prevention nurse will then contact the intervention subjects at up to 2-week intervals as necessary to carry out the multi-component intervention. Frequency of contact will be determined according to protocol triggers that may be patient or prevention nurse initiated (e.g. home BP values are high, checking for side effects after starting a statin). At a minimum, the prevention nurse will have face-to-face meetings at 0, 4, 8, and 12 months, as well as a mid-period telephone call between in-person visits to "check-in" about home BP values and medication adherence. An initial 2-week follow-up call will ensure proper use of the home BP monitor and to address any other questions. Two example scenarios of relatively lower intensity and higher intensity intervention are shown in **Figure 3** with the education control group as a reference. As demonstrated by the figure, a strict attention control group is not appropriate since the dose of attention will vary according to the needs of the participant.

As with control patients above, study visits must be completed +/- 14 days from the calculated visit date. Visits done between 14 and 28 days after the calculated visit date will be considered a protocol deviation. Visits outside of the +28 day window will not be scheduled and that visit will be considered missing for that patient. The fasting lipid panel blood draw will be done at the time of study visit or within a +/- 10 day window. Study calls will be completed +/- 14 days of the scheduled due date. In the event that the study nurse has made no less than three

attempts to schedule a participant's follow-up visit, a letter (Appendix R) will be sent to the participant.

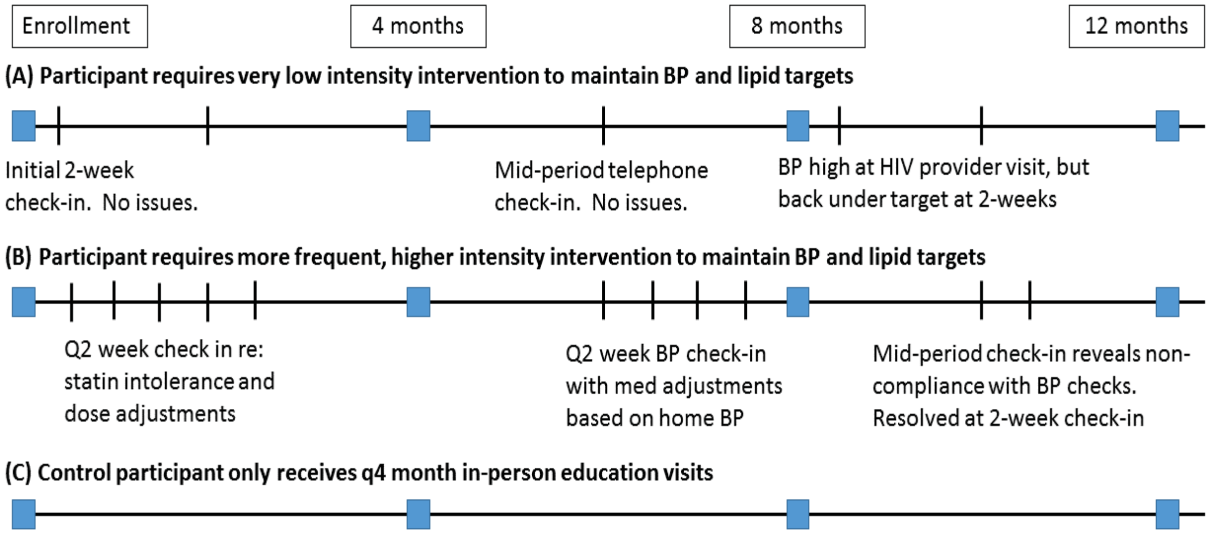

Figure 3: Example scenarios of intervention participant contact frequency. (A) Participant with lower intensity requirements; (B) Participant with higher intensity requirement; (C) Control participant. Squares represent in-person visits and lines are telephone contact.

EXTRA-CVD Intervention components:

1. Care coordination. Beginning with initial enrollment, the prevention nurse will coordinate blood pressure and cholesterol management for all participants in the intervention arm. Care coordination will consist of tailored discussions with the participant and his/her providers about which provider will take primary responsibility for BP and cholesterol management. Considerations of patient-provider trust and provider comfort level or experience will inform this decision. The prevention nurse will direct subsequent management decisions to the designated provider but will facilitate communication by notifying the non-designated of any changes to medications.

At the baseline visit, the prevention nurse will map the participant's care team and communication preferences, to ensure that all providers in the patient's care team are accounted for. After the initial visit, the nurse will contact members of the participant's care team through normal clinical communication channels which are standard of care for that clinic site (e.g. EMR messages, email, phone) to inform them that the participant has been enrolled in the study. The prevention nurse may use this opportunity to discuss any potential modification to suggested blood pressure and cholesterol targets.

In order to better incorporate the prevention nurse into clinic workflow, a number of recommendations were made by the design teams. These include having nurse meet & greet and regular staff updates on the study to ensure that communication preferences are respected.

Component 1—Relevant process evaluation data for aim 3: (1) number and duration of telephone calls and emails to providers or providers' staff; (2) number and nature of communication notes

in EMR; (3) prevention nurse trust & communication ties with providers; (4) time spent coordinating care.

2. Nurse-managed medication protocols and adherence support. Participants with BP or non-HDL above goal will receive tailored medication management and adherence support. Algorithm-based care to reduce practice variation and clinical inertia has long been recommended to assure that patients are not "stuck" at sub-therapeutic doses of medications[82]. A meta-analysis of nurse and pharmacist-led cholesterol medication adherence interventions showed substantial improvements in adherence and 15-20mg/dL reductions in total cholesterol[42]. By using algorithms and clear decision rules to guide medication titration, the prevention nurse will make recommendations to providers to improve care by reducing clinical inertia, reducing variation, and allowing non-physician staff members to assist in care. A clear and complete algorithm will also help simplify the medical regimen and emphasize medications that are affordable, effective, and have low side-effect profiles.

At each visit (in-person or telephone) where recent home BP values (average weekly BP based on a minimum of three values) exceed 130/90mmHg (in line with 2017 ACC/AHA hypertension guidelines), the prevention nurse will review the medication list with the patient, including any recent medication regimen changes and potential side-effects of each medication[83]. The nurse will provide counseling in several areas, including ways to enhance medication adherence and prevent or ameliorate side effects. For example, patients may be given a personalized medication schedule that shows when they should take their medications.

The prevention nurse will use the algorithms described below to decide on appropriate recommendations for medication changes and will approach the designated responsible provider (HIV, PCP, or non-HIV specialist) for prescriptions and lab orders. *The responsible provider will ultimately decide on final management decisions and may request to have the participant be taken OFF management protocols as clinically indicated (e.g. recent ASCVD events or advanced CKD), in which case the participant would continue all other components of the EXTRA-CVD intervention*.

Blood pressure. We will use an evidence-based blood pressure treatment algorithm (**Figure 4**, Tables and Figures uploaded separately) adapted from Kaiser Permanente and used in our prior studies.[84,85] Once-daily medication and combination therapy will be recommended when possible. A follow-up a basic chemistry panel will be ordered when adding ACE/ARB, thiazide diuretic, or potassium-sparing diuretic. Medication up-titrations will be recommended at intervals of 2-4 weeks until control is achieved. Measures not shown in the figure will include but will not be limited to: (1) adding agents such as hydralazine, terazosin, clonidine; (2) considerations for comorbid kidney disease or prior ASCVD event; (3) avoiding combination use of heart rate slowing drugs.

Figure 4: **Blood pressure treatment algorithm**

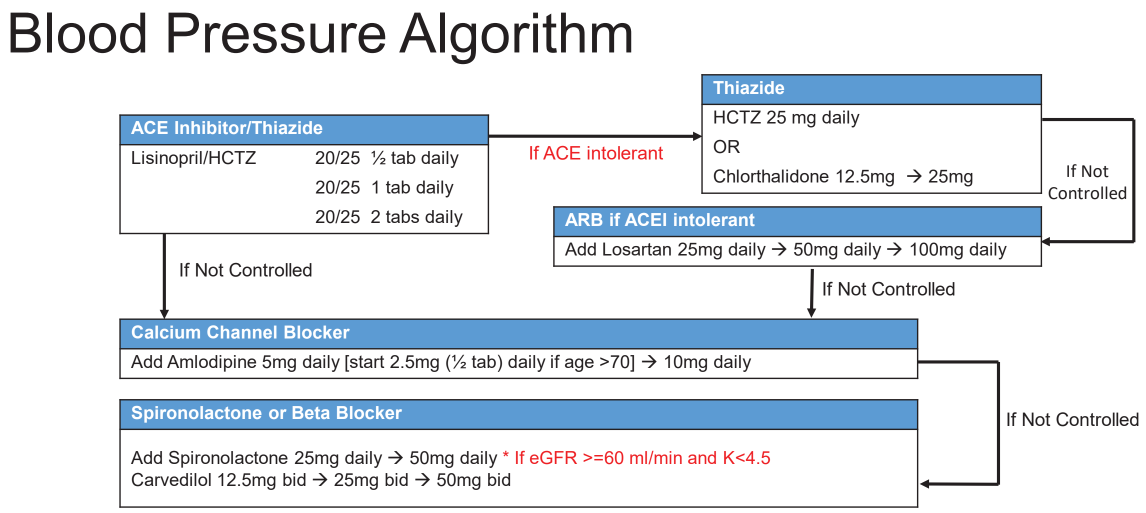

Other considerations

- Consider medication non-adherence
- Consider interfering agents (e.g. NSAIDS, excess alcohol)
- Consider white coat effect.
- Consider discontinuing Lisinopril/HCTZ and changing to chlorthalidone 25mg plus Lisinopril 40mg daily. Consider additional agents (hydralazine, terazosin, minoxidil)
- Avoid using clonidine, verapamil, or diltiazem together with a beta blocker. These heart rate-slowing drug combinations may cause symptomatic bradycardia over time
- Consider consultation with a hypertension specialist.

Figure 5A: Cholesterol treatment algorithm (part 1)

Cholesterol Algorithm

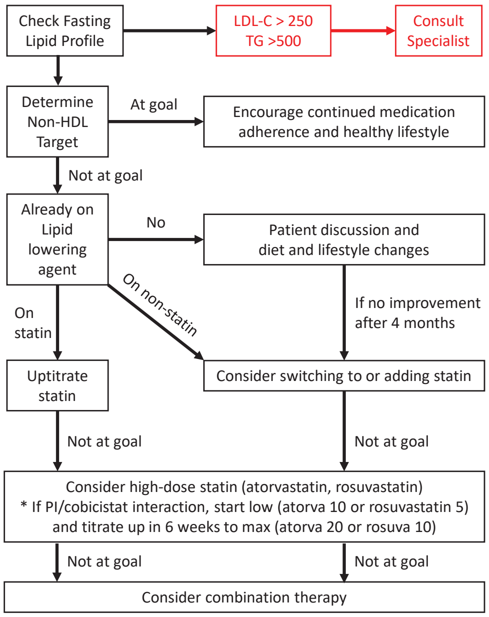

Risk Category	Criteria	Non-HDL Goal
Low-Moderate	• Most EXTRA-CVD patients	<130 mg/dl
High	• Known ASCVD<br>• Diabetes + ≥2 major risk factors *	<100 mg/dl
Very High	• Multiple Major ASCVD events<br>• 1 major ASCVD event + risk factors* (see below)	<100 mg/dl

Major ASCVD events
1. Recent acute coronary syndrome (<12 months)<br>2. History of MI or stroke (other than recent ACS above)<br>3. Symptomatic PAD (claudication with ABI <0.85, prior lower-extremity revascularization or amputation)

Risk Factors for Future ASCVD events
1. Age >65 years<br>2. Heterozygous Familial Hypercholesterolemia<br>3. History of prior CABG or PCI outside of the major events<br>4. Diabetes<br>5. CKD (eGFR 15-60 mL/min)<br>6. Current cigarette smoking<br>7. Congestive Heart Failure<br>8. Persistently elevated LDL >100mg/dL despite maximally tolerated statin therapy and ezetimibe

<u>Figure 5B:</u> Cholesterol treatment algorithm (part 2)

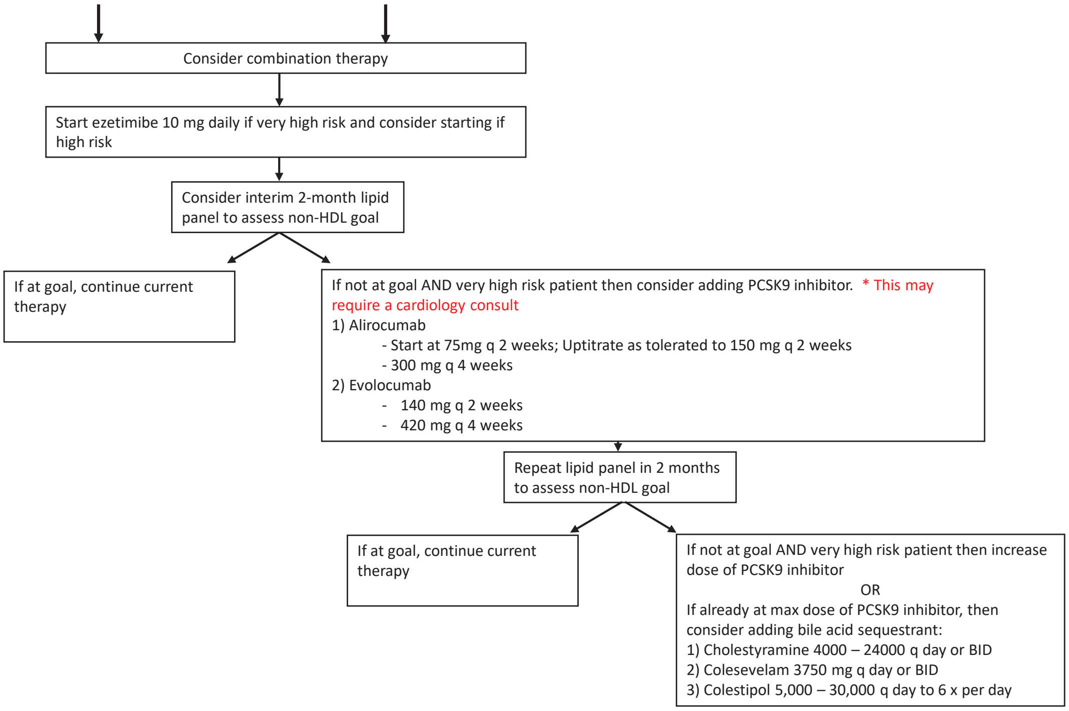

Figure 6: **Protocol for management of statin associated muscle symptoms**

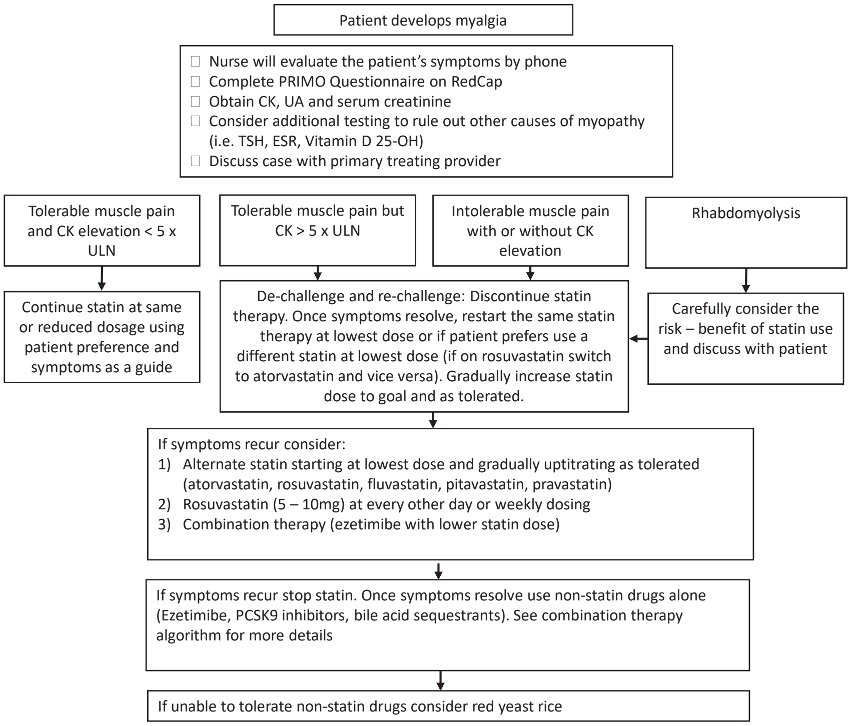

Cholesterol. We will use an algorithm (**Figure 5A-B**) adapted from National Lipid Association (NLA) guidelines for HIV-infected patients[25] and the 2018 ACC/AHA Guideline for the Management of Blood Cholesterol[81]. As a first step, the prevention nurse will determine the non-HDL target for each individual participant (Table 1; p. 7). For most participants in the trial, the target non-HDL will be <130mg/dL; however, high risk patients (such as those with history of prior ASCVD event) will have a more aggressive goal (<100mg/dL). As recommended by the guidelines, our algorithm will address drug-drug interactions with ART, including the safe use of higher dose statins (rosuvastatin and atorvastatin) if needed, when drug interactions are present. Lipid profiles (total cholesterol, triglycerides, HDL, LDL) will be checked at every in-person study visit. The nurse will have access to all cholesterol fractions, but the algorithm will focus on non-HDL as the primary target. When a new cholesterol medication is prescribed, the prevention nurse will call 2 weeks after initiation to discuss adherence and any possible side-effects. The nurse will use an evidence-based approach to evaluation and management of muscle symptoms and other intolerances of statins as recommended by NLA guidelines (**Figure 6**).[82,83] This approach will include evaluation for other causes, drug-drug interactions, checking creatinine kinase levels, trial off statin, retrial of different statin, non-daily dosing of longer acting statin (i.e. rosuvastatin), and/or referral to a specialist.

Component 2—Relevant process evaluation data for aim 3: (1) frequency of BP and cholesterol algorithm use; (2) number of telephone contacts and total duration of time required to bring an elevated BP or cholesterol level under control; (3) Frequency of statin intolerance and proportion of intolerance cases ending in complete cessation of any statin; (4) Number of referrals to BP or cholesterol specialists.

3. Home BP monitoring. Our justification for using home BP monitoring in the EXTRA-CVD intervention is that home BP measurements are reproducible with standard deviations of less than 3.1 mmHg for both systolic and diastolic measurements[84]. In addition, home BP monitors are accurate and comparable to ambulatory BP monitors[85], a 'gold standard' of BP measurements. Home measurements have greater predictive power for mortality as compared to office-based measurements.[86] All intervention participants will receive a home BP monitor and will be trained according to a developed protocol[14] and documentation of proper usage will be recorded. Participants will use the BP monitors every day. Prior to each telephone or in-person follow-up visit, we will request BP values for the past two weeks using a standardized data collection form. Participants with poor BP control will receive nurse calls every 2 weeks, with management changes made as described in component #2 above. All intervention participants will be able to keep the BP monitor after completion of the study. Control participants will receive a BP monitor and instructions about its use after completion of the final 12-month trial visit.

Component 3—Relevant process evaluation data for aim 3: (1) Frequency of home BP checks (average checks/week and proportion of weeks with ≥ 2 checks); (2) Number and nature of medication changes in response to home BP data; (3) Barriers to home BP use; (4) Knowledge of proper home BP use.

4. EMR support tools. Evidence-based EMR tools that do not result in provider fatigue or information overload effectively improve patient outcomes[87,88]. Our three sites use two of the most widely available EMR systems (Epic at MetroHealth/Duke and Allscripts at UH). At the UH Special Immunology Unit, there is also a parallel electronic database that may be used for clinical purposes and is amenable to programmable tools. Beginning in year 1, we will work locally with EMR (IT) support to develop these tools to assist the prevention nurse during the intervention phase:

- An extended treatment cascade graphic for the prevention-nurse specialist which will appear on his/her "dashboard" or as a recurring pdf report. During the intervention phase, the nurse will have regular access to this graphic and will receive names of specific patients who have fallen out of each cascade category.
- Tool to chart home blood pressures in EMR.
- Epic SmartSets that pipe in recent lab and BP values and give providers options for medication prescriptions based on the treatment algorithms.

<u>These new tools will only be available to the prevention nurse during the intervention phase</u>, but will then be made available to all providers after the intervention is completed. Currently, all three sites have automatically calculated 10-year predicated ASCVD risk available to all providers for all patients.

<u>Component 4—Relevant process evaluation data for aim 3:</u> (1) number of times each tool is accessed

<u>Protocol Changes Resulting from Novel Coronavirus Outbreak (COVID-19)</u>

We will not enroll new participants during the period of spread of the novel coronavirus in the United States. No new participants will be enrolled until deemed safe by the affiliated research institutions and the HIV clinic directors where the EXTRA-CVD intervention is taking place. Study procedures will revert to the original procedures described herein on pages 25-35 once the mandates are lifted. During this time, recruitment may continue as described above according to the discretion of each study site. Potentially eligible subjects will be placed on a waiting list to be enrolled once mandates are lifted.

In order to simultaneously reduce transmission of the virus while maintaining the management of participant's cardiovascular care to the best extent possible, pertinent study activities will be conducted remotely with currently enrolled participants who are willing and able to access HIPAA-compliant virtual platforms (e.g. secure telephone and virtual videoconferencing). These relevant research activities include follow-up visits with control and intervention groups and periodic check-ins with intervention participants, both described above in the study procedures for aim 2. Participants will be compensated for their participation at the same amount specified in the original consent through site-specific means such as check or prepaid debit cards by mail or direct deposit.

Participants who cannot or prefer not to participate in remote study activities during this time will have 4- or 8- month follow-up visits that are designated as missing. These participants will remain enrolled and will be contacted once the COVID-19 public health mandates are lifted and will be invited to re-engage in their next calculated visit as described in Figure 3 on page 27.

All enrolled participants at each site will be notified by mail of these protocol changes enacted during the period of public health mandates.

Once public health mandates for the COVID-19 have been lifted, study sites will re-evaluate the safety of conducting in-person visits and will adapt original and new procedures to guidelines of their respective institutions, the NHLBI, CDC, WHO, and state and national governments. Study procedures for in-person visits will be carried out to capture all original process and outcome data elements.

Follow-Up Visits with Control and Intervention Groups

Participants currently enrolled at the three study sites may be due for 4- or 8- month follow-up visits during the COVID-19 social distancing period.

For both intervention and control groups, the remote follow-up visits will be focused on collecting self-reported survey data, COVID-19-specific questionnaires and symptom screening, and engaging in cardiovascular health education with the study nurse. The intervention group will also engage in blood pressure and cholesterol care management with the study nurse and be asked about medication, blood pressure, cholesterol and clinical care coordination through standard tools developed in REDCap.

The remote visit format will prioritize a subset of self-administered surveys that the study teams across the sites have collectively deemed most essential. Additionally, the procedures have expanded to include a set of COVID-19-specific surveys. Refer to the table below for a list of these questionnaires. Nurses will inform each participant before the administration of the COVID surveys that if they feel uncomfortable completing any of the specific instruments, to let the interviewer know and they will skip to the next question or section.

For those participants with access to the Internet and a computer (including a smartphone), they will have the option to receive a REDCap survey link via email to complete before their remote visit with the nurse. For those who do not have access to the Internet or who do not complete the self-administered surveys before the remote visit, the nurse will attempt to collect the information and enter the data into REDCap. All other aforementioned data will be collected by the nurse in conversation with the participant and entered into REDCap by the study team.

Several study team members at each site estimated the time expected to complete the essential surveys and care coordination components. From these aggregated responses, we expect the remote follow-up visits to take approximately 75 minutes to complete.

A study protocol BP and blood draw will be attempted in conjunction with any provider-scheduled clinical encounter deemed essential for clinical care within a +/- 21 day time window. If this is not possible, these data will be missing. The window for all study procedures (visits, surveys, outcome BP/cholesterol) will be defined as +/- 21 days from the "target visit date". The target visit date is calculated as 4-, 8-, or 12-months from the baseline visit.

**COVID-19 Survey Battery**	
**Instrument**	**Description**
COVID Chaos Instrument	• This survey will ask participants if they have tested positive, been in contact with a positive case and/or had any COVID-19 symptoms since March 1, 2020.

	• Participants with current symptoms will be referred to the local hospital or department of health hotlines.<br>• Remaining items cover the following (timeframe: during COVID):<br>o Household makeup<br>o Activities affecting physical and emotional health<br>o Coping strategies
Loneliness Scale	A 3-item Likert scale (hardly ever, some of the time, often):<br>1. How often do you feel that you lack companionship?<br>2. How often do you feel left out?<br>3. How often do you feel isolated from others?
HIV Self-Management Scale, Short Form	A 12-item Likert scale asking the participant to rate how confident they are in doing things related to HIV (all, some, none of the time):<br>1. Exercising<br>2. Physical activity<br>3. Spiritual/religious practices<br>4. Changing aspects of health<br>5. Achieving health goals<br>6. Modifying diet<br>7. Balancing family priorities with HIV management<br>8. Personal time of enjoyment<br>9. Job activities helping with health<br>10. Educating others on HIV<br>11. Positive stress relief<br>12. Managing HIV symptoms and medication side effects

COVID-19 Symptom Screening and Referral Procedures

The COVID symptoms questionnaire asks the participant if they have experienced any COVID-19-related symptoms (*Cough, Shortness of breath, Loss of smell or taste, Runny nose, Sore throat, Abdominal pain, Feeling poorly, Chills, Vomiting, Severe headache, Muscle aches*) since March 1, 2020. If the participant answers yes to any symptom(s), the nurse will inquire about symptom duration. Participants with symptoms deemed concerning to the research clinical team will be referred by the nurse to the local COVID-19 hotline. The participant will also be connected to any other needed services (i.e. mental health counseling through the HIV clinic, food deliveries through local food banks, unemployment claims through state governments, etc).

Periodic Check-Ins with Intervention Group

The interval check-in phone calls with intervention group participants will proceed as described in the standard procedures for aim 2, described on page 26-27. The frequency of the intervals are driven by protocol triggers already conducted via telephone remotely.

Semi-Structured Interviews to Assess the Impact of COVID-19 on Cardiovascular Health

The EXTRA-CVD study will integrate qualitative analysis through semi-structure interviews with 36 study participants who have already enrolled into the study as of April 21, 2020. Twelve participants at each site (6 intervention and 6 control) will be interviewed.

Participants will be recruited by the study nurse at each site. The study nurse will ask participants during their follow-up visits if they are interested in being interviewed. Those who affirm their interest will be contacted by a qualitative researcher at Case Western Reserve University. The

CWRU qualitative researchers will conduct the interviews for all sites via telephone. The interviewer will perform verbal informed consent using an IRB-approved consent script over the phone, and the participant will be given ample time to ask questions and, if preferred, review the consent script through electronic mail. Participants must verbally give their consent before the interview begins. Those who consent will be interviewed during that call, and this process will proceed until each site has interviewed 12 participants.

Names, email address and phone numbers for all study participants who agree to be contacted from all 3 sites (UH, Duke and MetroHealth) will be entered by site research staff in REDCap. The qualitative researcher at Case will use these contact lists to contact patients and track when the interview was performed

All interviews will be recorded and transcribed, and verbal consent will be captured in a consent record as well as in the transcripts. All de-identified data will be stored in a secure location on Box and only the qualitative researchers will have access.

Transcription and coding of the interviews will follow the same analysis procedures as laid out in Aim 1. See appendix C, *COVID Aim: Key Informant Interview Guide for PLWH* to review the interview script.

Participants in this qualitative sub-study will receive $25 compensation for participating, which will be distributed to participants in the same manner as other study payments according to local site regulations.

<u>Aim 3 Procedures:</u>

This process evaluation of the EXTRA-CVD trial is based on the framework proposed by Saunders et al[72]**.** We will collect information about intervention fidelity, dose delivered, dose received (exposure), dose received (satisfaction), reach, and recruitment.

RedCap instruments will be used to capture the quantitative measures described in each of 4 component sections above. Additional redcap instruments will be used to collect scripted telephone encounter data, particularly from (a) the initial 2-week check-in and (b) the mid-period check-in. The purpose of scripting the calls is to standardize the intervention as much as possible; however, there will be some counseling and other types of follow-up calls (e.g. counseling, arranging appointments, etc…) that cannot be completely scripted.

To additionally monitor intervention fidelity, a study team member who is not the coordinator or prevention nurse will observe a study visit with a patient. He/she will rate the Prevention Nurse and study staff/coordinator in individual domains using checklists (Appendix L). At least one participant encounter will be observed per quarter and the results will be recorded in RedCap; however, additional encounters may be observed and recorded in RedCap using the repeated measures function.

For aim 4 process evaluation, a study team member will virtually observe a visit between the prevention nurse and patient at each site at least once per quarter using checklists (Appendix Q). If the observer is unable to synchronously observe, the prevention nurse will obtain and use a script to request and record verbal consent from the participant to record the entire visit either using a tape recorder if the visit is conducted by phone or the Zoom recording feature if the visit is conducted by Zoom. If the study team member who obtains informed consent from

participants for aim 4 is not the prevention nurse, then another study team member (either the prevention nurse or another study team member) will similarly observe the person obtaining consent.

Evaluating Study Nurse Motivational Interviewing Skills over Time

The process evaluation includes measuring the motivational interviewing (MI) skills of study nurses at each site over time. Each site has received the same MI training and coaching to learn skills that have been proven to build rapport with participants and enhance participant motivations to change health-related behaviors.
We will utilize the Behavior Change Counseling Index (BECCI) (appendix M) instrument to measure the practitioner skills involved in motivational interviewing. The BECCI is an 11-item instrument that utilizes a Likert scale to measure practitioner behaviors that are core elements of fostering participant behavior change talk" in MI. It is a brief instrument that is simple to implement even for untrained staff, and has been tested as a valid and reliable measure in showing changes in practitioner core MI skills over time. Study coordinators, PI Longenecker, and co-investigators at MetroHealth and Duke Health have been trained to complete the BECCI through direct observation or upon listening to a recorded segment of a study visit. They have also been trained as MI supervisors to provide feedback and coaching to the study nurses based on the observation/recording and completion of the BECCI instrument.

For aim 2, starting July 2020, **each site**'s study nurse will **record a ten-minute segment** of **two study visits** with a participant during **each study quarter**. The study nurse will perform verbal informed consent using an IRB-approved consent script, and the participant will be given ample time to ask questions. Participants must verbally give their consent before the nurse continues the recording of the study visit procedures, and this consent will be included on the audio recording. Recordings will be captured using a digital recording device that will be kept secure in a locked cabinet/desk in a locked room. Recordings will be immediately uploaded to a password protected HIPPA-compliant location at the local site (i.e. s: drive at UH site). The study coordinator or another trained study member will complete the BECCI after listening to the recording and will enter the scores in the REDCap process evaluation arm. No participant PHI will be entered into the REDCap BECCI instrument. A study coordinator will then arrange a time with the study nurse to complete the coaching session. During this session, a study coordinator will provide to the nurse the BECCI results as well as up to two MI learning resources, and will discuss the strengths, areas for improvement, and identify the nurse's goals for building MI skills over the subsequent quarter. These elements will be entered into REDCap under the process evaluation arm. To ensure inter-rater reliability, a study coordinator who did not provide feedback will also listen to at least one recorded session per site every other quarter and score using the BECCI instrument. Once the BECCI score is completed and feedback is delivered to the study nurse, the recording will be deleted from the digital device and any other local storage location. Only the study team members will have access to the recording device and audio files. BECCI data will also be used in a formative process evaluation at the end of the trial.

For aim 4, each site's study nurse will record at least one ten-minute segment of one study visit with a participant during each study quarter. The same informed consent, recording, and storage procedures will take place as described above.

Qualitative Data

In addition to the quantitative data, we will collect qualitative data for two groups (PLHIV and healthcare team members). Through our experience conducting phone interviews (including phone consent) during the COVID-19 pandemic, we have found this approach to be effective and desirable from the participant's perspective. Therefore, we will take a similar approach for these interviews as described below:

PLHIV and healthcare team member participants will be recruited by the study nurse at each site. The study nurse will ask potentially eligible participants if they are interested in being interviewed by phone. Those who confrim their interest will be contacted by a qualitative researcher who will conduct the interviews for all sites via telephone. The interviewer will perform verbal informed consent using an IRB-approved consent script over the phone, and the participant will be given ample time to ask questions and, if preferred, review the consent script through email. Participants must verbally give their consent before the interview begins. Those who consent will be interviewed during that call, and this process will proceed until each site has reached their sample size for each group.

Names, email address and phone numbers for all study participants who agree to be contacted from all 3 sites (UH, Duke and MetroHealth) will be entered by site research staff in REDCap. The qualitative researcher will use these contact lists to contact patients and track when the interview was performed

All interviews will be recorded and transcribed, and verbal consent will be captured in a consent record as well as in the transcripts. All de-identified data will be stored in a secure location in RedCap and only the qualitative researchers will have access.

Transcription and coding of the interviews will follow the same analysis procedures as laid out in Aim 1. See appendix S, *Key Informant Interview Guides for PLHIV and Clinicians* to review the interview script.

Participants in this qualitative sub-study will receive $25 compensation for participating, which will be distributed to participants in the same manner as other study payments according to local site regulations.

PLHIV (*n*=36): At approximately the time of the final 12-month visit, we will approach a convenience sample of approximately 8-12 participants per site to participate in an additional key informant interview as described below.

<u>*Key Informant Interviews (PLHIV).*</u> The virtual interviews will take place following the 12-month visit at a mutually agreed upon time. Questions will address the participant's perceptions of ASCVD risks associated with HIV, experiences with the EXTRA-CVD intervention and will focus on the RE-AIM domains of ***reach***, ***adoption***, and ***implementation.*** All interviews will take approximately 30 minutes, and audio recordings will later be transcribed verbatim.

Healthcare team members and site prevention nurses (*n*=27): We will recruit approximately 8 healthcare workers per site to provide qualitative feedback on the intervention. These healthcare team member participants will complete a brief self-reported survey consisting of demographics, training, and general practice patterns as well as an in-person key informant interview.

We will additionally invite the prevention nurse from each site (n=3) to participate in a

healthcare team member interview, since they have served as integral members of the healthcare team during the patient's participation in the trial. We have added additional questions to the interview guide (**Appendix S**) that are specific to the prevention nurses.

Key Informant Interviews (Healthcare Team members). In a similar fashion as for the PLHIV sample, we will conduct individual virtual interviews with healthcare team members to understand perceptions of the EXTRA-CVD intervention and will also focus on the RE-AIM domains of ***reach***, ***adoption***, and ***implementation***. These ~30minute interviews will be recorded and later be transcribed verbatim. The interviewer will also collect basic demographics and information about their practice setting.

Outcomes and analysis: Quantitative data (e.g., demographics) will be summarized and used to describe the study samples. After redacting all names and identifying information, verbatim transcriptions of recorded interviews will be entered into Dedoose[76], a secure, website-based analysis program to analyze qualitative data. A quality assurance protocol will be built into data management and analysis; 25% of the transcripts will be check to verify accuracy of the transcriptions and 10% will be double-coded to ensure inter-coder reliability of 80% or greater. Pooled Kappa statistics will be calculated for codes to assess inter-rater agreement.[77]

Under PI-Webel's direction, all responses will be analyzed using standard analytic techniques for qualitative data: identification of themes/domains; coding or classification of participants' responses by these themes performed independently by two team members (who have graduate-level training in qualitative coding); resolution of any coding discrepancies will be done by a third team member.[78] To ensure consistency, a codebook and dictionary will be developed to create universal definitions for each code. The codebook will contain all codes, their definition, and exemplar quotes.

PLHIV-Provider Trust & Communication Ties

The success of the EXTRA-CVD intervention to improve blood pressure and cholesterol may depend on the nature of the personal relationship between PLHIV participants and his/her providers, including the prevention nurse. Therefore, we have designed our own tool, based on validated surveys used in social network analyses, to assess the strength of trust and communication ties between PLHIV participants and providers as well as ties between the prevention nurse and health care providers/staff. There will be three sources of data for this part of the process evaluation:

1. Survey completed by the PLHIV participant about each of his/her healthcare team members. This survey will be completed at each in-person visit. **(Appendix I)**
2. Survey completed by the prevention nurse about each PLHIV participant after each in-person visit. **(Appendix J)**
3. Survey completed by the prevention nurse about providers encountered during the course of the trial (completed every 3 months only for those providers with whom the prevention nurse interacted during that quarter). **(Appendix K)**

Confidentiality of trust and communication survey responses is especially important. To that end, we have designed these surveys in a way that it is not possible to link responses to individual persons as follows:

1. The surveys will not be conducted in redcap. Instead, a Case Western Qualtrics platform will be used. The survey links for all sites will be maintained by one study coordinator at

the UH/Case Western site. No PHI will be entered into this platform.

2. The prevention nurse at each site will keep a key of unique identifiers for each provider within a patient's care team network. The prevention nurse will keep this in a password protected file on his/her own personal network drive (to ensure that it is backed-up). The prevention nurse will be the only one with access to this file.
3. The nurse will prepare the participant who is taking the survey by helping the participant enter the unique identifier for his/her HIV provider, HIV-nurse, primary care provider, or non-HIV specialist. The participant will then complete the same set of survey questions for each of his/her providers.
4. On a quarterly basis, or when not fewer than 10 complete records have accumulated, the UH/Case Western study coordinator will compile the survey data and link it to exported RedCap data using a statistics software program (STATA 13.0).
5. This completely deidentified dataset will then be sent to two consultants with expertise in social network analysis (Dr. Emily Choi, UT Dallas and Dr. Virginie Kidwell-Lopez, U North Texas) who will conduct the analyses.
6. Results will be reported anonymously by site (site A, B, and C), prevention RN (RN1, RN2, RN3) and provider (HIV provider 1, PCP 1, etc…).

Analyses: The primary analysis will be to test the intervention effect on PLHIV-Provider trust and communication ties, using a similar repeated measures analysis as described below for the primary BP and secondary cholesterol outcomes. As an exploratory analysis, we will assess whether the effect of the EXTRA-CVD intervention on the primary BP and secondary non-HDL outcomes are mediated by changes in trust and communication ties. Additional exploratory analyses will be conducted to assess the effect on the balance of trust/communication ties between HIV vs. non-HIV providers and to assess the longitudinal change in prevention RN-provider trust and communication over the course of the intervention.

Aim 4 Procedures:

Intervention Adaptation

The intervention will be adapted with the assistance of stakeholder design teams that have already been convened in Cleveland and Durham as part of our original intervention adaptation sub-aim 1.1. We now propose an additional round of design iteration to make virtual adaptations for this aim. Members of both design teams will be invited to attend combined virtual design sessions hosted in Zoom.

Adaptation Description

During the Zoom meetings, several suggested virtual enhancements will be presented and we will engage in abbreviated exercises to (1) brainstorm, (2) conceptualize, and (3) create new virtual enhancements to the intervention. Small working groups will be assigned to further develop the enhancements into final products offline, using iterative processes consistent with human-centered design principles. The goal of this phase of aim 4 is to present the virtual intervention to the design team and move them through a human-centered design process that will allow them to adapt intervention elements aimed at improving the overall experience and outcomes of participants. Through this process, design team members will: explore the intervention in its current conceptual state; consider how the participants living with HIV may experience the intervention elements; identify potential challenges PLWH may have in positively experiencing the virtual arm of EXTRA-CVD; conceive new ideas and solutions to overcome

those challenges, and; reach consensus on three key areas of adaptation to improve the aim 4 intervention for PLWH before enrollment begins.

Finalized Adaptions

Three potential virtual enhancements to the EXTRA-CVD intervention were presented to the design team for consideration as potential adaptations. These included the following three bulleted items.

- *Virtual Adherence Support Groups:* Peer support groups are a long-standing component of community HIV care that can address social isolation and improve quality of life for PLWH. We propose asynchronous Facebook groups or synchronous Zoom meetings to address social isolation and provide peer-based education to improve CVD self-management behaviors for PLWH in the US.

- *Cardiovascular Prevention Specialist Remote Consultation:* Early qualitative data from studies conducted by our study team suggest PLWH want to access high quality CVD prevention care but face unique barriers to doing so, including stigma and discrimination at visits with specialty providers outside their HIV medical home. One proposed EXTRA-CVD adaptation would be to provide all virtual arm participants at least one remote consultation from a specialist who would provide recommendations for care and consider clinical follow-up as necessary.

- *Community Health Worker Technology Coach:* Evidence suggests that community health workers may be helpful to address psychosocial problems—such as the stress and isolation resulting from social distancing—in PLWH. In addition, people who are unfamiliar with technology such as smart phones and videoconferencing may be able to access them with minimal coaching from technologically savvy family members or friends. We propose considering community based coaches (possibly peers, social workers, or case managers) to provide assistance either through home visits or at other required points of contact with the HIV clinic.

The design team explored the acceptability and feasibility of implementing these above ideas as well as others conceived during the sessions, and opted against prioritizing inclusion of the virtual adherence support groups and CVD prevention specialist consultations into aim 4.

Adaption 1: Study Nurse Technology Coach. The design team did reach consensus for the inclusion of a technology coach into aim 4, however, recommended that the study nurse at each site serve as the coach for participants rather than an added member of the study team. The nurse will serve as a technology coach given that they will already be building rapport with the participant, and this will allow for the participant to easily identify who to reach out to when they need technical support. Each nurse will receive ongoing training and consultation from IT experts who are already providing assistance to the study and/or clinical sites. The nurses will share their built knowledge with the parent study team during monthly bootcamp learning sessions that are already taking place as part of aim 2. The study team has developed an evolving virtual technology and engagement toolkit that will serve as a guide for the nurses from which

they can pull patient-tailored resources and methods to help troubleshoot telehealth issues and improve participant engagement.

Adaptation 2: Virtual Communication Preferences Assessment. The design team recommended that virtual engagement methods integrated into aim 4 be flexible to meet participant needs and include opportunities to build a strong sense of trust and relational connection between the nurse and participants. In response to this concern, the study team has developed two tools that the design team has approved in helping fulfill the needs of the identified adaptation. One tool will be integrated into REDCap and distributed as a survey during their consent visit. This survey, called FACETS (*Appendix O*), is a validated instrument to assess participants' comfort in and access to utilizing various forms of technology. The other tool is a communication preferences form (*Appendix P*) that will be completed as a brief interview with the participant on the day of consent. This form will help the nurse gauge which methods of communication the participant feels most comfortable using to conduct the study visits, receive study-related education, and check in for study-specific follow-ups. The preferences form captures their remote contact information, their self-rating for capacity in utilizing various forms of communication technology, their sense of privacy and safety in communicating on health issues within their home environment, and an open-ended question to understand any concerns they have about communicating with the nurse virtually on health issues.

Adaptation Components

The adaptation phase will include one REDCap survey link to collect demographic information from all design team participants and an organizational readiness assessment for participants who work in the study site HIV clinics. This online survey will take up to 15 minutes to complete. This will be followed by three brief, virtual design team sessions, with one being an at-home assignment and the remaining two Zoom videoconference meetings. The activities will include: 1. An assignment called empathy mapping that members will be sent via email and as a link for each person to complete at their own pace alone, taking approximately one hour of their time; 2. An initial 1.25-hour scheduled Zoom session to discuss the empathy mapping themes and generate ideas to enhance the aim 4 intervention, and; 3. A second and final 1.25-hour Zoom session to reach consensus on and refine three adaptation ideas. For this virtual design team, a focus group discussion on the virtual human-centered design experience will be conducted at the end of the final Zoom session among those who consent to participate.

IRB Approval of Adaptations

Any final adaptations developed by the design team will be shared with the full study team during a monthly study call. In order for adaptations to be integrated into the intervention, they must be approved by the study PIs. Adaptations that result in changes to the procedures outlined in the aim 4 intervention below, or that alter the core components of the intervention, will be submitted to the UH IRB as a protocol modification. **The team will await IRB review and approval of such adaptations before any aim 4 study procedures take place.**

Implementation-Effectiveness Trial

After deeply assessing the impacts of COVID-19 through interviews conducted with EXTRA-CVD participants as described above, we will develop a virtual EXTRA-CVD hypertension intervention that will be well adapted to the post-COVID era. For this hybrid 3 implementation-effectiveness trial, PLWH participants (n=75) on suppressive ART with high BP will be enrolled in the virtually enhanced intervention. Because these subjects are otherwise ineligible for or unable to participate in the parent trial, this supplemental study arm will not poach potentially eligible participants from the parent trial. EXTRA-CVD study visits and components of the intervention will be delivered remotely, and additional remote support tools will be tailored to the HIV clinic context with assistance from the Design Team.

The purpose of this additional study is to increase the impact and scalability of the original EXTRA-CVD intervention by ***assessing*** the social isolation impacts of the COVID pandemic and ***adapting*** it to post-COVID realities in healthcare delivery. We will accomplish this goal without compromising the integrity or feasibility of the parent trial, and will thus increase the likelihood of the overall project to exert a sustained and powerful influence on the field.

Frequency of Contact

For the virtual intervention arm, after in-person written informed consent and a short visit to pick up a home BP monitor, all subsequent interactions will be conducted by telephone or videoconference, with the exception of home visits from a community health worker technology coach. Virtual follow-ups will occur at 2-weeks (Baseline), 4 months, 8 months, and 12 months.

Virtual study visits will be calculated from the date of the baseline enrollment visit in months 4, 8, and 12. Study team members should plan to schedule these visits +/- 14 days from the calculated visit date, or 21 days at the most. Visits outside of the maximum 42-day window will not be scheduled and that visit will be considered missing for that patient.

Intervention Components

The four components of the EXTRA-CVD intervention: (1) nurse-led care coordination, (2) nurse-managed medication protocols and adherence support (3) home BP monitoring, and (4) electronic medical records (EMR) support tools will be adapted to be delivered 100% virtually by telephone or videoconference, with additional supports for social isolation and technology coaching developed during the intervention adaptation phase described above.

The intervention will utilize many of the same procedures as for the parent EXTRA-CVD intervention (aim 2). Given that visits will be limited to remote means, however, the study team will not collect blood pressure or cholesterol outcome measurements. Study nurses will collect participant-reported, at-home BP measures and will provide CVD coaching and care coordination through telephone and/or videoconferencing during the 4-, 8-, and 12-month follow-up visits. For these follow-ups, the study team will send participants the REDCap survey links via e-mail or will administer surveys by telephone. Our team has experience doing this with the EXTRA-CVD study during the early COVID era (March-June 2020), when all study procedures were conducted remotely. These surveys include validated instruments assessing participants' experiences with loneliness, HIV self-management, and COVID-specific risks and lifestyle impacts. The only EXTRA-CVD surveys which will not be administered to aim 4 trial participants are the provider trust and communication surveys, which we believe cannot be

reliably and confidentially administered by telephone. CVD and HIV-related health information will be reviewed by the study nurses before each follow-up visit through the use of each site's electronic medical record (EMR). This study visit preparation using EMR review allows the study nurses to coordinate CVD care and medication management with the health team, and helps the nurse determine participant progress and elements where participants may benefit from CVD coaching during the virtual follow-up visits. Study nurses will coordinate CVD care with the participant's healthcare providers through email and/or EMR support tools, as is being done in aim 2.

Aim 4 Timeline

Invitations will be sent to the design team members in late October 2020, explaining aim 4 and capturing their interest in participating in the adaptation process. The study team expects to begin consenting design team members in November and to begin conducting study activities related to aim 4 adaptation in November and completing in December 2020.

The study team plans to begin recruitment and enrollment for the aim 4 intervention in January 2021 and continue recruitment for six months. In this scenario, the final 12-month follow-up study visits for aim 4 are expected to be completed in June 2022, and the process evaluation exit interviews to be completed by September 2022.

	Nov- Dec 2020	Year 1 Jan – Dec 2021				Year 2 Jan – Dec 2022			
		Q1	Q2	Q3	Q4	Q1	Q2	Q3	Q4
Intervention Adaptation: Design Team Activities	■								
Aim 4 Intervention Recruitment & Baseline Visits		■	■						
4-Month Follow-Up Visits			■	■	■				
8-Month Follow-Up Visits				■	■	■			
12-Month (Final) Follow-Up Visits					■	■	■		
Exit Interviews With Aim 4 Participants					■	■	■	■	

Sources of materials

Information used to identify potentially eligible patients will be obtained using each site's respective electronic medical record system. We will also use patient reported data obtained after consent. We will obtain biometric data (includes lab data, anthropometric and BP measurements) from individuals and their electronic medical record system. Trained and certified professional staff will obtain all data according to detailed study protocols. Data will be collected directly from study participants, medical records, and used specifically for research purposes.

The following human subjects related data elements will be collected for this study, with the source(s) of information noted:

- Subject demographic and clinical characteristics, including age, gender identity, race/ethnicity, income, education level, and marital status (self-report),
- Clinical diagnoses, lab values (lipid panels, creatinine, basic chemistries, liver panel, HbA1c, creatinine kinase, myoglobin), BP values, and progress notes (electronic medical record)
- Communications between care team providers (electronic medical record, telephone logs, work emails)
- Prospectively collected lipid panel (total cholesterol, LDL-C, HDL-C, and triglycerides) for research (venous blood draw; analyzed in clinical lab)
- Prospectively collected BP for research (rigorous BP measurements by trained research assistant according to a validated protocol)
- Subject reported ASCVD risk perceptions and barriers and facilitators to improving ASCVD risk (key informant interviews)
- Subject health-related information: perceived ASCVD risk, general health and comorbidities (self-report survey and electronic medical records)
- Medication use (self-report and electronic medical record)
- Self-monitoring data: BP values (collected by subject)
- Trust and Communications Ties Survey (self-report)
- Observations of study visits performed by another study team member to ensure fidelity

Our study team is extremely prudent in keeping subject data secure and confidential. The control of access to study databases will be managed centrally by IT systems at each clinic site through user passwords linked to appropriate access privileges. This protects forms from unauthorized view and modifications as well as inadvertent loss or damage. Database servers at each clinic site are secured by a firewall as well as through controlled physical access. We will use a REDCap database (projectredcap.org) to store all study data. REDCap has many security protection features that ensure that each person accessing the database has the proper authority to perform the functions he or she requests of the data management system. Within the secondary SAS databases, UNIX group access control will be used for maintaining similar security. University Hospitals REDCap IT will ensure that only IRB-approved individuals on the study team will have access to individually identifiable information about human subjects. This will include the PIs, co-investigators, project coordinator, statisticians, database/programming team, and research assistants. Some of the data above will be accessed from information already collected as part of usual care. All additional data from subjects will be specifically collected for the proposed research project and not a part of clinical care.

Potential risks

Loss of confidentiality. The risks associated with gathering mixed methods data from participants by properly trained and supervised research assistants and technical staff is low and include risks of loss of privacy and psychological distress.

***Detection of clinically significant problems*:** Although not caused by study participation, it is possible that clinically significant problems will be detected by study staff. Subjects entering the

study will have a history of hypertension and hyperlipidemia so we expect to see abnormal systolic and diastolic values, as well as abnormal lipid values. All values that reach a safety threshold (<90 or >180 systolic BP, <40 or >110 diastolic BP) will be reported to the subjects' care provider.

***Kidney disease and electrolyte imbalances*:** Some of the blood pressure agents used in the nurse-managed protocols may cause acute kidney injury and electrolyte imbalances. Subjects with underlying kidney disease at baseline will be at higher risk.

***Other medication side effects**:* All medications have potential side effects. Medications used in the nurse-managed protocols will be only be recommended by the nurse and must ultimately be prescribed by the subject's treating provider according to his/her best clinical judgement and approval. Common side effects of anti-hypertensive and lipid-lowering medication include but are not limited to: bradycardia, lightheadedness and orthostatic hypotension, lower extremity edema, kidney injury and electrolyte imbalances (see above), and myalgias. Rhabdomyolysis, glucose intolerance, and hepatic injury are rare complications of statin therapy.

Physical activity. All subjects will be encouraged to increase their physical activity, raising the possibility of musculoskeletal injury or unmasking of ischemic heart disease. Risks from increased physical activity will be minimized by encouraging moderate rather than vigorous activity. Providers will respond to these patient problems per usual medical practice.

Smoking. While not a key component of the intervention, all subjects will be encouraged to quit smoking (if currently using), raising the possibility of withdrawal symptoms from nicotine dependence.

Psychological risks. We do not anticipate any substantial psychological risks to be associated with participation in this study. As part of our assessments, we will ask participants about their demographic characteristics (i.e., race/ethnicity, socioeconomic status). It is possible that some participants may feel uncomfortable answering some of these questions. We will only ask questions that involve data that are important for study outcomes, and we will inform patients that they may refuse to answer any interview or survey questions, but still be involved in the study. It is also possible that participants may be uncomfortable talking with the Prevention Nurse about some topics that are included in patient-based intervention.

Protections against risk

The specific risk of participation are noted above; procedures for protection follow.

***Protection of participants' identities and confidentiality**:* Because this study involves persons with HIV/AIDS, steps must be taken to protect not only the data they provide, but also their identities. The following confidentiality-protection steps will be taken: [1] All research staff will participate in initial training, follow-up training, and ongoing monitoring and supervision to ensure their understanding of ethical issues involved in this research; [2] consent forms will be maintained in locked files with limited access, separate from any subject data and will only be accessible to the study team; and [3] any personal identifiers linked to data will be removed and replaced by code numbers in all records. These steps are not foolproof, and participants will be informed of the associated risks at the time of informed consent.

Research staff will spend approximately 20 hours in initial training sessions and observed practice. Training includes reading and discussing research protocols and selected articles about

interviewing, tracking, participants and attending lecture sessions regarding emergency procedures, mandatory reporting, confidentiality, and research ethics. Training also will include how to handle transient discomfort or distress related to embarrassing or sensitive discussions as well as how to identify and respond to signs of acute distress; experienced supervisors will be available for immediate consultation in the event of unexpected acute psychological problems; and all staff will be made familiar with referral resources and procedures for psychological, social service, substance-use treatment, and other emergency needs.

Blood Pressure*:* All participants will have a high risk for CVD, and thus many will be prescribed hypertension medications by their health care provider at the outset of the study. It is likely that as a result of increased monitoring, we will detect more episodes of abnormal BP values. Because of potential high and low BP values, subjects in the intervention arm may have their current hypertension regimen adjusted. Safety monitoring of BP will occur in the context of home BP monitoring as well as BP measurement during data collection visits. An average SBP at any study visit or during home blood pressure monitoring > 180 mm Hg and/or diastolic is >110 mm Hg will be considered an alert value and will trigger assessment by the clinician. Furthermore, an average at any study visit or during a home blood pressure monitoring that is < 90 systolic or < 40 diastolic would also be considered an alert value and would trigger an assessment by a clinician. Participants who have an alert reading at home will be asked to contact the clinician directly so that she/he can assess for any cardiovascular symptoms.

Participants who have an alert BP reading during study visits will be directly assessed for cardiovascular symptoms during the visit. Once an alert value has been confirmed the participant will be triaged according to follow-up recommendations from Joint National Committee Recommendations (JNC 8). Participants will have access to their regular providers as well as the following study investigators designated as the clinical contact for each site: Dr. Gripshover (infectious disease physician and HIV clinic director; UH Special Immunology Unit), Dr. Hileman (infectious disease physician and HIV clinic director; MetroHealth Medical Center) and Dr. Okeke (infectious disease physician; Duke Health). If at any time, participants have symptoms of acute end organ damage (i.e. current chest pain, dyspnea at rest, new onset of blurry vision, or new neurological deficits consistent with a stroke) in the context of an elevated BP measurement (SBP >180, and/or DBP >110), participants will be asked to contact the clinician and will be advised and assisted in seeking emergency medical care. For participants in the intervention group whose average SBP >180 and DBP >110 or SBP < 90 or DBP < 40 but are without acute symptoms, the participant's primary provider will be notified and medications will be changed as deemed appropriate by the study team. Follow up contact with the study staff will occur within one week. All abnormal blood pressure results will be communicated to the clinic director at each site who will be an integral part of triage and ensuring follow up. Any change in medication management or observation of an alert value will be communicated from the nurse as soon as possible. The prevention nurse will then generate a note to be entered into the electronic medical record and will communicate directly with the subject's PCP. The research nurses will be integrated parts of their respective health clinic.

Medication adverse effects, including kidney and electrolyte imbalances*:* All participants who are prescribed a clinically indicated new medication according the nurse-managed protocol will have that medication prescribed by the participant's usual health care provider, who will take primary responsibility for counseling the patient about side-effects and ordering follow-up laboratories. In addition, each participant will be counseled by the prevention nurse about

possible side effects and need for any monitoring. These protocols therefore will provide an additional level of monitoring compared to routine clinical care.

Anti-hypertensive medication: Any patient prescribed an ACE-inhibitor, angiotensin receptor blocker, diuretic, or aldosterone antagonist, will be asked to return in 7-10 days for a repeat chemistry panel to check kidney function and electrolytes. The blood pressure algorithm will have special recommendations for those with more advanced chronic kidney disease (eGFR <60). Providers caring for study participants with conditions including but not limited to CKD and ASCVD, will be permitted to take their patients off of any protocolized management. Providers will take primary responsibility for the prescription of any medications in this study.

Lipid lowering medication: When a new lipid-lowering agent is prescribed, the prevention nurse will call 2 weeks after initiation to discuss adherence and any possible side-effects. The nurse will use an evidence-based approach to evaluation and management of muscle symptoms and other intolerances of statins as recommended by NLA guidelines. This will include ordering of creatinine kinase levels or liver function tests when appropriate.

***Unexpected and serious adverse event reporting*:** A detailed monitoring plan will be included as part of the study protocol and submitted to the IRB and reviewed and approved by the funding Institute and Center (IC) before the study begins. Prior to initiation of the study, agreement about the data safety monitoring plan will be confirmed in order to ensure the safety of subjects and the validity and integrity of the data. The prevention nurse or research coordinator at each site will report serious adverse events that are unexpected and study related immediately to a study physician who will convey this information to the study team, IRB, and NIH. All AE's and SAE's will be captured, reports will be completed, and entered into the study database. A safety report will detail all serious and unexpected adverse events or other unanticipated problems that involve risk to study participants or others, and whether these appeared to be related to the study-based interventions or research assessment protocols. If the study team, UH IRB, or NIH has concerns regarding SAEs, the UH IRB will be notified and a copy of the safety summary will be filed with the UH and local site IRBs. Actions taken by the UH or local IRB offices in response to adverse event concerns will be reported to the NIH.

Communications between care team providers and the study prevention nurse (electronic medical record, telephone logs, work emails) will be collected as a key process measure of the intervention. We will only examine telephone logs to and from study phones used exclusively for this research study for the purpose of coordinating care of study participants. Similarly, we will examine emails to and from email addresses created specifically for the study. Only secure, HIPAA compliant hospital email platforms will be used. The purpose of this is to protect against collection of any personal communications that are unrelated to the research study.

Potential Benefits of the Proposed Research to the Subject and Others

Potential benefits for subjects may include improved lifestyle, lower blood pressure, and lower cholesterol with a consequent reduction in cardiovascular risk. In our previous experience, subjects in biobehavioral research studies have generally found participation to be a positive experience and they often feel good about helping provide information that has the potential to help others like them. Potential benefits to others include the possibility that this research will lead to the development of more efficient and effective clinical treatments for patients with

cardiovascular disease, with the expectation that this would lead to consequent reduction in subsequent, cardiovascular complications and death. Given the minimal risks associated with this research, and the potential benefit of the proposed findings, the risks to subjects are reasonable, especially with our plan to protect subjects from these risks. Subjects will be reimbursed for their time spent on study participation.

Importance of Knowledge to be Gained

Reducing cardiovascular disease among PLHIV can prevent cardiovascular complications and death. This study may establish the effectiveness of a population intervention that can be disseminated widely. In particular, this intervention has the potential to be disseminated very broadly in HIV-specialty clinic contexts around the United States, because of the potential for improved outcomes among many of its beneficiaries. The minimal health risks to participants are offset by the potential benefits to them and to society.

Research Participant Remuneration

Participant category	Remuneration
Aim 1 HCP	$25 once
Aim 1 PLHIV	$25 once
Sub-aim 1.1 PLHIV	$25 initial group discussion, $50 for each in-person visit ($125 total over 6 weeks)
Sub-aim 1.1 HCP	$25 once
Aim 2 Clinical Trial (PLHIV)	$50 for baseline and 12-month visits, $25 for 4- and 8-month visits ($150 total)
Aim 2 COVID sub-study	$25 once for phone interview
Aim 3 PLHIV	$25 once
Aim 3 HCP	$25 once
Aim 4 Design Team	$50 x 2 Zoom meetings + $50 at home activity = $150 total
Aim 4 AAIM High Trial (PLHIV)	$50 for baseline and 12-month visits, $25 for 4- and 8-month visits ($150 total)

HCP, healthcare provider; PLHIV, person living with HIV

Alternatives to Participation

Participation in the study is voluntary. If a potential participant chooses not to participate, he/she will receive usual care from their HIV specialty care clinic.

Sharing of Results with Research Participants:

As an implementation study that aims to improve clinical care of patients, any relevant clinical data will be shared with patient participants and/or their clinical care team as they would typically be shared through the course of clinical care. This primarily consists of home blood pressure data (intervention arm) and lipid panels, which will result in the electronic health record for both intervention and control participants. Because of the need to maintain the integrity of

the control condition, the prevention nurse will not directly communicate blood pressure or cholesterol results to the control group participants or their providers unless they exceed the safety thresholds defined above. Results of surveys acquired for research purposes only will not be shared directly with participants. At the end of the trial, final aggregate primary results will be disseminated to participants through presentations to local community advisory boards and/or clinic staff at the local sites.

Data Safety Monitoring:

All elements of the data and safety monitoring plan will be reviewed by the IRB at UH and provided to the NIH institute overseeing the project. The study PIs—Dr. Longenecker, Dr. Webel, and Dr. Bosworth together with the UH IRB PI Dr. Gripshover—will share responsibility for monitoring risks to human subjects and implementation of the monitoring plan. The project will utilize existing medical referral sources and physicians knowledgeable about HIV to address any physical risks that might arise. Serious adverse events will be reported promptly to the institution's IRB and project officer of the funding source. Monitoring procedures and reporting and action plans for data and safety-related risks are described below. The Data and Safety Monitoring Plan includes the following components which will be addressed individually below:

- Regulatory Issues
- Data Safety and Monitoring Board
- Data Validity/Integrity
- Protocol Compliance
- Termination for Significant Risk

Each of these components have multiple procedures to safeguard the wellbeing of study subjects and to maintain the scientific integrity of the project while achieving the study's specific aims. Key components of the data and safety monitoring plan include weekly meetings of core members of the research team (at minimum, the Principal Investigators, Project Director and Research Assistants) and monthly meetings of the extended research team including Dr. Longenecker, Dr. Gripshover, Dr. Webel, Dr. Bosworth, Dr. Okeke, Dr. Bloomfield, Dr. Smith, Dr. Vedanthan, site prevention nurses and research coordinators.

Meetings of Core Members of the Research Team:

The PIs will run monthly conference calls with the entire study team which will include core protocol team along with study coordinators, prevention nurses, and other representatives from each of the 3 study sites. The agenda for these meetings will include tracking of subject recruitment, enrollment, and retention; data collection and entry; and documentation and review of any subject concerns or adverse effects. These meetings will help ensure that the project timeline is being met.

Regulatory Issues

Adverse Events Protocols:

An adverse event is defined as any reaction, side effect, diagnosis or untoward event that either a) occurs during the course of the clinical trial and was not present at baseline; or b) was

present at baseline and appears to worsen during the study. All AE's will be reported to one of the study PIs. All subjects will be aware of their rights prior to participation and will be encouraged to report any incidents or adverse effects to the investigators and the Case Western Reserve University Institutional Review Board (IRB). Contact information for the investigators and the IRB will be provided in all the consent materials. During assessments the research staff will inquire about AEs and complete an AE form for each subject. In the event that the participant is experiencing a worsening of symptoms, the Research Assistant will, with participant's permission, inform the clinic director. The study PIs, and research staff will together determine if the AE places the participant at risk if study treatment is continued.

All adverse events, with the exception of clinically insignificant events and minor common illnesses and injuries (e.g., cold/flu, scrapes, upset stomach, low-grade headaches) will be documented on an AE Log. Should any study subjects express concerns about the study and/or their participation in the study or express distress as a result of their participation, the witnessing research team member will document this in the AE Log. AEs will be reported to the study PIs and the study research staff to determine whether and what actions might be appropriate. Any AEs determined to be serious and/or study-related will require the completion of an AE form to be entered into the project database.

During research staff training, procedures for AE identification, collection and reporting will be reviewed in detail. Study staff will be trained to provide crisis intervention and referral as is standard operating procedure within each clinic for such situations, should they become dangerous or life-threatening (i.e. suicidal ideation or attempts).

AEs will be managed in conjunction with clinic medical staff, with permission from the participant. All subjects will be ongoing patients at a study site clinic and thus subjects can be monitored and have access to medical staff throughout the study period.

Data and Safety Monitoring Board

According to guidelines established by the NIH for clinical trials, the investigators will establish a Data and Safety Monitoring board (DSMB) composed of independent research scientists not otherwise connected with the study. This committee (which is independent of the study investigators) will be set up to monitor the study results for evidence of adverse or beneficial treatment effects throughout the study period. The Monitoring Committee will remain "blinded" to outcome characteristics of the study for as long as possible. While the committee may have access to information that is deemed necessary to make an appropriate determination, highly sensitive information in relation to the outcome of the study will be requested on a "need to know" basis as it may arise during the course of the committee's deliberations. The committee's concerns will be directed to patient accrual rates, appropriate follow-up, compliance, data acquisition, undue complications, and whether the study as it is currently being conducted will be able to answer the proposed hypotheses. Additional responsibilities will include monitoring the integrity of data collection, reviewing training and compliance with all components of the interventions and monitoring for adverse events. The membership and frequency of meeting are at the discretion of the funding institute but will consist of 3-5 members with appropriate expertise, for example in biostatistics, cardiovascular disease, behavioral interventions, HIV research, and clinical trials. It is expected that this committee will meet once per year in person and once via conference call and will report to the funding institute on scientific and administrative issues. We do not anticipate any real harm to patients, but adverse

events will be monitored. Adverse event forms will be used to report all unanticipated events. The following information will be included in the report: date of event, attribution to intervention, and outcome of adverse events. Death will be reported within 24 hours. Unanticipated adverse events will be reported within 7 days. Reports will be submitted electronically to the CWRU IRB and NIH. A written follow-up will be submitted within 30 calendar days. All adverse events (serious or not, related or unrelated, anticipated or unanticipated) will be reported in the annual report to the CWRU IRB and NIH.

The DSMB members and contact information is shown in the following table (members appointed Jan 2019):

MEMBER NAME	EMAIL	EXPERTISE
**Ann Avery, MD *** MetroHealth (Cleveland, OH)	aavery@metrohealth.org	HIV Medicine
**Matthew Feinstein, MD** Northwestern (Chicago, IL)	matthewjfeinstein@northwestern.edu	Cardiology
**J. Craig Phillips, LLM, PhD, RN, APRN, ACRN, FAAN** Univ of Ottawa (Ottawa, Canada)	Craig.Phillips@uottawa.ca	Nursing and Human Rights Law
**Steven Grambow, PhD** Duke University (Durham, NC)	steven.grambow@duke.edu	Epidemiology and Statistics

* Chair

Termination for Significant Risk

Diligent monitoring will occur as specified above under Adverse Events Protocols. A participant that expresses concern about his or her participation or reports distress associated with the research procedures may be asked to discontinue participation in the study if there is concern about participant safety and wellbeing or about the safety and wellbeing of others. Likewise, if one of the research assistants or supervising members of the Research Team expresses a concern about a participant's safety or wellbeing, the Adverse Events Protocol would be used and it is possible that the participant could be asked to discontinue participation if there were concern about his or her, or others', safety and wellbeing.

Data Validity/Integrity:

Recruitment:

Participant recruitment will be tracked and reviewed in the weekly meetings of the research team. In order to maintain confidentiality, the list, which includes the names of all potential subjects, will be kept separately from the documentation and tracking spreadsheet in a secure folder. Basic demographic information, as well as reasons for refusal, will be noted for eligible individuals who decline participation. Tracking will be carried out to determine which

and how many individuals are interested in study participation, are eligible for study participation, enroll in the study, and complete the first assessments.

Enrolled subjects will be given code numbers and their study participation will continue to be tracked via spreadsheet, which will include documenting the occurrence and date of completion of data collection.

Consistency of Data Collection:

Data collection will follow study procedures. Data collection will be tracked as specified above and will be monitored in the weekly meetings of key members of the research team. Any issues that arise can therefore be dealt with in a timely manner. Weekly meetings will also include review of upcoming data collection so questionnaires can be prepared in a timely manner.

Quality Assurance:

Drs. Longenecker, Webel and Bosworth will work closely with the statistician (Dr. Smith) to design forms and a database that maximize accurate data entry. Common data elements will use the standardized codes and attempts will be made to harmonize all database codes with NIH initiatives. To minimize data-entry error and data-management miscoding, questionnaire data will be collected via tablet computers and immediately uploaded to a secure web-based server, ensuring backup. The electronic files from all sites will be merged into one REDCap data management file. Summary results will be entered into the larger REDCap database. Source documents and electronic data will be checked for accuracy and adherence to study protocols.

Data Storage:

Consent forms and subject lists will be stored in locked file cabinets separate from subject data and on password-protected computers, and will be only accessible to the research team. All subject data will be labeled only with a code number. These coded data will be kept separate from the master list that links subjects and their code numbers. All subject data will be kept in locked file cabinets in a locked office. Coded data stored on the computer will be maintained in password-restricted files. Any breach in confidentiality will be reported to the PIs.

Statistical Considerations

The primary outcome will be systolic BP at 12 months and secondary outcome will be non-HDL cholesterol at 12 months, both measured at 4 time-points (0, 4, 8, and 12 months). All BPs used for outcomes will be obtained by a blinded research assistant and cholesterol levels will be measured by lab personnel who are also blinded to treatment group. Because the outcomes are continuous, linear mixed-effects models[89] (LMM) will be used to examine the differences over time between the study arms. LMM will allow us to implicitly account for the correlation between a patient's repeated measurements over time. The general mean structure of the LMM we will use to examine the hypotheses is, $Y_{ij} = \beta_0 + \beta_1 * I(month = 4) + \beta_2 * I(month = 8) + \beta_3 * I(month = 12) + \beta_5 * arm * I(month = 4) + \beta_6 * arm * I(month = 8) + \beta_7 * arm * I(month12)$, where Y_{ij} represents the outcome of interest (i.e., SBP or non-HDL) for patient i at time j. In this model, we fit a common intercept and arm is the intervention group indicator. Similarly, time is classified, where for example, $I(month = 12)$ is a dummy variable equal to 1 for the 12 month time point. Random intercepts will be included for

each individual to account for correlation among repeated measurements over time. The primary analytic model will adjust for clinic site. The mixed effects model parameters will be estimated and tested using SAS PROC MIXED (SAS Institute, Cary, NC), and the hypothesis of between-arm differences over time will be tested using estimate statements within PROC MIXED. In particular, β_7, the estimated difference in outcome between arms at 12 months, will be the primary effectiveness outcome assessed.

For the tertiary outcome of cascade category, we will calculate an ordinal four-level variable at baseline, 4, 8, and 12 months. We will use a proportional odds model fit via generalized estimating equations to examine differences over time between study arms. The proportional odds assumption will be assessed using score tests, and the model will be relaxed to partial proportional odds if necessary. Robust sandwich standard errors will be used to account for potential overdispersion and correlation among the repeated observations over time. Similarly to the primary analysis, the model will adjust for clinic site and include the same general mean model specification. The primary hypothesis will be tested via SAS PROC GENMOD to assess whether the estimated proportions in each level at 12 months differ between arms. All analyses will be conducted following an intention to treat (ITT) principle.

Missing Data:

We will assess mechanisms for missing data in this study. LMM, implicitly accommodates missingness when the response is Missing At Random (MAR); that is, when missingness is due either to treatment, to prior outcome, or to other baseline covariates included in the LMM. We will address missing data by imputing missing values using multiple imputation procedures as described by Schafer. Once missing values have been imputed, each multiply-imputed data set can be analyzed using the LMM. Final parameter estimates and their standard errors will be calculated using Rubin's formula. We will analyze our data and report final study results with and without employing the multiple imputation strategy (compared to complete case analysis) and carefully examine and describe any discrepancies found.

Power:

The power calculation for this study was based on our prior nurse-led BP intervention[12], a meta-analysis of cholesterol medication adherence interventions[42], and baseline blood pressure and cholesterol data from our clinic sites. Power estimates were derived empirically via simulation in SAS 9.4. Simulated data were generated based on estimates from prior studies, such that we assumed a mean SBP at baseline of 145mmHg for both arms, with a reduction in the education control arm of 1mmHg by 12 months For the intervention arm, we evaluated effect sizes (differences from education control at 12 months) of 5-7mmHg. We estimate that 15% of patients may drop out by the 12-month time point, and incorporated missing values into the simulated data based on a uniform pattern of 5% missing at 4 months, 10% at 8 months, and 15% at 12 months. The drop-out rate is consistent with prior interventional studies at our sites (80-88% retention at 12 months).[11,12,90] We conservatively estimated variance components assuming a total standard deviation of 17 and a within-individual correlation of 0.4 among repeated SBP measurements. Similarly, for the secondary non-HDL outcome, we assumed a baseline value of 132mg/dL with a standard deviation of 41 and a within-individual correlation of 0.7, and evaluated sample size needed over effect sizes ranging from 10-20mg/dL.

After generating 1,000 simulated datasets under these assumptions, we fit the LMMs described above to each and assessed the effect of interest using two-sided tests with a type I

error rate of 0.05. Based on results, we will have >80% power to detect a 6mmhg lower systolic BP and >90% power to detect a 15mg/dL lower non-HDL cholesterol in the intervention arm vs. education control. Table 5 displays the sample sizes needed to detect a range of plausible *clinically significant* BP and non-HDL effects. A 6 mmHg improvement in systolic BP is associated with a ~20% decrease in ASCVD events[91], and a 15mg/dL improvement in cholesterol is associated with ~10% decrease in clinical ASCVD events[31].

Table 5: Sample size estimates to detect a range of plausible and *clinically significant* effect sizes

	BP Effect Size			*Non-HDL Effect Size*		
	5mmHg	*6mmHg*	*7mmHg*	*10mg/dL*	*15mg/dL*	*20mg/dL*
70% Power	278	190	140	248	110	64
80% Power	350	234	178	310	148	80
90% Power	466	340	232	424	184	104

Green cells represent sample sizes that are less than our proposed sample size (n=300).

Pre-specified sub-group analyses of the primary, secondary, and tertiary outcomes will include clinic site, sex, and baseline ASCVD risk (<20% 10-year risk[92] vs. >20% or prior ASCVD). For each category, we will examine the interactions with intervention arm and time. Generally, the modeling approach will mirror that described above for each outcome. Three separate analyses for each outcome will be conducted to assess the effect of each potential moderator. Models will be fit in SAS PROC MIXED and GENMOD, as described above, and the moderating effect of each of the three factors will be assessed via the hypothesis test of the three-way interaction among subgroup, treatment, and time at 12 months.

ClinicalTrials.gov requirements

This application includes a trial that requires registration in ClinicalTrials.gov. The registration number for this trial is NCT03643705.

Multiple Principal Investigator Leadership Plan

This project will have three NIH co-Principal Investigators (co-PI) and a fourth University Hospitals Principal Investigator to oversee the IRB:

1. **Dr. Chris Longenecker, MD (NIH contact PI)**—Associate Professor of Medicine; University of Washington School of Medicine; Seattle, WA
2. **Dr. Hayden Bosworth, PhD (NIH MPI)**—Research Professor of Population Health, Medicine, Psychiatry, and Nursing; Department of Population Health Sciences; Duke University School of Medicine; Durham, NC
3. **Dr. Allison Webel, RN PhD (NIH MPI)**— Professor of Nursing; University of Washington School of Nursing; Seattle, WA
4. **Dr. Barb Gripshover, MD (UH IRB PI)**—Professor of Medicine; University Hospitals Cleveland Medical Center and Case Western Reserve University School of Medicine; Cleveland, OH

Rationale: We have chosen the multiple PI approach because developing innovative strategies to address the increasing burden of cardiovascular risk among people living with HIV (PLHIV) requires collaborative, interdisciplinary research. Furthermore, the two junior co-PI's (Longenecker and Webel) who are emerging as independent scientists in their field, will benefit from the experience of a senior implementation scientist (Bosworth) with a proven track record developing successful and scalable non-physician-led interventions to address cardiovascular risk factors in the VA and general US population.

Roles and Responsibility: Dr. Longenecker will be the contact PI and will be responsible for communication between the PIs and the NIH**. Dr. Gripshover** will serve as the site PI for UH and take responsibility for logistical study operations at the University Hospitals site and will serve as the primary clinical contact person for that site (similar to the role that co-I's Dr. Hileman (MetroHealth) and Dr. Okeke (Duke) will perform at their respective sites). After 11/1/2021, Dr. Gripshover will also assume other regulatory, training, or enrollment responsibilities at the UH site. **Dr. Bosworth** will take primary responsibility for overseeing the conduct of the clinical trial (aim 2) and will supervise logistical operations for all 3 aims at the Duke site in conjunction with Dr. Okeke. Dr. Bosworth will supervise data collection for aim 2 and has a long track record of collaboration with the project statistician (Dr. Smith) as well as with Dr. Okeke. **Dr. Webel** will take primary responsibility for supervising the qualitative research conducted for the baseline assessment (aim 1) and for the process evaluation (aim 3). The budget allocations have been assigned according to these roles and the effort required to fulfill them (see budget justification). The study PIs will share responsibility for monitoring risks to human subjects. Publication oversight will be based on the relative contributions of all team members, with final decisions regarding first and senior authorship to be made jointly and with consensus by the 3 co-PIs. In the unlikely event of a disagreement, the core protocol team will vote to resolve the disagreement as described below.

Governance: The leadership responsibilities for the study team will be shared by the three co-PIs as described above. Additional members of the core protocol team include the following co-investigators: Dr. Corri Hileman (ID/HIV specialist and MetroHealth HIV clinic director; R01-funded physician scientist studying CVD co-morbidity in HIV; 8 years of multiple collaboration with PI Longenecker), Dr. Lance Okeke (ID/HIV specialist at Duke; studies CVD co-morbidity in HIV and mentored by PI Bosworth), Dr. Gerald Bloomfield (Duke cardiologist; expert on CVD in HIV, multiple collaborations with PI Longenecker), Dr. Rajesh Vedanthan (NYU cardiologist; NHLBI R01-funded implementation scientist; prior collaborations with Dr. Bloomfield and Dr. Longenecker). Given PI Longenecker's transition to UW, site PI Gripshover will assume on-site responsibilities at UH. If any additional PIs move to a new institution, attempts will be made to transfer the relevant portion of the grant to the new institution. In the event that a PI cannot carry out his/her duties, a new PI with appropriate expertise and skills will be recruited as a replacement at one of the participating institutions.

Communication: The co-PIs will run monthly conference calls with the entire study team which will include core protocol team along with study coordinators, prevention nurses, and other representatives from each of the 3 study sites. Dr. Longenecker and Webel have offices within <5min walking distance of each other on the UW campus. The entire study team will meet at a critical juncture at the end of Year 1 for a team meeting in Cleveland in order to promote healthy team dynamics and to conduct some of the study aims as described in the research strategy (initial presentations to the sub-aim 1.2 design team, training for aim 2, etc…).

Decision Making and Conflict Resolution: Decision-making about study conduct and scientific direction will be made by the core protocol team. We anticipate that any minor conflicts that arise will be resolved through direct communication, and we plan to use that as our main approach. The team will mediate any conflicts between study PIs, in the unlikely event that the conflict cannot be resolved with direct communication. For issues that only affect this proposal and where voting is appropriate, a simple majority of the core team will rule. The DSMB also is available for mediation if an issue arises regarding subject safety.

13. Timeline:

**Timeline**	Year 1				Year 2				Year 3				Year 4			
	Q1	Q2	Q3	Q4	Q1	Q2	Q3	Q4	Q1	Q2	Q3	Q4	Q1	Q2	Q3	Q4
**Administrative**																
Develop protocols and study forms	■															
Train staff	■	■	■	■												
Team meeting in Cleveland				x												
**Aim 1**																
Mixed-methods data collection		■	■													
Analyses			■	■	■											
**Sub-aim 1.1:**																
Development			■	■												
Acceptability & Feasibility Testing				■	■											
**Aim 2**																
Enrollment					■	■	■	■	■							
Follow-up assessments						■	■	■	■	■	■	■	■			
Analyses														■	■	■
**Aim 3**																
Process evaluation data collection					■	■	■	■	■	■	■	■	■			
Perceived risk and network surveys					■	■	■	■	■	■	■	■	■			
Qualitative studies													■	■		
Analyses														■	■	■
**Aim 4**																
Virtual intervention adaptation											■	■				
Implementation – effectiveness trial												■	■	■	■	■
**Research Products**																
Scientific meetings & publications				x		x		x		x		x		x		x

Appendix A: Health Beliefs for Cardiovascular Disease Survey

	Strongly Disagree			Strongly Agree
1.It is likely that I will suffer from a heart attack or stroke in the future	1	2	3	4
2. My chances of suffering from a heart attack or stroke in the next few years are great	1	2	3	4
3. I feel I will have a heart attack or stroke sometime during my life	1	2	3	4
4. Having a heart attack or stroke is currently a possibility for me	1	2	3	4
5. I am concerned about the likelihood of having a heart attack or stroke in the near future	1	2	3	4
6. having a heart attack or stroke is always fatal	1	2	3	4
7. Having a heart attack or stroke will threaten my relationship with my significant other	1	2	3	4
8. My whole life would change if I had a heart attack or stroke	1	2	3	4
9. Having a heart attack or stroke would have a very bad effect on my sex life	1	2	3	4
10. If I have a heart attack or stroke I will die within 10 years	1	2	3	4
11. Increasing my exercise will decrease my chances of having a heart attack or stroke	1	2	3	4
12. Eating a healthy diet will decrease my chance of having a heart attack or stroke	1	2	3	4
13. Eating a healthy diet and exercising for 30 minutes most days will help to prevent a heart attack or stroke	1	2	3	4
14. When I exercise I am doing something good for myself	1	2	3	4
15. When I eat healthy I am doing something good for myself	1	2	3	4
16. Eating a healthy diet will decrease my chances of dying from cardiovascular disease	1	2	3	4
17. I don't know appropriate exercises to perform to reduce my risk of developing cardiovascular disease	1	2	3	4
18. It is painful for me to walk for more than 5 minutes walking	1	2	3	4
19. I have access to exercise facilities and or equipment	1	2	3	4
20. I have someone who will exercise with me	1	2	3	4

21. I don’t have time to exercise for 30 minutes a day on most days of the week	1	2	3	4
22. I don’t know what is considered a healthy diet that would prevent me from developing cardiovascular disease	1	2	3	4
23. I don’t have time to cook meals for myself	1	2	3	4
24. I cannot afford to buy healthy foods	1	2	3	4
25. I have other problems more important than worrying about diet and exercise	1	2	3	4

Appendix B: Adherence to Hypertension and Cholesterol Medication Scales

Adherence for blood pressure medication

In order for blood pressure medication to work, people have to take it according to their doctor's instructions. For one reason or another, people can't or don't always take all of their pills as prescribed. We want to know how often you have missed your blood pressure medication. Please rate your agreement with the following statements.

Over the past 7 days…

1. I took my blood pressure medication as prescribed.	Strongly disagree ○	○	○	○	○ Strongly agree
2. I missed or skipped at least one dose of my blood pressure medication.	Strongly disagree ○	○	○	○	○ Strongly agree
3. I was not able to take all of my blood pressure medication.	Strongly disagree ○	○	○	○	○ Strongly agree

Scoring instructions: nonadherence scale score - calculating the mean of the three extents of nonadherence items. Higher scores indicate greater levels of non-adherence

Reasons for Nonadherence to Blood Pressure Medicines

Situations come up that make it difficult for people to take their blood pressure medications as prescribed by their doctors. Below is a list of those situations. We want to know how much these situations contributed to you missing a dose of your medication. Only one of these situations may apply to you, or many may apply to you.

In the past 7 days, how much did each situation contribute to you missing a dose of your blood pressure medication?

	Not at all				Very much
1. I was busy	○	○	○	○	○
2. There was no one to remind me	○	○	○	○	○
3. They caused some side effects	○	○	○	○	○
4. I worried about taking them for the rest of my life	○	○	○	○	○
5. They cost a lot of money	○	○	○	○	○
6. I came home late	○	○	○	○	○
7. I did not have any symptoms of high blood pressure	○	○	○	○	○
8. I was with friends or family members	○	○	○	○	○
9. I was in a public place	○	○	○	○	○

10. I was afraid of becoming dependent on them	Not at all ○	○	○	○	○	Very much
11. I was afraid they may affect my sexual performance	Not at all ○	○	○	○	○	Very much
12. The time to take them was between my meals	Not at all ○	○	○	○	○	Very much
13. I felt I did not need them	Not at all ○	○	○	○	○	Very much
14. I was travelling	Not at all ○	○	○	○	○	Very much
15. I was supposed to take them more than once a day	Not at all ○	○	○	○	○	Very much
16. I had other medications to take	Not at all ○	○	○	○	○	Very much
17. They make me want to urinate while away from home	Not at all ○	○	○	○	○	Very much
18. I ran out of medication	Not at all ○	○	○	○	○	Very much
19. I was afraid the medication would interact with other medication I take	Not at all ○	○	○	○	○	Very much
20. My blood pressure was too low	Not at all ○	○	○	○	○	Very much
21. I was feeling too ill to take them	Not at all ○	○	○	○	○	Very much

Scoring instructions: These items are used individually; no total score is computed.

Adherence for cholesterol lowering medication

In order for cholesterol medication to work, people have to take it according to their doctor's instructions. For one reason or another, people can't or don't always take all of their pills as prescribed. We want to know how often you have missed your cholesterol medication. Please rate your agreement with the following statements.

Over the past 7 days…

4. I took my cholesterol medications as prescribed.	Strongly disagree ○	○	○	○	○	Strongly agree
5. I missed or skipped at least one dose of my cholesterol medications.	Strongly disagree ○	○	○	○	○	Strongly agree
6. I was not able to take all of my cholesterol medication.	Strongly disagree ○	○	○	○	○	Strongly agree

Scoring instructions: nonadherence scale score - calculating the mean of the three extents of nonadherence items. Higher scores indicate greater levels of non-adherence

Reasons for Nonadherence to Cholesterol Medicines

Situations come up that make it difficult for people to take their cholesterol lowering medication as prescribed by their doctors. Below is a list of those situations. We want to know how much these situations contributed to you missing a dose of your medication. Only one of these situations may apply to you, or many may apply to you.

In the past 7 days, how much did each situation contribute to you missing a dose of your cholesterol medication?

	Not at all				Very much
1. I was busy	○	○	○	○	○
2. I forgot	○	○	○	○	○
3. The medication caused some side effects.	○	○	○	○	○
4. I worried about taking them for the rest of my life	○	○	○	○	○
5. They cost a lot of money	○	○	○	○	○
6. I came home late	○	○	○	○	○
7. I did not have any symptoms of high cholesterol	○	○	○	○	○
8. I was with friends or family members	○	○	○	○	○
9. I was in a public place	○	○	○	○	○

10. I was afraid of becoming dependent on them	Not at all ○ ○ ○ ○ ○ Very much	
11. I was afraid they may affect my liver.	Not at all ○ ○ ○ ○ ○ Very much	
12. The time to take them was between my meals	Not at all ○ ○ ○ ○ ○ Very much	
13. I felt I did not need them	Not at all ○ ○ ○ ○ ○ Very much	
14. I was travelling	Not at all ○ ○ ○ ○ ○ Very much	
15. I was supposed to take them too many times a day.	Not at all ○ ○ ○ ○ ○ Very much	
16. I had other medications to take	Not at all ○ ○ ○ ○ ○ Very much	
17. I was afraid they would cause muscle pain.	Not at all ○ ○ ○ ○ ○ Very much	
18. I ran out of medication	Not at all ○ ○ ○ ○ ○ Very much	
19. I was afraid the medication would interact with other medication I take	Not at all ○ ○ ○ ○ ○ Very much	
20. My cholesterol was too low.	Not at all ○ ○ ○ ○ ○ Very much	
21. I was feeling too ill to take them.	Not at all ○ ○ ○ ○ ○ Very much	

Scoring instructions: These items are used individually; no total score is computed.

Appendix C: Interview Guides AIM 1

Key Informant Interview Guide for Patients Living with HIV

Introduction

[Remind about Audio Recording and not to use real names]

Thank you for talking with us today. We are interested in learning more about your thoughts and beliefs about cardiovascular or heart disease. I am going to ask you some questions about these topics to hear about your experiences and perspectives. Please know that there is no right or wrong answer. You will notice that I won't give you a lot of feedback on your responses because I don't want to influence your answers. You may also notice that I will jot things down on paper while you talk- this is simply a reminder to ask you a follow-up question. Finally, you are under no obligation to talk about anything that you are not comfortable discussing with me. Do you have any questions or concerns before we begin?

Primary Questions

General CVD Perceptions

Let's start by you telling me about what you know about cardiovascular or heart disease. Prompts: What is heart disease; what causes heart disease; how do you prevent it; how do you treat it if you get it; how do you know if you have it?

Do you think you are at risk for developing heart disease? Why? Prompts: Family history, past or current substance use, obesity, inactivity, diet, medications you take

What do you know about the risk factors for stroke? Are they similar or different from other heart disease risk factors?

When you think about your passing, what do you think you will eventually die from? (e.g., AIDS, heart disease or something else)

Have any of your doctors or nurses ever talked with you about heart disease? If yes, what did they tell you? Have you ever asked your HIV doctor about your risk of developing heart disease? Do you have a separate primary care doctor (from your HIV doctor)? If so, did have they ever talked with you your risk for heart disease?

Have you ever been evaluated for heart disease by a doctor? If so, what did he or she do? Was it your HIV doctor or another doctor? What led to that evaluation (e.g., symptoms (chest pain, swelling, etc…)

What are the medications that are used to prevent heart attacks and strokes?

Do you tend to be more worried about side-effects of medications or are you more worried about what will happen if you don't take the medications?

Tell me about a time when your doctor or nurse has talked with you about your risk for having a heart attack or heart problems? Probes: What prompted that discussion (e.g., did you bring it up, was it part of a research study, did you have abnormal blood pressure or cholesterol?)

Have you been prescribed medications for blood pressure or cholesterol? Do you know what the medication is? How often to do you take it? What makes it hard to take it? Easy?

Do you check your blood pressure at home or anywhere outside of a doctor's office? If so, how often do you check it?

What else do you do to help prevent heart problems? Who or what helps you do that?

HIV and CVD

Do you think HIV affects your risk for heart disease? If so, how?

Has anyone in your doctor's office ever talked with you about the relationship between HIV and heart disease? Who? What did they tell you? How did you respond to that conversation? Did it impact how you take care of yourself? How?

What HIV medications are you taking? Have you ever heard that some HIV medications can increase your risk of developing heart disease while others do not impact this risk? Where and from whom?

If you knew that HIV medication increases your heart disease risk, would this impact your behavior (e.g., smoking, diet, exercise, taking medications, seeing/talking with your doctor).

Are you concerned about other medications interacting with your HIV drugs? If a non-HIV doctor prescribes a medication for you, do you always check with your HIV doctor before you agree to take it?

Would you be more likely to take a preventive medication (like aspirin or statins) if your HIV doctor prescribed it compared to a non-HIV provider? Why or why not?

Intervention Tailoring

Tell me about a time you improved your health behavior. Who helped you achieve this improvement? What steps did you take to make the change? How long did it last?

Do you ever track anything at home, like your weight or your mood? What would make it easier for you to do so? What if we gave you a kit to monitor your blood pressure? Do you think you could measure it every day? What would be hard about that? What would make it easier?

Other than your doctor, where do you get information about your health? What sources do you trust the most? What sorts of health messages or education do you prefer? What messages motivate or inspire you to change your health behaviors.

What sort of intervention or program might help you improve heart disease prevention behaviors such as smoking cessation, eating a healthier diet, exercising harder and more often, talking with your doctor about how you can determine your own risk for heart disease and take steps to reduce that risk?

Do you like messages that focus on one health behavior change (such as smoking cessation or improving you diet) or multiple changes?

Do you prefer paper, verbal (conversations), digital (phone, computer) or visual (TV) messages to help you understand health information the best and how to best act on that information to improve your health?

Secondary/Follow-up Questions

Follow-up “Probes” after significant statements are made:

[Earlier/A moment ago/when you first started speaking/when you were talking about x] you said [significant statement].

- Can you tell me more about that?
- Can you tell me more about how that affects [X]
- Can you clarify what you mean by [significant statement]
- Can you give me an example of a time when [significant statement] happened to you

Conclusion

Is there anything else you want us to know about [X]? (YES return to interview; NO proceed)

We want to thank you so much for your participation and remind you that everything we have discussed will remain private. The audio file will be destroyed once this interview is transcribed, and the transcription will not contain your name or any identifying information.

Key Informant Interview Guide for Clinicians

Introduction

[Remind about Audio Recording and not to use real names]

Thank you for talking with us today. We are interested in learning more about your thoughts and beliefs about cardiovascular disease among your HIV+ patients. We are most interested in the patients you see in their routine, outpatient HIV care, not those in the hospital. I am going to ask you some questions about these topics to hear about your experiences and perspectives. Please know that there is no right or wrong answer. You will notice that I won't give you a lot of feedback on your responses because I don't want to influence your answers. You may also notice that I will jot things down on paper while you talk- this is simply a reminder to ask you a follow-up question. Finally, you are under no obligation to talk about anything that you are not comfortable discussing with me. Do you have any questions or concerns before we begin?

Primary Questions for all Clinicians

General CVD Perceptions

- Let's start by you telling me about what you think the biggest health problems are for your HIV+ patients.
- Can you tell me what you know about cardiovascular or heart disease and HIV. Prompts: Are your HIV+ patients at risk for developing heart disease? Why? Prompts: Family history, past or current substance use, obesity, inactivity, diet, medications you take
- When you think about your patients passing away, what do you think you will eventually die from? (e.g., AIDS, heart disease or something else)
- Have you ever talked with your patients about heart disease? If yes, tell me about that/those conversation(s)? Prompts: What prompted that discussion (e.g., did you bring it up, was it part of a research study, did you have abnormal blood pressure or cholesterol?) Did they ask you about their risk of developing heart disease? Did their primary care provider reach out to you?
- What are the medications that are used to prevent heart attacks and strokes?
- What can do you do to help prevent heart problems in your HIV+ patients? Who or what helps you do that?

HIV and CVD

- Do you think HIV affects your patient's risk for heart disease? If so, how?
- Have you ever heard that some HIV medications can increase your patient's risk of developing heart disease while others do not impact this risk? Where and from whom?
- How would knowing they are at increased risk for heart disease affect your patients' health behavior (e.g., smoking, diet, exercise, taking medications, seeing/talking with your doctor).

Prescribing provider/pharmacist ONLY questions

- What helps you think about or better manage CVD in your HIV+ patients?
- Do you believe that your patients appropriately recognize their risk of heart disease? If your high-risk patients recognize their risk, are they then motivated to address risk factors and take preventive actions?
- What is your routine for monitoring cholesterol in your patients?
- How often do you review current or past cholesterol results for your patients? Prompts: every visit?, once yearly?, rarely?
- Blood pressure is typically measured at every clinical visit. Do you ever neglect to review the blood pressure because you are focused on other things? Do you ever notice the blood pressure is high but then decide that it is not a high enough priority to address at that visit?
- How comfortable are you with current guidelines about the definitions and management of high blood pressure?
- How comfortable do you feel prescribing hypertension and cholesterol medications and managing their side effects?
- How often do you prescribe home blood pressure monitoring? How do you follow up on adherence to these medications or monitoring? Prompts: Have your nurse call; patient calls in with numbers; check in with patient via email or EMR reminders.
- Do you talk with your patients about how HIV medications affect heart disease risk? If so, tell me about those conversations.
- Do you believe drug-drug interactions is a significant barrier to prescribing preventive medications like statins?
    - **HIV specialty providers**: If a non-HIV doctor prescribes a medication for your patients, do you know about it? How frequently do you encounter dangerous drug-drug interactions that result from others being unfamiliar with ART?
    - **PCPs (i.e. non-HIV provider)**: How knowledgeable are you about ART drug interactions? Do you avoid certain statin medications in all HIV patients for fear of drug interactions or do you check for specific interactions based on the patient's ART?
- Do you think your patients would be more likely to take a preventive medication (like aspirin or statins) if your HIV doctor prescribed it compared to a non-HIV provider? Why or why not?
- What types of patients do you refer to a specialist for cardiovascular disease <u>prevention</u>? Prompts: very high LDL or triglycerides (do you have a threshold)? Difficult to control hypertension (do you have a threshold—i.e. not controlled on 3 meds? 5 meds?), + family history?, patient preference/request?
- How comfortable are you ordering cardiovascular testing (i.e. stress tests, echocardiograms, holter/event monitors, coronary calcium scans)?

Nurse and Medical Assistant ONLY questions

- Can you tell me when in the patient visit your patients' blood pressure is assessed? What are the steps involved in measuring it? Is it logistically difficult for the patient to rest quietly for a time before it is taken? What makes this hard?
- How often do you reconcile your patients' medications? Do they ever have questions about their blood pressure and cholesterol medications? What sorts of questions do they ask? How do you address the questions?
- Do you assess medication adherence? For which medications? Do you provide feedback to the patient based on the medication adherence levels? What do you do if they are non-adherent? If they are non-adherent to any of their medications do you follow up with them after their visit to see if its changed?

Intervention Feasibly and Usability

Finally, we are interested in designing a new initiative, with a new nurse coordinator, to help your patients reduce their risk of heart disease. However, we know in this and many clinics, there are cultural and systemic issues that can make a new clinic initiative more or less successful. We'd like to get your opinion on these issues and as a reminder, all information you provide will be confidential and aggregated across three clinics.

System

- Tell me about your clinic's culture. What aspects of it help you do your job well and what aspects can make it harder for you? What are the strengths of your clinic?
- Does your clinic have a hierarchy? If so, can you tell me how that affects how decisions are made in your clinic?
- What the priorities of your clinic? Have they change over time?
- Tell me about a clinic initiative that was recently developed to help your patients improve their health behavior. What steps were taken to help make the change? Who initiated the change and how did having that person champion it affect the initiative? How long did it last? What was the outcome (did it work)?

Training

- What sort of education or training do you prefer for new initiatives? Do you prefer paper, verbal (conversations), digital (phone, computer) or visual (TV) training? Individual or Group? Does continuing education credit entice you to complete new trainings? Would you be interested in helping to develop a new training for your clinic on reducing heart disease in your patients?

Design Considerations/Representations

- When you have a patient concern, for example an elevated temperature or a high blood pressure, how does that information get turned into action in this clinic? Can you walk me through the various steps? [consider drawing this out on white board]. How do you involve non-HIV clinicians, for example your patient's primary care provider, in this communication cycle?
- How often do you personally communicate with your patients outside of the clinic setting? How does this communication happen (e.g., phone, EMR, via your nurse). Does the patient usually initiate this contact or do you?

Secondary/Follow-up Questions

Follow-up "Probes" after significant statements are made:

[Earlier/A moment ago/when you first started speaking/when you were talking about x] you said [significant statement].

- Can you tell me more about that?
- Can you tell me more about how that affects [X]
- Can you clarify what you mean by [significant statement]
- Can you give me an example of a time when [significant statement] happened to you

Conclusion

Is there anything else you want us to know about [X]? (YES return to interview; NO proceed)

We want to thank you so much for your participation and remind you that everything we have discussed will remain private. The audio file will be destroyed once this interview is transcribed, and the transcription will not contain your name or any identifying information.

COVID AIM: Key Informant Interview Guide for Patients Living with HIV

Introduction

[Remind about Audio Recording and not to use real names]

Thank you for talking with us today. We are interested in learning more about your thoughts and beliefs about how the coronavirus pandemic (or COVID-19) has impacted your cardiovascular health. I am going to ask you some questions about these topics to hear about your experiences and perspectives. Please know that there is no right or wrong answer. You will notice that I won't give you a lot of feedback on your responses because I don't want to influence your answers. You may also notice that I will jot things down on paper while you talk- this is simply a reminder to ask you a follow-up question. Finally, you are under no obligation to talk about anything that you are not comfortable discussing with me. Do you have any questions or concerns before we begin?

Today's date is: **and I am interviewing participant** [use study ID]

Primary Questions

General COVID-19 Perceptions

- Let's start by you telling me about what you know about the novel coronavirus. **Prompts**: What is it; how do you prevent it; how do you treat it if you get it; how do you know if you have it?
- Do you think you are at risk for becoming infected with coronavirus? Why? **Prompts**: Living situation, work, health care appointments, lack of social/material support?
- Have any of your doctors or nurses ever talked with you about coronavirus? If yes, what did they tell you? Have you ever asked a doctor or nurse about how your HIV may impact your likelihood of becoming sick with coronavirus? What about your other risk factors such as heart disease or high blood pressure?
- Have you ever been evaluated for coronavirus by a health care provider? If so, what did he or she do? Was it your HIV doctor or another doctor? What led to that evaluation (e.g., symptoms (chest pain, fever, etc…)

Isolation

- Tell me about your social interactions since word of coronavirus virus spread in March 2020. **Prompts**: How often do you go outside of your house? Where do you go? How do you get there (e.g., walking, driving, public transportation, bicycle?)
- Has this behavior changed over time (or X months since March 2020)? Why or Why not?

- Who do you talk to regularly on the phone or via computer? How has that changed over time? Who do you feel close to today?
    - Tell me about times when you have felt supported over the past X months?
    - Tell me about times when you have felt alone or isolated? How has that affected your mood? Your health? Your motivation to take of your health?

<u>COVID and Self-Management</u>

Let’s talk a bit more about how any changes you have made to your behavior in response to COVID pandemic may have influenced your health.

- What medications do you take for HIV? What about your heart health?
- How has coronavirus affected your ability to take your medications? **Prompts**: Your schedule is different; motivation changed/diminished; unable to get refills/see your health care provider? You can’t travel to get your medications?
- What else do you do to help manage your health? Physical activity, support groups, diet control, regularly clinic appointments, volunteer, read, take daily blood pressure measurements, etc?
    - How has this been impacted by the coronavirus?
- Do you think the spread of coronavirus will impact your ability to take care of your HIV and heart disease over the long-term? How or why not?
- Do you think it will impact your ability or willingness to visit your health care team?
- What are your experiences with telehealth or talking to health care team over the phone or computer? **Prompts**: How comfortable are you? Does it change your relationship with the team?
- What about doing more research visits on the computer or over the phone. Would you be willing to do that? What challenges do think you might encounter if you did more research visits over the phone or computer? What benefits do you think might occur with more virtual research visits?
- If we were change EXTRA-CVD to include more virtual visits/calls, what should we keep in mind? Prompts: Internet access? Ability to navigate platforms? Should we have an orientation visit first to make sure you understand the technology?
- How about the social support you receive form the research team. How could that be effected by fewer in person visits?
- Do you think more virtual visits would impact your willingness to participate in EXTRA-CVD and other research studies? If so, how could we offset that?

Secondary/Follow-up Questions

Follow-up “Probes” after significant statements are made:

[Earlier/A moment ago/when you first started speaking/when you were talking about x] you said [significant statement].

- Can you tell me more about that?
- Can you tell me more about how that affects [X]
- Can you clarify what you mean by [significant statement]
- Can you give me an example of a time when [significant statement] happened to you

Conclusion

Is there anything else you want us to know about [X]? (YES return to interview; NO proceed)

We want to thank you so much for your participation and remind you that everything we have discussed will remain private. The audio file will be destroyed once this interview is transcribed, and the transcription will not contain your name or any identifying information.

Appendix D: Clinic Variables Checklist

EXTRA-CVD

Clinic Variables Checklist

Date: ______________________________

Study site (circle one): UH/SIU MetroHealth Duke

Instructions: For each of the following support services, please circle the option that best describes availability of that services in your ID or HIV clinic.

Resources	Not Available	Same Day Availability	Available by Advance Scheduling/Request Only
Adherence Support Services (Ex. Assisted pill box filling, DOTS, etc.)	0	1	2
CVD Prevention Specialist/Consultant with HIV Expertise	0	1	2
Dietician/Nutritionist	0	1	2
Exercise or Fitness Classes	0	1	2
Medication Adherence Incentive Programs	0	1	2
Nurse Support Line	0	1	2
Peer Coaching	0	1	2
Pharmacist	0	1	2
Social Worker Led Support Group(s)	0	1	2
Tobacco Cessation Program	0	1	2
Transportation Vouchers	0	1	2

What additional in-clinic resources are available to aid patients in managing their hypertension and/or hyperlipidemia?

____

____

Appendix E: PLHIV Pilot Participant Acceptability Testing Guide

Note: Not all questions will be asked explicitly. Participants may answer the questions, or cover the topic, in the course of the one-on-one conversation.

Study background

Welcome to our discussion! People living with HIV (PLHIV) are at increased risk for heart disease; however, the uptake of evidence based therapies to prevent heart disease is substandard. As such, strategies to improve the uptake of cardiovascular disease preventive therapies among PLHIVs are urgently needed. The goal of this study is to test a prevention, nurse-led intervention to extend the HIV/AIDS treatment cascade for the treatment of hypertension and high cholesterol among PLHIV on suppressive antiretroviral therapy.

Introduction

[Remind about Audio Recording and not to use real names]

Thank you for talking with me today. I am interested in learning more about your thoughts and beliefs about cardiovascular disease among your HIV+ patients. I am most interested in your thoughts about a new intervention our research team is planning on scaling-up at your clinic and for your HIV+ patients who have hypertension and high cholesterol. I am going to ask you some questions about these topics to hear about your experiences and perspectives. Please know that there is no right or wrong answer.

Here are the ground rules for our focus group discussion. We would like to have everyone participate in this discussion. I will only give you all a few probes and would like for you all to react to those probes. Beyond giving the probes, the discussion will be entirely driven by you all – my only job is to facilitate. And I will facilitate to get more clarity on statements you all share. Additionally, I am interested in engaging each of you to talk freely and to make sure everyone has equal opportunities to speak. My main purpose here is to ask questions, listen, and make sure that you have the opportunity to share your thoughts and perspectives. This session is being recorded, as was noted in the consent form you signed before, so that none of your informative comments and feedback will be missed. Again your names will not be collected and your comments will be confidential. Finally, you are under no obligation to talk about anything that you are not comfortable discussing with me. Do you have any questions or concerns before we begin?

[Facilitator, please note the participant's ID number in the audio recording so we can match it to the demographic information.]

Intervention Acceptability

Finally, we are interested in a new initiative, with a new nurse coordinator - led intervention, that we designed to help patients like you reduce their risk of heart disease in clinics around the

country. As such we would like to get your opinion on thoughts on this intervention. As a reminder, all information you provide will be confidential and aggregated across three clinics.

Facilitators and barriers to the nurse-led intervention for HIV+ patients with hypertension and high cholesterol

1. Please tell me your thoughts on working with your CVD nurse to tailor treatment for HIV+ patients like yourself who have either been diagnosed with hypertension and high cholesterol.
    a. PROBE: What general experience do you have working with nurses to manage your health?
2. What do you think are some potential benefits or barriers of working with nurses?

[Interviewer will share the refined intervention structure and format with the participant(s).]

Thoughts on logistical factors (i.e. resources or tools at that clinic)

3. What are your initial thoughts on this intervention?
4. Do you like this intervention?
5. Please tell me any potential concerns you may have as an HIV+ patient participating in this intervention?
6. What are some potential benefits that you see with integrating this intervention into your current treatment?
7. What are some current resources or tools within your clinic that you think will help facilitate the integration of this intervention for your care?
    a. Conversely, what may be some aspects of your clinic that you think will be a barrier to integrating this intervention for your care or treatment?
8. Do you feel that this intervention is relevant in terms of your health or the issues you are dealing with?
9. Would you be interested in having this service offered to you as part of your treatment?

Secondary/Follow-up Questions
Follow-up "Probes" after significant statements are made:
[Earlier/A moment ago/when you first started speaking/when you were talking about x] you said [significant statement].

- Can you tell me more about that?
- Can you tell me more about how that affects [X]
- Can you clarify what you mean by [significant statement]
- Can you give me an example of a time when [significant statement] happened to you

Conclusion

Is there anything else you want us to know about [X]? (YES return to interview; NO proceed)

We want to thank you so much for your participation and remind you that everything we have discussed will remain private. The audio file will be destroyed once this interview is transcribed, and the transcription will not contain your name or any identifying information.

Appendix F: PLHIV Pilot Participant Feasibility Testing Guide

Note: Not all questions will be asked explicitly. Participants may answer the questions, or cover the topic, in the course of the one-on-one conversation.

Study background

Welcome to our discussion! People living with HIV (PLHIV) are at increased risk of heart disease; however, the use of the most effective treatment options or approaches to manage heart disease in this population is not the best. As such, strategies to improve the uptake of heart disease prevention among PLHIVs is very important and needed in HIV clinics. The goal of this study is to test a prevention, nurse-led intervention to extend the HIV/AIDS treatment cascade for the treatment of hypertension and high cholesterol among PLHIV on suppressive antiretroviral therapy.

Introduction

[Remind about Audio Recording and not to use real names]

Thank you for talking with me today. I am interested in learning more about your thoughts and beliefs about cardiovascular disease among your HIV+ patients. I am most interested in your thoughts about a new intervention our research team is planning on scaling-up at your clinic and for your HIV+ patients who have hypertension and high cholesterol. I am going to ask you some questions about these topics to hear about your experiences and perspectives. Please know that there is no right or wrong answer.

Here are the ground rules for our focus group discussion. We would like to have everyone participate in this discussion. I will only give you all a few probes and would like for you all to react to those probes. Beyond giving the probes, the discussion will be entirely driven by you all – my only job is to facilitate. And I will facilitate to get more clarity on statements you all share. Additionally, I am interested in engaging each of you to talk freely and to make sure everyone has equal opportunities to speak. My main purpose here is to ask questions, listen, and make sure that you have the opportunity to share your thoughts and perspectives. This session is being recorded, as was noted in the consent form you signed before, so that none of your informative comments and feedback will be missed. Again your names will not be collected and your comments will be confidential. Finally, you are under no obligation to talk about anything that you are not comfortable discussing with me. Do you have any questions or concerns before we begin?

[Facilitator, please note the participant's ID number in the audio recording so we can match it to the demographic information.]

Intervention Feasibility

We are interested your experience and thoughts have participated in the pilot test for the new nurse coordinator-led intervention that we designed to help your patients reduce their risk of heart disease in clinics around the country. As a reminder, all information you provide will be confidential and aggregated across three clinics.

Participating in the nurse-led intervention for HIV+ patients with hypertension and high cholesterol:

1. Please tell me what your general thoughts and experience was with the nurse-led intervention?
2. What effect (positive or otherwise) did the intervention have on how you engaged with the following providers:
    a. Prevention nurse: How did you interactions with the nurse evolve over the course of the 6-week pilot?
    b. Your primary HIV nurse: Did your participation in this pilot affect your relationship with your primary HIV nurse? If so, how?
    c. Your physician providers: How did your participation in this pilot affect your relationship with your primary HIV doctor and/or your primary care doctor?
3. After meeting with your nurse during the intervention what differences did you notice in how you responded to your own treatment?
    a. PROBE: How where you better able to treat or care for your hypertension and cholesterol?

Thoughts on intervention's feasibility

4. How do you feel this intervention fits within your clinic setting?
5. After completing all of the intervention-related meetings with the nurse, what barriers did you feel that they faced in helping you manage your hypertension and cholesterol?
6. In what ways do you think this intervention, in its current form, will be an effective tool or resource for your clinic to integrate going forward?
7. In what ways do you think this intervention, in its current form, will NOT be an effective tool or resource for your clinic to integrate going forward?
8. What feedback have you shared or provided about the intervention with the clinic supervisors or nurses?
9. Does this intervention seem possible as a form of HIV treatment or management for people with hypertension and high cholesterol?
10. How do you think this intervention can be implemented within general HIV care clinics?

Secondary/Follow-up Questions

Follow-up "Probes" after significant statements are made:

[Earlier/A moment ago/when you first started speaking/when you were talking about x] you said [significant statement].

- Can you tell me more about that?
- Can you tell me more about how that affects [X]
- Can you clarify what you mean by [significant statement]
- Can you give me an example of a time when [significant statement] happened to you

Conclusion

Is there anything else you want us to know about [X]? (YES return to interview; NO proceed)

We want to thank you so much for your participation and remind you that everything we have discussed will remain private. The audio file will be destroyed once this interview is transcribed, and the transcription will not contain your name or any identifying information.

Appendix G: Health Worker Feasibility Testing Guide

Note: Not all questions will be asked explicitly. Participants may answer the questions, or cover the topic, in the course of the one-on-one conversation.

Study background
Welcome to our discussion! People living with HIV (PLHIV) are at increased risk for heart disease; however, the uptake of evidence based therapies to prevent heart disease is substandard. As such, strategies to improve the uptake of cardiovascular disease preventive therapies among PLHIVs are urgently needed. The goal of this study is to test a prevention, nurse-led intervention to extend the HIV/AIDS treatment cascade for the treatment of hypertension and high cholesterol among PLHIV on suppressive antiretroviral therapy.

Introduction
[Remind about Audio Recording and not to use real names]
Thank you for talking with me today. I am interested in learning more about your thoughts and beliefs about cardiovascular disease among your HIV+ patients. I am most interested in your thoughts about a new intervention our research team is planning on scaling-up at your clinic and for your HIV+ patients who either have or at risk for CVD. I am going to ask you some questions about these topics to hear about your experiences and perspectives. Please know that there is no right or wrong answer.

Here are the ground rules for this semi-structured interview. I am going to ask you a few questions and will only give you a few probes with the intention that you will react to those probes. Beyond giving the probes, the discussion will be entirely driven by your responses – my only job is to facilitate. And I will facilitate to get more clarity on statements you share. Additionally, I am interested in engaging each you to talk freely and to make sure you are able to share your thoughts fully. My main purpose here is to ask questions, listen, and make sure that you have the opportunity to share your thoughts and perspectives. This session is being recorded, as was noted in the consent form you signed before, so that none of your informative comments and feedback will be missed. Again your name will not be collected and your comments will be confidential. Finally, you are under no obligation to talk about anything that you are not comfortable discussing with me. Do you have any questions or concerns before we begin?

[Facilitator, please note the participant's ID number in the audio recording so we can match it to the demographic information.]
Intervention Feasibility
We are interested your experience and thoughts have participated in the pilot test for the new nurse coordinator-led intervention that we designed to help your patients reduce their risk of heart disease in clinics around the country. As a reminder, all information you provide will be confidential and aggregated across three clinics.

Participating in the nurse-led intervention for HIV+ patients with hypertension and cholesterol
1. Please tell me about your thoughts and experience with the nurse-led intervention?
2. How did the intervention impact (positive or otherwise) the way in which you engaged with HIV+ patients?

3. Did you feel prepared to integrate the intervention in your current treatment routine? If so, how? If not, what would have helped you to be more prepared?
4. After meeting with your patients during the intervention, what were some differences you noticed in how patients responded to their treatment?
    a. PROBE: How where patients better able to treat or care for their CVD risks?

Thoughts on the intervention's feasibility

5. How do you feel this intervention fits within your clinic setting?
6. After completing all of the intervention procedures with your patients, did you feel that they still faced any barriers to managing their hypertension and cholesterol?
7. How did this intervention impact your workload with treating HIV+ patients?
8. In what ways do you think this intervention, in its current form, will be an effective tool or resource for your clinic to integrate going forward? In other words, what worked well in this intervention in this particular setting?
9. In what ways do you think this intervention, in its current form, will NOT be an effective tool or resource for your clinic to integrate going forward? Or what can be improved?
10. What feedback have you shared or provided about the intervention with your clinic colleagues?
11. Does this intervention seem possible as a form of HIV treatment or management for people with hypertension and high cholesterol?
12. How do you think this intervention can be implemented within general HIV care clinics?

Secondary/Follow-up Questions

Follow-up "Probes" after significant statements are made:
[Earlier/A moment ago/when you first started speaking/when you were talking about x] you said [significant statement].

- Can you tell me more about that?
- Can you tell me more about how that affects [X]
- Can you clarify what you mean by [significant statement]
- Can you give me an example of a time when [significant statement] happened to you

Conclusion

Is there anything else you want us to know about [X]? (YES return to interview; NO proceed)
We want to thank you so much for your participation and remind you that everything we have discussed will remain private. The audio file will be destroyed once this interview is transcribed, and the transcription will not contain your name or any identifying information.

Appendix H: Design Team Discussion Guide

Themes to be assessed:

- Previous design team experience.
- Understanding of the design team approach.
- Thoughts on the design team process for the nurse-led intervention.
- Experience for each phase or meeting of the design approach.
- Thoughts on the final intervention model.

General focus group discussion introduction:

For this part of our design team meeting, I am most interested in learning more about your thoughts and beliefs about the human-centered design process. I am going to ask you some questions about the process to hear about your experiences and perspectives. Please know that there is no right or wrong answer.

Here are the ground rules for our focus group discussion. We would like to have everyone participate in this discussion. I will only give you all a few probes and would like for you all to react to those probes. Beyond giving the probes, the discussion will be entirely driven by you all – my only job is to facilitate. And I will facilitate to get more clarity on statements you all share. Additionally, I am interested in engaging each of you to talk freely and to make sure everyone has equal opportunities to speak. My main purpose here is to ask questions, listen, and make sure that you have the opportunity to share your thoughts and perspectives. This session is being recorded, as was noted in the consent form you signed before, so that none of your informative comments and feedback will be missed. Again your names will not be collected and your comments will be kept confidential, not to be revealed to anyone outside of this room. Finally, you are under no obligation to talk about anything that you are not comfortable discussing with me. Do you have any questions or concerns before we begin?

Focus group questions

- What impact do you think your individual participation in the design team had on the final intervention model?
- Tell me about your experiences working on a team with individuals from different professions and roles?
- What was your personal contribution and the overall team's contribution to the design team?
- Was there a particular design phase (i.e. brainstorming, conceptualization, creation, iteration I, or iteration II) that you found to be most insightful for the entire design experience? Why or why not?
- Which design phase (i.e. brainstorming, conceptualization, creation, iteration I, or iteration II) did you find to be most challenging and why do you think so?
- Which design phase (i.e. brainstorming, conceptualization, creation, iteration I, or iteration II) did you find to be least challenging and why do you think so?

- What are your general thoughts on the final intervention model? Do you think that this version will meet the intended goals of our design team and that of the research study?
- Are there any closing thoughts that you want to share or that you think we should have discussed in terms of the final intervention model that we do not touch on?
- Are there any final thoughts that you want to share or that you think we should have discussed in terms of the design team experience that we do not touch on?

Thank you for your time and participation. We very much appreciate your comments, discussions, and input throughout the design process.

Appendix I: Trust and Communication Survey (PLHIV participant – Provider)

As a participant in the EXTRA-CVD study, we are asking you to complete a survey about your relationship with your healthcare providers. You will need at least 15 minutes of uninterrupted time to take this survey. If you have any questions, please see your EXTRA-CVD nurse or study coordinator. Please understand that your answers will be confidential. More specifically, your name and the names of your providers will never be recorded with your data. The unique ID numbers will be kept separately by study staff at your site who do not have access to the survey results. Please respond below in order to proceed:

- O Yes I understand my rights as a participant in this study. I consent to proceed.
- O No I do not consent and will not participate in this study and survey

Please enter here your unique participant ID. EXTRA-CVD study staff will ensure that you enter your correct ID. PLEASE DO NOT LIST YOUR NAME.

____

Please select which visit you completed today as part of this study. EXTRA-CVD study staff will ensure that you select the correct answer.

- O Baseline visit
- O 4 months visit
- O 8 months visit
- O 12 months visit

As a participant of this study, in the following sections, you will be asked some questions regarding your relationships with providers that may help treat your HIV or your blood pressure and cholesterol. At a minimum, this will include: your HIV provider and your EXTRA-CVD nurse.

Additionally, you may be asked some questions about the rest of your health care providers (for example, your Primary care physician, your HIV clinic nurse or you Non-HIV specialists).

We hope to collect quality data for our research. But If you feel uncomfortable to answer any of the questions or feel that a question if not applicable to you, feel free to skip it.
Do you have a HIV clinic nurse?

O Yes

O No

Do you have a Primary care physician?

O Yes

O No

Please indicate which of the following medical professionals primarily treats your blood pressure (if multiple physicians care equally for your blood pressure, please select all that apply):

☐ My HIV provider

☐ My HIV clinic nurse

☐ My Primary care physician

☐ My Non-HIV specialist, please specify the specialist type (i.e.; cardiologist, nephrologist, endocrinologist): ____

☐ No one manages my blood pressure

Please indicate which of the following medical professionals primarily treats your cholesterol (if multiple physicians care equally for your cholesterol, please select all that apply):

☐ My HIV provider

☐ My HIV clinic nurse

☐ My Primary care physician

☐ The same Non-HIV specialist who also treats my blood cholesterol (as indicated in the question above)

☐ A different Non-HIV specialist from whom treats my blood cholesterol as indicated in the prior question), please then specify the specialist type- i.e. cardiologist, nephrologist, endocrinologist): ____

☐ No one manages my cholesterol

*** The participant will complete the following survey items for each of their providers identified above.

Please think of your relationship with your [HIV, PCP, non-HIV specialist] provider. For the validity of this study, please answer honestly. Recall your answers are confidential and will never be shared with this provider nor anyone else outside our research team. Some of the questions may sound repetitive, please do read fully before answering each question.

How long have you been a patient of this provider?

O I just became a patient this past month

O Less than 6 months

O 6 months to 1 year

O 1-2 years

O 3-5 years

O More than 5 years

How often do you communicate with this provider?

O Every 1-2 months

O Every 3-4 months

O Every 5-6 months

O Once yearly

Please rate the following statements based on your recent visits with this provider and overall care with this provider.

	Strongly Disagree	Disagree	Neither Agree nor Disagree	Agree	Strongly Agree
My provider has much knowledge about the treatment that needs to be done.	O	O	O	O	O
My provider is very concerned about my welfare.	O	O	O	O	O
I feel I can discuss with my provider how I honestly feel about my health treatment, even negative feelings and frustration.	O	O	O	O	O
Overall, I trust my provider.	O	O	O	O	O

	Strongly Disagree	Disagree	Neither Agree nor Disagree	Agree	Strongly Agree
My provider and I communicate effectively.	O	O	O	O	O
My provider listens carefully to me.	O	O	O	O	O
Overall, I am satisfied with the communication with my provider.	O	O	O	O	O

.

	Strongly Disagree	Disagree	Neither Agree nor Disagree	Agree	Strongly Agree
My provider and I have a good relationship.	O	O	O	O	O
I feel part of a team with my provider in regards to my health care and needs.	O	O	O	O	O
Overall, I like my provider.	O	O	O	O	O
Overall, I am satisfied with my provider.	O	O	O	O	O

Do you have any recommendations to improve your care experience with this provider?

____

____

____

____

____

Appendix J: Trust and Communication Survey (Prevention RN – PLHIV participant)

Enter the participant ID

____

Please indicate the study visit:

- O Baseline visit (0)
- O 4 month visit (1)
- O 8 month visit (2)
- O 12 month visit (3)

Please think of your relationship with the patient you listed above. For the validity of this study, please answer honestly. Recall your answers are confidential and will never be shared with any provider, patient nor anyone else outside our research team. Some of the questions may sound repetitive, but please read fully before answering each question.

Please rate the following statements based on your recent interaction with this patient.

	Strongly Disagree (1)	Disagree (2)	Neither Agree nor Disagree (3)	Agree (4)	Strongly Agree (5)
Overall, I trust this patient.	O	O	O	O	O
Overall, I am satisfied with my communication with this patient	O	O	O	O	O
Overall, I have a good relationship with this patient.	O	O	O	O	O

Thank you! To rate another participant, please open a new survey.

Appendix K: Trust and Communication Survey (Prevention RN – Provider)

Please input only the unique identifier of the provider (do not enter any names!) that you will be rating in the next set of questions. Please verify this ID is correct before continuing.

____

Please rate the following statements based on your recent interactions (since the last survey) with this provider. Your responses to these questions may or may not change over time as you continue to interact with this provider over the course of the EXTRA-CVD intervention.

	Strongly Disagree	Disagree	Neither Agree nor Disagree	Agree	Strongly Agree
Overall, I trust this provider.	O	O	O	O	O
Overall, I am satisfied with the communication with this provider.	O	O	O	O	O
Overall, I have a good relationship with this provider.	O	O	O	O	O

Do you have another provider to rate?

O Yes

O No

Appendix L: Study staff and prevention nurse observation checklist to ensure intervention fidelity.

Study Staff Observation Checklist:

Date:

Observer:

Location:

Setup:

- ☐ Checked that Blood Pressure (BP) machines are functional and available
- ☐ Knew the directions to the blood lab and the working hours

Explain and correctly perform blood pressure procedures (study staff NOT prevention nurse):

- ☐ Participant was sitting with feet on the floor
- ☐ Participant's arm was at heart level
- ☐ Participant removed extra clothing (coat/jacket must be removed; long sleeve shirts may remain on)
- ☐ Participant was encouraged to relax
- ☐ Participant was not talking during measurement
    - o If participant was talking, measurement was repeated
- ☐ BP cuff was well placed above the elbow and admitting two fingers
- ☐ Correct size of BP cuff was used
- ☐ Two BPs were obtained, 1 minute apart
- ☐ Blood pressure was checked in both arms

Informed consent:

- ☐ Explained purpose of research study

- ☐ Explained description of procedure
- ☐ Explained confidentiality of study data
- ☐ Explained potential benefits
- ☐ Explained potential risks
- ☐ Explained voluntary participation and rights to discontinuation
- ☐ Checked understanding with open questions. “Could you tell me what’s going to happen if you enroll in the study?”)
- ☐ Checked understanding with closed questions (e.g. “Will you get free medications from the staff of this research study?” or “what will happen to your medical care after the study?”
- ☐ Corrected misconceptions (eg: Patient thinks they will get free medications or patient thinks they don’t have to follow up with their doctor after this study is done”) and recheck understanding.
- ☐ Filled the informed consent checklist
- ☐ Requested signature or participant thumbprint
- ☐ Asked if participant has any questions

<u>Data collection:</u>

- ☐ Explained how to use REDCap to the participant
- ☐ Appropriately gathered medical history data from chart review
- ☐ Appropriately answered questions about how to respond to individual survey questions

Prevention nurse procedures checklist:

Date:

Observer:

Location:

Establishing Rapport:

- ☐ Introduced herself/ himself to the patient
- ☐ Made eye contact with the patient
- ☐ Listened to the patient
- ☐ Was able to engage patient in the conversation

Counselling: Baseline

- ☐ Asked patient for the purpose and side effects of his/her current medications
- ☐ If a new medication is added, PN discussed purpose and side effects of the medication, and any required follow up
- ☐ Discussed strategies for medication adherence
- ☐ Discussed non pharmacological targets for blood pressure control
- ☐ Asked patient for their preferred primary provider
- ☐ Discussed methods of home BP monitoring
- ☐ Set expectations with the patient about follow up phone calls and visits
- ☐ Informed primary and non primary providers of plan at the end of visit

Counselling: Follow up

- ☐ Asked patient for home BP values over the last 2 weeks
- ☐ Checked patient's understanding of the method of home BP measurement.
- ☐ Reviewed patient's medication list and adherence
- ☐ If patient is non- adherent, prevention nurse discussed reasons for non-adherence (Eg. side effects, cost, timing etc.)

- ☐ If a new medication is added, prevention nurse discussed purpose and side effects of the medication, and any required follow up
- ☐ Referred patient to the blood lab for a lipid panel
- ☐ Set expectations for follow up phone call and visits
- ☐ Informed primary provider and non primary provider of updated plan

Appendix M: Behavior Change Counseling Index (BECCI) to Measure Practitioner's Motivational Interviewing Skills

**Date:**<br>**Staff and Role:**<br>**Observer:**	**Strengths:**<br>**Area for Growth:**<br>**Scoring: 0** = Not at all, **1** = Minimally, **2** = To some extent, **3** = A good deal, **4** = A great extent, or **N/A**

Item	Description	Score	N/A	Comments
**1.** Invites the person to talk about behavior change*	Explicitly asks permission to talk about behavior change. Conveys person is not obligated to make a decision.			•
**2.** Demonstrates sensitivity to talking about other issues	Person is given choice in what to talk about; use of agenda setting.			•
**3.** Encourages person to talk about current behavior/status quo	Encourages person to talk freely about the benefits and limitations of current behavior. Uses open-ended questions to elicit person's perspective and reflections to convey understanding.			•
**4.** Encourages person to talk about change	Encourages person to talk freely about the benefits and limitations of making a behavior change. Uses of open-ended questions to elicit person's perspective and reflections to convey understanding.			•
**5.** Asks questions to elicit how person thinks and feels about topic	Uses open-ended questions to elicit person's thoughts and feelings related to a behavior change			[Closed Questions – CQ]<br>•<br>[Open Questions – OQ]<br>•

6. Uses reflective listening when the person talks about the topic	Uses reflections to convey understanding, clarify understanding of what the person has said and encourage further patient elaboration.			•
**7.** Uses summaries to bring together what the person says about the topic	Uses periodic summaries (collecting, linking, transitional) to check understanding, assists person to hear what they have said and/or transition to a new task (i.e., change planning)			•
**8.** Acknowledge challenges about behavior change that the person faces	Uses reflections to convey understanding that change is difficult and affirm the person's strengths			•
**9.** When providing information it is sensitive to person's concerns and understanding*	Attempts to understand what the person knows, wants to know and elicits their understanding of information provided and/or reaction to information provided. N/A if no information requested or shared			•
**10.** Staff and person exchange ideas about how the person could change current behavior*	Uses open-ended questions to encourage person to brainstorm strategies that support behavior change; person offers most of the ideas; permission to disregard and multiple ideas are offered when staff makes suggestions. N/A if person is not ready to plan for change			•
**BECCI Overall (Average) Score**				
**11.** Staff speaks for approximately	• More than half the time<br>• About half the time<br>• Less than half the time	•		

MI Skill Development Plan

Name: Date:

Strengths Demonstrated in Session		
**Skill Development**		
**MI Skill Targeted for Improvement**	**What specifically will be developed or improved?**	**How will the goal be reached?**

Appendix O. Functional Assessment of Currently Employed Technology Scale (FACETS), Copyright 2018 Charles M. Lepkowsky, Ph.D

A.	Home Domain						
1.	I send email...	Never	A few times a year	A few times a month	Once a week	A few times a week	Daily
2.	I find, open & close files in my computer...	Never	A few times a year	A few times a month	Once a week	A few times a week	Daily
	Home Domain Subtotal						
B.	Social Domain						
3.	I send text messages using a smart phone…	Never	A few times a year	A few times a month	Once a week	A few times a week	Daily
4.	I post on social media (e.g., facebook, twitter)…	Never	A few times a year	A few times a month	Once a week	A few times a week	Daily
	Social Domain Subtotal						
C.	E-Commerce Domain						
5.	I manage my banking and credit card accounts online…	Never	Tried, but it didn't work	Got help but didn't work	Only with help	Can but prefer not to	Prefer to
6.	I pay bills and make purchases via the internet…	Never	Tried, but it didn't work	Got help but didn't work	Only with help	Can but prefer not to	Prefer to
	E-Commerce Domain Subtotal						
D.	Health Care Domain						
7.	I communicate with my doctor or clinic online…	Never	Tried, but it didn't work	Got help but didn't work	Only with help	Can but prefer not to	Prefer to
8.	I communicate with my health insurance company online…	Never	Tried, but it didn't work	Got help but didn't work	Only with help	Can but prefer not to	Prefer to
	Health Care Domain Subtotal						
E.	Technical Domain						
9.	I have installed components (monitors, speakers, mice)…	Never	Tried, but it didn't work	Got help but didn't work	Only with help	Myself, with difficulty	Myself easily
10.	I have reset a modem or router in my home…	Never	Tried, but it didn't work	Got help but didn't work	Only with help	Myself, with difficulty	Myself easily
	Technical Domain Subtotal						
	**Total FACETS Score**						

Appendix P. Virtual Communication Preferences Form

Person Completing this Form:
Name ____
Role ____

Basic Information
First Name: ________________________________Enrollment Date: ___________________
Preferred Name: ____________________________MRN: _____________________________
Last Name: ________________________________Study ID: ___________________________

Contact Information

a) Which of the following would you like us to use to get in touch with you (circle all that apply)?
   i. *Phone* *Text* *E-mail*

b) What is the best telephone number to reach you?
(___ ___ ___) ___ ___ ___ - ___ ___ ___ ___

c) Is there a next best phone number to reach you?
(___ ___ ___) ___ ___ ___ - ___ ___ ___ ___

d) What days of the week are best for us to reach you? ____________________________

e) What is the best time of the day to reach you? __________________________________

f) Will you be the only person answering these phone numbers? If not, how do you want us to identify ourselves?

g) Please rate your skills in this area by selecting the most appropriate answer for each statement.

Can you:

	I'm not sure what this means	I haven't done this	I did this once	I do this regularly	I could teach someone else how to do this
**Email**					
Send an email to one or more people?	¨	¨	¨	¨	¨
Open and/or save an attachment from a received email?	¨	¨	¨	¨	¨
**Text**					
Open a text message from someone on your phone	¨	¨	¨	¨	¨

Send a text message on your phone to one or more people	¨	¨	¨	¨	¨
**Browsing the internet**					
Open a website link	¨	¨	¨	¨	¨
Copy and paste a website link	¨	¨	¨	¨	¨
**FaceTime**					
Accept or send a FaceTime request to communicate with people on video	¨	¨	¨	¨	¨
**Zoom Video Conference**					
Open a zoom meeting invite from your email	¨	¨	¨	¨	¨
Communicate with a health care provider on zoom	¨	¨	¨	¨	¨

h. How comfortable are you with communicating about your health on zoom/FaceTime or other video platform? (please circle one)

1 2 3 4 5 6 7 8 9 10

Not comfortable at all Very Comfortable

i. How concerned would you be about your living space when communicating with a provider on video? (please circle one)

1 2 3 4 5 6 7 8 9 10

Not comfortable at all Very Comfortable

j. How concerned would you be about confidentiality or being overheard when communicating with a provider on video? (please circle one)

1 2 3 4 5 6 7 8 9 10

Not comfortable at all Very Comfortable

k. Please rank your preference for communication with the EXTRA-CVD nurse (1 being the most preferred choice):

Telephone (no video): ________
Zoom: ________
FaceTime: ________
Other: ________

k. Do you have concerns about communicating with the EXTRA-CVD intervention nurse on any of the platforms mentioned above? Please explain.

APPENDIX Q: AAIM-High Observation Checklist

Date:
Observer:
Location:

What was the platform used by the nurse and participant for the observed study visit?

- o Zoom
- o FaceTime
- o Telephone visit
- o In-person consent
- o Other: ___________

What type of study visits was observed?

- o Consent visit
- o Baseline visit
- o 4-month follow-up visit
- o 8-month follow-up visit
- o Final 12-month visit
- o Interim check-in (not a quarterly follow-up visit)
- o Other: ___________

Did the observer observe in real time or observe to a recorded session?

- o Live session
- o Recorded

(If Live) Live setup:

- o Observer and nurse join Zoom room or telephone call prior to participant visit
- o Prevention nurse introduces observer (if applicable) and purpose

(If Recorded) Recorded session:

- o Prevention nurse obtains verbal consent prior to recording
- o Prevention nurse begins recording session (use tape recorder if telephone visit and Zoom recording feature if Zoom visit)

Data collection (All but consent visit).

Ensure participant correctly performs a home BP measurement (check all that apply):

- o Participant was sitting with feet on the floor
- o Participant's arm was at heart level
- o Participant removed extra clothing (coat/jacket must be removed; long sleeve shirts may remain on)
- o Participant relaxed for five minutes
- o Participant was not talking during measurement
- o If participant was talking, prevention nurse encouraged participant to repeat measurement
- o BP cuff was well placed above the elbow and admitting two fingers

Did the prevention nurse provide any coaching to the patient regarding blood pressure procedures?

- o Yes (Explain: ______________________)
- o No

Data collection:

- o Appropriately gathered medical history data from chart review

Is the participant completing surveys independently using REDCap?

- o Yes
- o No

If yes:

- o Explained how to use REDCap to the participant
- o Appropriately answered questions about how to respond to individual survey questions

If no:

- o Read questions to the participant verbatim
- o Appropriately answered questions about how to respond to individual survey questions

Counseling (all but consent visit)

Establishing Rapport:

- Made an effort to affirm the patient virtually by nodding, active listening, etc.
- Listened to the patient
- Was able to engage patient in the conversation

Counselling: Baseline

- Asked patient for the purpose and side effects of current medications
- If a new medication is added, PN discussed purpose and side effects of the medication, and any required follow up
- Discussed strategies for medication adherence
- Discussed non pharmacological targets for blood pressure control
- Asked patient for their preferred primary provider
- Discussed methods of home BP monitoring
- Assessed comfort and provided coaching on technology
- Provided troubleshooting for technology issues
- Set expectations with the patient about follow up phone calls and visits
- Informed primary and non primary providers of plan at the end of visit

Counselling: Follow up

- Asked patient for home BP values over the last 2 weeks
- Checked patient's understanding of the method of home BP measurement.
- Reviewed patient's medication list and adherence
- If patient is non- adherent, prevention nurse discussed reasons for non-adherence (Eg. side effects, cost, timing etc.)
- If a new medication is added, prevention nurse discussed purpose and side effects of the medication, and any required follow up
- Assessed comfort and provided coaching on technology
- Provided troubleshooting for technology issues
- Set expectations for follow up phone call and visits
- Informed primary provider and non primary provider of updated plan

Consent Visit (consent visit only)

Communication Preferences:

- o Appropriately obtains communication preferences and engages in conversation about technology

Informed consent:

- o Explained purpose of research study
- o Explained description of procedures
- o Explained confidentiality of study data
- o Explained potential benefits
- o Explained potential risks
- o Explained voluntary participation and rights to discontinuation
- o Checked understanding with open questions. ("Tell me what's going to happen if you enroll in the study.")
- o Checked understanding with closed questions (e.g. "Will you get free medications from the staff of this research study?" or "what will happen to your medical care after the study?"
- o Corrected misconceptions (eg: Patient thinks they will get free medications or patient thinks they don't have to follow up with their doctor after this study is done") and recheck understanding.
- o Filled the informed consent checklist
- o Requested signature
- o Asked if participant has any questions

Materials and supplies:

- o BP cuff
- o Patient has the cuff already (EXTRA-CVD Intervention Arm participant)
- o Patient is already familiar with the device-- no need to demonstrate
- o Patient not familiar-- device taken out of the box and demonstrated
- o Living a Healthy Life Book
- o If not checked, why not?
- o Pt declined
- o Staff forgot or didn't have to give out
- o BP log sheets
- o QR code sheet

Appendix R: AAIM High clinic context form

AAIM-High

Clinic Variables Checklist

Date: ______________________________

Study site (circle one): UH/SIU MetroHealth Duke

Which of the following potential barriers to providing telemedicine care has your clinic experienced during the pandemic?

	Not a barrier		Modest Barrier		Significant Barrier
Patient access to hardware (smartphone, laptop w/camera, etc…)	1	2	3	4	5
Patient tech literacy	1	2	3	4	5
Patient access to wireless or cellular data	1	2	3	4	5
Administrative pressure to do more in-person visits	1	2	3	4	5
Provider tech literacy	1	2	3	4	5
Provider discomfort or lack of experience providing telemedicine	1	2	3	4	5
Inappropriate physical environment to conduct virtual visits	1	2	3	4	5

What platforms does your health system offer for "virtual" videoconference appointment with patients? Rank them in order of frequency of use (1 = most used)

- ☐ Doxy.me Rank: ________
- ☐ Zoom Rank: ________
- ☐ FaceTime Rank: ________
- ☐ Other: ________ Rank: ________
- ☐ Other: ________ Rank: ________

On average across all providers, what proportion of the clinic's total visits fell in each of the following categories during each of these periods of time (Each column must add to 100%):

	Mar - Jun 2020	Jul-Oct 2020	Nov 2020 - Feb2021	Mar 2021- Current
In-Person				
Telephone				

"Virtual" audio + visual				

Please describe how clinic providers have been educated about providing "virtual" care during the pandemic (paragraph notes):

____

____

Please discuss the clinic culture around providing virtual care with a focus on any characteristics you believe might be unique to your setting:

____

____

Is there anything else about your clinic setting that you believe may influence how the AAIM-High intervention will be implemented at your site:

____

____

Appendix S: Key Informant Interview Guides for PLHIV and Clinicians

Key Informant Interview Guide for Patients Living with HIV

Introduction

[Remind about Audio Recording and not to use real names]

Thank you for talking with us today. We are interested in your thoughts and beliefs about a recent blood pressure and cholesterol study with [study nurse] we tested in your HIV clinic. I am going to ask you some questions about the intervention to hear about your experiences and perspectives. Please know that there is no right or wrong answer. You will notice that I won't give you a lot of feedback on your responses because I don't want to influence your answers. Finally, you are under no obligation to talk about anything that you are not comfortable discussing with me. Do you have any questions or concerns before we begin?

Primary Questions

<u>Process Evaluation</u>

Context

- What medicines do you take for your cholesterol, blood pressure, or heart? How often do you take them?
- Prior to beginning this study, how often did you check your blood pressure at home? How have you incorporated checking your blood pressure into your daily routine?
- What else do you do to help prevent heart problems or lower your cholesterol or blood pressure? Who or what helps you do that?
- Tell me about the support you have to help take care of your health, like taking your medications, and checking your blood pressure. Is it hard to ask for help about your health?
- What makes it easier for you to take care of your health or prevent cardiovascular disease? What stands in the way of caring for your health?
- What sort of impact does your relationship with your care team have on your health? Tell me about how your relationship with providers and how it has evolved over time.
- Compared to a year ago, tell me about your physical activity today. What about what you typically eat?
- Today, can you tell me how HIV may affect your risk for heart disease? If so, how?

<u>Intervention Dose and Fidelity</u>

- Did your blood pressure or cholesterol medication change during the study? If so, tell me what that was like for you.

- You recently participated in the intervention to reduce your risk of heart disease. How, if at all, has participating in the intervention changed the way you think about your risk for heart disease? Which of your health care team members talked with you about your risk for heart disease over the last year? What did he or she or they say? What role, if any, did HIV have in those conversations?
- You worked with a nurse who was supposed to help you coordinate the activities needed to reduce your risk of heart disease. This included calling you to remind you of your upcoming visits, checking in on your blood pressure monitor numbers, making sure the medications were working for you. Can you tell me the types of activities the nurse helped you with? [Probes: Which of those activities were most helpful for improving your heart health? What was it about that [activity] that was so helpful to you?]
- How often did you interact with the nurse? [Probes: How, if at all, did phone interactions with the nurse differ from your in-person interactions? Which type of interaction was more common? Which form of communication did you prefer?]
- Did the contacts with the nurse feel predictable or did you feel like you received something different from the nurse every time? Tell me more.
- What was your experience and feelings with the number of visits and phone calls you participated in for this study? Explain what you believe would be the ideal number of visits and calls if you were going to continue engaging in an intervention like this in the HIV clinic with your care team.
- (*Enactment Fidelity*) What, if anything, changed as a result of you working with the nurse? Your behavior (e.g. medication adherence, diet, or exercise), understanding of your health, your relationship with your other health care team members? Your ability to take care of your heart health? How?
- Compared to a year ago, how do you feel about managing your blood pressure or cholesterol and prevention of heart disease?

<u>Feasibility and Acceptability</u>

- How do you feel about completing this intervention? Tell me about any thoughts you might have about the potential for an intervention like this to become a part of regular care for addressing heart health in the HIV clinic.
- Was being in the intervention worth your time? Why? Would you recommend it to a friend or family member who also has HIV?
- How did it make you feel working with the nurse? How, if at all, did your relationship or trust with him/her evolve over the year?
- What did you like best about the intervention? What should we continue doing?
- What was hard about participating in the intervention with the nurse? What would you change about the intervention?

Secondary/Follow-up Questions

Follow-up "Probes" after significant statements are made:

[Earlier/A moment ago/when you first started speaking/when you were talking about x] you said [significant statement].

- Can you tell me more about that?
- Can you tell me more about how that affects [X]
- Can you clarify what you mean by [significant statement]
- Can you give me an example of a time when [significant statement] happened to you

Conclusion

What do you want me to know that I didn't ask you about? (YES return to interview; NO proceed)

We want to thank you so much for your participation and remind you that everything we have discussed will remain private. The audio file will be destroyed once this interview is transcribed, and the transcription will not contain your name or any identifying information.

Key Informant Interview Guide for Clinicians

Introduction

[Remind about Audio Recording and not to use real names]

Thank you for talking with us today. We are interested in learning more about your thoughts and beliefs about a recent intervention we tested in your HIV clinic. I am going to ask you some questions about these topics to hear about your experiences and perspectives. Please know that there is no right or wrong answer. You will notice that I won't give you a lot of feedback on your responses because I don't want to influence your answers. Finally, you are under no obligation to talk about anything that you are not comfortable discussing with me. Do you have any questions or concerns before we begin?

ADOPTION: Primary Questions for all Clinicians

- Can you tell me how you address cardiovascular risk in your patients living with HIV?
- Does your organization [name clinic] have training or tools on helping you manage cardiovascular disease risk in your patients living with HIV? Please tell me about those.
- Has the Nurse-led intervention changed how you talk with your patients about heart disease? If yes, tell me about that/those conversation(s)? Prompts: What prompted that discussion (e.g., did you bring it up) Did they ask you about their risk of developing heart disease? Did their primary care provider reach out to you? (Implementation)

ADOPTION: Intervention Dose and Fidelity

- Let's talk specifically about the intervention. As you may know, we had a nurse coordinator [name nurse at the site] for CVD management for HIV+ patients at high risk for CVD. Did you interact with this nurse? How often did you talk to this person? What sorts of conversations did you have? Was everything delivered by phone or did you come in in person? Which form of communication did you prefer?
- How did it make you feel working with this person? How did that evolve over the year?
- Did the nurse coordinator interact with your patient's primary care providers? How did that affect your workload?
- Was having the intervention in the clinic worthwhile? Why? Would you recommend it to a colleague at another clinic?
- (*Enactment Fidelity*) Did anything change as a result of you working with the nurse? Your behavior, understanding of your patients, your relationship with your other health care team members? How?
- What did you like best about the intervention? What should the clinic continue doing?
- What was hard about participating in the intervention with the nurse? What would you change about the intervention?

IMPLEMENTATION: Intervention Feasibly and Usability

Finally, we are interested in scaling up this intervention to help other patients reduce their risk of heart disease. However, we know in this and many clinics, there are cultural and systemic issues that can make a new clinic initiative more or less successful. We'd like to get your opinion on these issues and as a reminder, all information you provide will be confidential and aggregated across three clinics.

System

- Tell me about your clinic's culture. What aspects of it help you do your job well and what aspects can make it harder for you? What are the strengths of your clinic?
- What are the priorities of your clinic? Have they changed over time?
- Do you think your clinic values the prevention and treatment of cardiovascular disease in PLHIV?
- Does your clinic allocate any of its funding for the prevention and treatment of cardiovascular disease in PLHIV? If so, what proportion/amount would you estimate?
- Does your clinic track/monitor cardiovascular process metrics (e.g., number of CVD counselling sessions, lifestyle referrals, or BPs checked at each encounter)? Does it track/monitor cardiovascular outcome metrics (e.g., number of pts with controlled BP or lipids within normal limits)? If so, how is that information shared with you? How does that influence your own clinical practice?
- Is there anything that your HIV clinic currently does not have that would benefit your patients' cardiovascular health (e.g., funding, more staff, etc)
- Are there any cardiovascular health services that you currently do not offer that you would like to? (if yes, what is preventing you from providing these services)
- What areas of cardiovascular health do you think future research should focus on? (e.g. outcomes, cost benefit, mentor training, etc)

Secondary/Follow-up Questions

Follow-up "Probes" after significant statements are made:

[Earlier/A moment ago/when you first started speaking/when you were talking about x] you said [significant statement].

- Can you tell me more about that?
- Can you tell me more about how that affects [X]
- Can you clarify what you mean by [significant statement]

- Can you give me an example of a time when [significant statement] happened to you

Conclusion

Is there anything else you want us to know about [X]? (YES return to interview; NO proceed)

We want to thank you so much for your participation and remind you that everything we have discussed will remain private. The audio file will be destroyed once this interview is transcribed, and the transcription will not contain your name or any identifying information.

Key Informant Interview Guide for Prevention Nurses

Introduction

[Remind about Audio Recording and not to use real names]

Thank you for talking with us today. We are interested in learning more about your thoughts and beliefs about your participation in the EXTRA-CVD or AAIM-High clinical trials. I am going to ask you some questions to hear about your experiences and perspectives. Please know that there is no right or wrong answer. You will notice that I won't give you a lot of feedback on your responses because I don't want to influence your answers. Finally, you are under no obligation to talk about anything that you are not comfortable discussing with me. Do you have any questions or concerns before we begin?

ADOPTION: Intervention Dose and Fidelity

- Let's talk specifically about how the intervention was delivered at the [UH/Metro/Duke] HIV clinic. Tell me first about the range of your interactions with providers at [UH/Metro/Duke]. Were there some providers that embraced and engaged with the intervention? Were there some that were less enthusiastic or even antagonistic? What sorts of conversations did you have with providers? Did you interact by phone or did you come to clinic in person? Which form of communication did you prefer?
- How did your interactions evolve over the course of the 30 month trial period?
- Now tell me about your interactions with non-HIV providers. How often did you interact with primary care providers or non-HIV specialist providers outside of the HIV clinic? How were your interactions with these providers different than for the HIV clinic providers?
- (*Enactment Fidelity*) Do you believe that you were able to improve the cholesterol and blood pressure care of participants in the intervention? What aspects of the intervention were the most helpful in making these improvements? Are there any other outcomes you can think of that improved because of participation in the intervention (i.e., what other changes did you notice in participants?)
- What did you like best about the intervention? What should continue doing?
- What was hard about being the prevention nurse for this intervention? What would you change about the intervention?

MOTIVATIONAL INTERVIEWING

As part of the trial, you received specific training and ongoing coaching about motivational interviewing (MI) techniques to help the trial participants with behavior change. We are now going to ask you a few questions about your experience learning and using MI.

- We wonder if MI skills are important for the success of the intervention. What are your thoughts on this? Was the time and energy invested in training and coaching worth the results achieved?

- Are there any other outcomes (besides blood pressure and cholesterol changes) you can think of that improved because of participation in the intervention (i.e., what other changes did you notice in participants?)
- Describe the impact (if any) MI skills had on your relationship with participants (e.g., trust, engagement, empathy, etc.).
- How important do you think ongoing MI coaching and feedback would be for nurses working on similar interventions to improve their MI performance? What form should that coaching/feedback take? What would you keep or change about how the coaching was provided for this trial?
- What are the benefits to using MI which may not be easily captured in the main outcome measures of this trial (i.e. blood pressure and cholesterol change)?
- Reflecting on your use of MI with trial participants, do you think that there are trends in socioeconomic circumstances, demographics, behaviors, and/or health status that either help or deter the usefulness of MI to improve intervention outcomes? Similarly, are there underlying phenomena that make you feel more or less confident in successfully interacting with participants using the MI spirit or skills?
- Overall, how (if at all) have you incorporated MI into your interactions with participants? Probes: Did you find that you regularly embraced the spirit of MI and used MI skills, or was it something you felt you did only during process evaluation recordings?

IMPLEMENTATION: Intervention Feasibly and Usability

Finally, we are interested in scaling up this intervention to help other patients reduce their risk of heart disease. However, we know in this and many clinics, there are cultural and systemic issues that can make a new clinic initiative more or less successful. We'd like to get your opinion on these issues and as a reminder, all information you provide will be confidential and aggregated across three clinics.

System

- Tell me about the [UH/Metro/Duke] clinic's culture. What aspects of it helped you do your job well and what aspects made it harder for you? What are the strengths of your clinic?
- What are the priorities of your clinic? Have they changed over time?
- Do you think your clinic values the prevention and treatment of cardiovascular disease in PLHIV? How (if at all) have these values changed since the beginning of the trial?
- Does your clinic allocate any of its funding for the prevention and treatment of cardiovascular disease in PLHIV? If so, what proportion/amount would you estimate?
- Does your clinic track/monitor cardiovascular process metrics (e.g., number of CVD counselling sessions, lifestyle referrals, or BPs checked at each encounter)? Does it track/monitor cardiovascular outcome metrics (e.g., number of pts with controlled BP or lipids within normal limits)? If so, how is that information shared with you? How does that influence your own clinical practice?

- Is there anything that your clinic currently does not have that would benefit your patients' cardiovascular health (e.g., funding, more staff, etc)

- Are there any services that the clinic currently does not offer that would be valuable in addressing cardiovascular health? (if yes, what is preventing the clinic from providing these services)

- What areas of cardiovascular health do you think future research should focus on? (e.g. outcomes, cost benefit, mentor training, etc)

Secondary/Follow-up Questions

Follow-up "Probes" after significant statements are made:

[Earlier/A moment ago/when you first started speaking/when you were talking about x] you said [significant statement].

- Can you tell me more about that?
- Can you tell me more about how that affects [X]
- Can you clarify what you mean by [significant statement]
- Can you give me an example of a time when [significant statement] happened to you

Conclusion

Is there anything else you want us to know about [X]? (YES return to interview; NO proceed)

We want to thank you so much for your participation and remind you that everything we have discussed will remain private. The audio file will be destroyed once this interview is transcribed, and the transcription will not contain your name or any identifying information.

Appendix T: Examining the role of context on the adaptability of the EXTRA-CVD intervention through transportability analysis, stakeholder engagement, and the construction of a dose composite measure

Study Protocol and Manual of Procedures

Lead investigator: Angela Aifah, PhD, AM; NYU Grossman School of Medicine
Funded by: NHLBI (U01HL142099-S1; Diversity Supplement)
Date & Version no.: 18th July 2022, version 1

1. PROTOCOL SUMMARY

Study Description: This study uses mixed-methods approaches and stakeholder feedback to develop fa composite measure of dose (i.e. the dose delivered and dose received) to explain the effectiveness of the EXTRA-CVD intervention of the parent study [*A nurse-led intervention to **EX**tend the HIV **TR**eatment c**A**scade for **C**ardio**V**ascular **D**isease prevention (EXTRA-CVD);* (U01HL142099)].

Aim: To develop a pragmatic dose composite measure (i.e., the frequency and intensity) for the EXTRA-CVD intervention.

Objectives: Use a group concept mapping approach of stakeholder engagement and the Delphi method to formulate the dose composite measure.

Endpoints: Dose composite measure of the parent study, EXTRA-CVD.

Study population: Stakeholders, including those who have participated in previous stakeholder activities for the parent study (design team). These individuals include the EXTRA-CVD nurses, physicians, social workers, dieticians etc.

Study duration: 6 months

2. SCHEDULE OF ACTIVITIES (in months)

Activity	M-1	M-2	M-3	M-4	M-5	M-6
Finalize protocol & obtain IRB approval	X					
Send invitations to participants	X					
GCM steps 1 to 4		X	X			
GCM steps 5 & 6				X		
Delphi rounds 1 to 3					X	

**Share findings with stakeholders & research team**						**X**

3. BACKGROUND

Within United States HIV clinics, there is limited evidence examining the effectiveness of implementation strategies to address the burden of comorbid HIV and atherosclerotic cardiovascular disease (ASCVD). Equally important to implementation effectiveness is the dose of an intervention – i.e. the frequency or duration of exposure to the intervention. Research from evidence based interventions (EBIs) underscore the importance of dose on the adoption and sustainability of interventions within routine practice care settings. While agreement on the link between dose and intervention outcomes is clear, there is less evidence in support of the development of optimal dose composite measures for EBIs, particularly pragmatic measures which include stakeholder feedback.

Stakeholder-engaged research is an important driving force for the implementation of science-led behavioral interventions, particularly as it pertains to engaging stakeholders early and frequently throughout the implementation period. More importantly, while most approaches for stakeholder-engaged research typically incorporate qualitative methods, robust mixed-method approaches such as group concept mapping (GCM) are increasingly being applied to implementation research for a number of reasons including developing criteria for pragmatic measures. GCM is a novel, participatory approach to stakeholder engagement and topic development, whereby the perceptions of participants on a specific subject are elicited and then used to generate illustrative conceptual frameworks of the target group or end-users views.

A recent study applied a two-step approach for engaging stakeholders by using GCM and the Delphi method to operationalize pragmatic measures for implementation science outcomes (i.e. acceptability, appropriateness, and feasibility) and establish rating criteria for assessing the construct. In particular, Powell et al. apply the Delphi technique as a follow-up step to better refine and consolidate the list of criteria generated from the GCM step. The consensus-building approach of Delphi uses an iterative process to collect data from key or expert stakeholders in a pre-specified sequence. The use of mixed-methods in stakeholder analysis such as group concept mapping and the Delphi technique offers unique opportunities to enhance the adoption of EBIs through the development of a pragmatic dose composite measure. The current study similarly incorporates the two-step process of GCM and Delphi to engage key stakeholders in developing a dose composite measure for the parent study.

Total dose of complex multi-component interventions is often difficult to conceptualize. For example, the EXTRA-CVD intervention consists of 4 primary components (nurse-led care coordination, home blood pressure (BP) monitoring, algorithms for evidence-based prescribing of BP meds, and electronic health records tools). Each of these 4 components is further composed of multiple activities including telephone calls with patients, telephone calls with providers, prescription of new medications or uptitration of medication, home blood pressure monitoring, etc…) Some of these may be more or less important in the eyes of key stakeholders who include patients, doctors, clinic nurses, and the EXTRA-CVD interventionist nurses. For example, how does one weight the value of a 15 minute phone conversation with the patient vs. a 15 minute phone conversation with a prescribing doctor? The aim of this study is to engage these stakeholders in the construction of an a priori composite measure of dose that is based on personal and clinical experience. This measure of dose will then be used as a potential mediator of EXTRA-CVD outcomes, including both primary and secondary clinical outcomes (BP & cholesterol) but also implementation outcomes and other outcomes of interest (i.e. time to disengagement for those who are lost to follow-up).

4. STUDY AIM & OBJECTIVE

Aim	Objective
To develop a composite (or hybrid) dose measure of the EXTRA-CVD intervention.	Conduct stakeholder engaged activities through group concept mapping and the Delphi method.

5. STUDY DESIGN

The objective of this study will be to use a GCM approach along with the Delphi method to engage roughly twenty EXTRA-CVD stakeholders on constructing a pragmatic dose composite measure for the parent study intervention.

Two-part approach: **first**, use group concept mapping to identify factors important for dose composite measure and **second,** use the Delphi process to develop a final dose compose measure.

All activities for the GCM phase will be conducted online using the Concept Systems software and will include one full group meeting for step 5, which will be done via Zoom or WebEx. The Delphi process activities, i.e. questionnaires, will be conducted online.

5.1 Part A: Group Concept Mapping

Group Concept Mapping (GCM) is a six-step process: 1) preparation, 2) generation, 3) structuring, 4) representation, 5) interpretation, and 6) utilization. See Figure 1 for an overview of the GCM steps. Below are the definitions for the six steps along with the activities that will take place at each step for this study. The descriptions of the steps are based on the work and explanations provided by Burke et al. (2005).

- Step 1, Preparation: Identify key focal topics (e.g., developing the prompt for participants to respond to or address) and determine participant selection criteria. For this study, the prompt will be *"Based on your review of the data for the EXTRA-CVD dose received and delivered what are some factors that you think are important for putting together a dose measure (i.e., considering both dose received and delivered) for the intervention?"* This prompt will be shared with participants via a link sent from the Concept Systems program. Participants will be asked to create an account from which they can easily complete all of the activities online or on the web platform.
- Step 2, Generation: Participants respond to the prompt and provide a list ofresponses that will be used data collection and analysis. In this study, participants will be asked to provide up to 8 responses to the prompt question.
- Step 3, Structuring: Participants independently sort and rate the complete list of responses or items. Participants will sort the responses or items into piles of statements

based on their perceived similarity. Following the sorting of the responses, participants then rate the responses according to its importance or usefulness to the focal question.

- Step 4, Representation: Once all of the sorting-and-rating data is collected, the Concept Systems software will then analyze the data to provide quantitative summaries and visual representations of the relationship between and importance of the responses. As noted by Burke et al. (2005), the visual representations or concept maps are "based on a sequence of analyses that includes most prominently multidimensional scaling and hierarchical cluster analysis. All analysis will be conducted by the study investigator and completed using the Concept Systems software.
- Step 5, Interpretation: In a group setting, participants will be asked to provide feedback on the concept maps – particularly to discuss the cluster domains (and individual responses represented in the clusters) produced from the sorting and rating activities.
- Step 6, Utilization: For this final step, the findings will be discussed among the investigative team to determine how they best inform the original prompt.

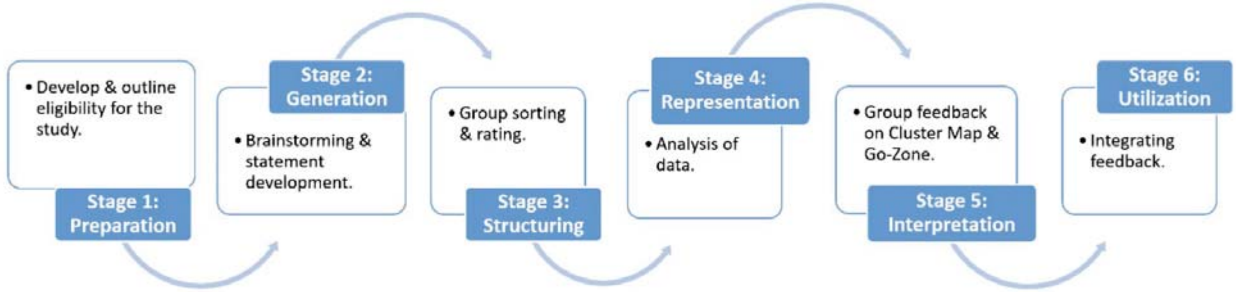

FIGURE 1: OVERVIEW OF GCM STEPS

Expected outcome of the GCM phase: Shortlist of factors that participants believe best represent an optimal dose measure for the EXTRA-CVD intervention. The data source for the GCM component will be based on the process evaluation measures on the dose received and delivered from the parent study. Following GCM, the Delphi process will incorporate the GCM-generated list of factors to establish a consensus on the criteria for potential dosage measures for the intervention. The procedure for the Delphi Process is noted below.

5.2 Part B: Delphi Process

The Delphi process will include 3 rounds of questionnaires to reach a general consensus on an appropriate and acceptable dose measure for the parent study. Areas of consideration for the Delphi participants will be selecting key components for the dose composite measure as well as deciding how to effectively "weight" the value of the components for the measure. Throughout the process, participants will be asked to rate or rank-order items which they believe are most applicable until a consensus is reached during the final round on the key factors for a dose composite measure. The questionnaires will be accessible via a link that will be emailed to participants. Using an online link will allow for subject anonymity – an advantage of the Delphi technique.

6. STUDY POPULATION

Individuals who meet the following study eligibility will be included in the stakeholder activities.

Inclusion criteria

- Participant in previous EXTRA-CVD or AAIM-HIGH Design Team sessions.
- EXTRA-CVD or AAIM-High study personnel
- Able to provide consent.

Exclusion criteria

- There are no exclusion criteria

7. STUDY ACTIVITIES

7.1 Recruitment of participants

Individuals from the two geographic sites (i.e. Cleveland, Ohio and Durham, North Carolina) who participated in the human-centered design process of the parent study who indicated on their previous consent form that they would be willing to participate in future studies will be invited to participate in this study. Our goal sample size is 20 total participants, with a minimum of 20 total participants. If we do not reach our goal sample through invitations to design team members, then we will invite EXTRA-CVD or AAIM-High study personnel to participate. The design teams were well-balanced in terms of representation from patients, clinic staff, clinic nurses, HIV specialists, non-HIV specialists (e.g. cardiologists), and primary care doctors. We will seek to maintain this balance of representation for the current study.

7.2 Informed consent

To invite design team members to participate in this study, the study team coordinators at each site will send an e-mail to members who have previously consented to be contacted for future research. The email will explain the purpose and procedures of the new activities and will include a REDCap survey link to a form that will capture the participant's interest in participating in or declining the invitation to participate. For those that decline, the REDCap survey will end, thanking the member for their time. For those that are interested in participating, the REDCap survey will inform the design team member that a study team member will need to contact them to complete the informed consent process, and the survey will capture the preferred communication method to schedule that virtual visit (e-mail or phone contact information). A study team member will then contact the design team member through phone or e-mail to schedule the virtual informed consent visit and explain the process. A study team member will email a REDCap survey link to the full version of the consent document to the participant on the scheduled date. This virtual consent will capture an electronic signature and follow most of the guidelines laid out by the UH IRB in their

document titled Guidelines for Remote Electronic Consent. An exception will be the audiovisual visit to confirm identity, as the study team has determined that this will place unnecessary burden on the design team members. All design team members have worked closely with the study team in the past and their identity is well-known to the study team. Aligning with data previously captured from participants, the study team member will ask the participant for their full name, professional title at their respective site, and their e-mail address to confirm their identity over the phone, and will then perform verbal informed consent while the participant reads along through the REDCap survey link. The participant will then provide their electronic signature in RedCap confirming their consent to participate.

7.3 Discontinuation/ Withdrawal of Participants from Study

Each participant has the right to withdraw from the study at any time. In addition, the Investigator may discontinue a participant from the study at any time if the Investigator considers it necessary for any reason including:

- Ineligibility either arising during the study period or retrospectively having been overlooked during recruitment or consent.
- Significant protocol deviation.
- Significant non-compliance with study requirements.
- Withdrawal of consent.

8. DATA ANALYSIS

The GCM will be administered via an online link such that participants will be able to respond to a focus prompt, e.g. “Based on your review of the data for the EXTRA-CVD dose received and delivered what are some factors that you think are important for putting together a dose measure (i.e. considering both dose received and delivered) for the intervention?”. Once participants respond to the focus prompt and the collected data is organized to remove redundant or similar statements, they will be given the opportunity to rate and sort the statements based on pre-established criteria. Following the rating and sorting activity, the collected data will then be analyzed using the online Concept Systems Global MAX software (The Concept System Global MAX, 2019) and based on multidimensional scaling (MDS) with a dimensional solution to arrange and produce cluster maps which show the thematic associations of the statements. Participants will then have a focus group meeting to provide feedback on the cluster maps and choose the cluster map of statements which they believe will best represent the key constructs or variables for developing a pragmatic dose composite measure for the intervention.

Based on the combined results from the GCM focus groups from both sites, the Delphi technique will then be used to develop a final composite measure based on the questionnaire responses of stakeholders from 3 iterations/ rounds. During each round, the data collected from the previous round is consolidated and assessed by the researcher in order to develop a questionnaire that includes the variables or items rated highly by the participants. The outcome of this final step will be a composite dose measure for the parent intervention.

8.1 Data Management

All data from the GCM activities (including the collection of basic participant demographics) will be identifiable only by a participant ID number and will not contain PHI. It will be securely housed on the Concept Systems Global Max software and will be password protected. The Lead Investigator and research assistant will be the only individuals that will have access to the GCM data.

The Delphi activities' data (i.e. outcomes of the 3 questionnaire or survey rounds) will be kept on a secure network using UH REDCap and again only linked to participant ID number. The Lead Investigator and research assistant will be only individuals that will have access to the Delphi data.

9. PARTICIPANT CONFIDENTIALITY

The study staff will ensure participants' anonymity is maintained. Participants will be identified only by a participant ID number on all study related documents and electronic databases, with the exception of the in-person GCM activities (i.e. step 5), where participants can share their perspectives and will be addressed by their preferred name. All documents will be stored securely and only accessible by study staff and authorized personnel.

10. EXPENSES AND BENEFITS

Participants will be compensated for their time and effort on this study through $100 payments distributed by each site in accordance with their local policies. This payment will be provided at the end of all stakeholder engagement activities.

References:

1. Weiser J, Beer L, West BT, Duke CC, Gremel GW, Skarbinski J. Qualifications, Demographics, Satisfaction, and Future Capacity of the HIV Care Provider Workforce in the United States, 2013-2014. Clin Infect Dis 2016;63:966-75.
2. Freiberg MS, Chang CC, Kuller LH, et al. HIV infection and the risk of acute myocardial infarction. JAMA Intern Med 2013;173:614-22.
3. Longenecker CT, Triant VA. Initiation of antiretroviral therapy at high CD4 cell counts: does it reduce the risk of cardiovascular disease? Curr Opin HIV AIDS 2014;9:54-62.
4. Althoff K, Palella F, Gebo K, et al. Impact of Smoking, Hypertension & Cholesterol on Myocardial Infarction in HIV+ Adults. Conference on Retroviruses and Opportunistic Infections. Seattle, WA, USA2017.
5. Clement ME, Park LP, Navar AM, et al. Statin Utilization and Recommendations Among HIV- and HCV-infected Veterans: A Cohort Study. Clin Infect Dis 2016;63:407-13.
6. Al-Kindi SG, Zidar DA, McComsey GA, Longenecker CT. Gender Differences in Statin Prescription Rate Among Patients Living With HIV and Hepatitis C Virus. Clin Infect Dis 2016;63:993-4.
7. Myerson M, Poltavskiy E, Armstrong EJ, Kim S, Sharp V, Bang H. Prevalence, treatment, and control of dyslipidemia and hypertension in 4278 HIV outpatients. J Acquir Immune Defic Syndr 2014;66:370-7.
8. Cioe PA, Crawford SL, Stein MD. Cardiovascular risk-factor knowledge and risk perception among HIV-infected adults. J Assoc Nurses AIDS Care 2014;25:60-9.
9. Cheng QJ, Engelage EM, Grogan TR, Currier JS, Hoffman RM. Who Provides Primary Care? An Assessment of HIV Patient and Provider Practices and Preferences. J AIDS Clin Res 2014;5.
10. Ho PM, Lambert-Kerzner A, Carey EP, et al. Multifaceted intervention to improve medication adherence and secondary prevention measures after acute coronary syndrome hospital discharge: a randomized clinical trial. JAMA Intern Med 2014;174:186-93.
11. Bosworth HB, Powers BJ, Olsen MK, et al. Home blood pressure management and improved blood pressure control: results from a randomized controlled trial. Arch Intern Med 2011;171:1173-80.
12. Bosworth HB, Olsen MK, Grubber JM, et al. Two self-management interventions to improve hypertension control: a randomized trial. Ann Intern Med 2009;151:687-95.
13. Pastakia SD, Manyara SM, Vedanthan R, et al. Impact of Bridging Income Generation with Group Integrated Care (BIGPIC) on Hypertension and Diabetes in Rural Western Kenya. J Gen Intern Med 2017;32:540-8.
14. Bosworth HB, Olsen MK, McCant F, et al. Hypertension Intervention Nurse Telemedicine Study (HINTS): testing a multifactorial tailored behavioral/educational and a medication management intervention for blood pressure control. Am Heart J 2007;153:918-24.
15. Kumar A, Kamano JH, Too K, et al. Effect of Nurse-Based Management of Hypertension in Rural Western Kenya. American College of Cardiology Scientific Sessions. Chicago, IL2016.
16. 90-90-90: an ambitious treatment target to help end the aids epidemic. Geneva, Switzerland: UNAIDS Joint United Nations Programme on HIV/AIDS; 2014.
17. HIV/AIDS Bureau Performance Measures. US Department of Health and Human Services, 2015. (Accessed July 14, 2017, at https://hab.hrsa.gov/deliverhivaidscare/coremeasures.pdf.)
18. National Institutes of Health. PA-17-194 Targeted Implementation Science to Achieve 90/90/90 Goals for HIV/AIDS Prevention and Treatment (R01) (Accessed July 14, 2017, at https://grants.nih.gov/grants/guide/pa-files/PA-17-194.html.)

19. Linkages across the Continuum of HIV Services for Key Populations Affected by HIV (LINKAGES). (Accessed December 26, 2018, at https://www.fhi360.org/projects/linkages-across-continuum-hiv-services-key-populations-affected-hiv-linkages.)
20. Chobanian AV, Bakris GL, Black HR, et al. The Seventh Report of the Joint National Committee on Prevention, Detection, Evaluation, and Treatment of High Blood Pressure: the JNC 7 report. JAMA 2003;289:2560-72.
21. Mancia G, Fagard R, Narkiewicz K, et al. 2013 ESH/ESC guidelines for the management of arterial hypertension: the Task Force for the Management of Arterial Hypertension of the European Society of Hypertension (ESH) and of the European Society of Cardiology (ESC). Eur Heart J 2013;34:2159-219.
22. Whelton PK, Carey RM, Aronow WS, et al. 2017 ACC/AHA/AAPA/ABC/ACPM/AGS/APhA/ASH/ASPC/NMA/PCNA Guideline for the Prevention, Detection, Evaluation, and Management of High Blood Pressure in Adults: A Report of the American College of Cardiology/American Heart Association Task Force on Clinical Practice Guidelines. Hypertension 2017.
23. Bosworth HB, Powers BJ, Oddone EZ. Patient self-management support: novel strategies in hypertension and heart disease. Cardiol Clin 2010;28:655-63.
24. Dube MP, Stein JH, Aberg JA, et al. Guidelines for the evaluation and management of dyslipidemia in human immunodeficiency virus (HIV)-infected adults receiving antiretroviral therapy: recommendations of the HIV Medical Association of the Infectious Disease Society of America and the Adult AIDS Clinical Trials Group. Clin Infect Dis 2003;37:613-27.
25. Jacobson TA, Maki KC, Orringer CE, et al. National Lipid Association Recommendations for Patient-Centered Management of Dyslipidemia: Part 2. J Clin Lipidol 2015;9:S1-S122 e1.
26. Jacobson TA, Ito MK, Maki KC, et al. National Lipid Association recommendations for patient-centered management of dyslipidemia: part 1 - executive summary. J Clin Lipidol 2014;8:473-88.
27. Stone NJ, Robinson JG, Lichtenstein AH, et al. 2013 ACC/AHA guideline on the treatment of blood cholesterol to reduce atherosclerotic cardiovascular risk in adults: a report of the American College of Cardiology/American Heart Association Task Force on Practice Guidelines. Circulation 2014;129:S1-45.
28. Cannon CP, Blazing MA, Giugliano RP, et al. Ezetimibe Added to Statin Therapy after Acute Coronary Syndromes. N Engl J Med 2015;372:2387-97.
29. Sabatine MS, Giugliano RP, Keech AC, et al. Evolocumab and Clinical Outcomes in Patients with Cardiovascular Disease. N Engl J Med 2017;376:1713-22.
30. Jarcho JA, Keaney JF, Jr. Proof That Lower Is Better--LDL Cholesterol and IMPROVE-IT. N Engl J Med 2015;372:2448-50.
31. Silverman MG, Ference BA, Im K, et al. Association Between Lowering LDL-C and Cardiovascular Risk Reduction Among Different Therapeutic Interventions: A Systematic Review and Meta-analysis. JAMA 2016;316:1289-97.
32. Averting a Crisis in HIV Care: A Joint Statement of the American Academy of HIV Medicine (AAHIVM) and the HIV Medicine Association (HIVMA) On the HIV Medical Workforce. 2009. (Accessed July 14, 2017, at http://www.idsociety.org/uploadedFiles/IDSA/Policy_and_Advocacy/Current_Topics_and_Issues/Workforce_and_Training/Statements/AAHIVM%20HIVMA%20Workforce%20Statement%20062509.pdf.)
33. Institute of Medicine, Committe on HIV Screening and Access to Care. HIV screening and access to care: health care system capacity for increased HIV testing and provision of care. 2011. (Accessed July 14, 2017, at http://www.nap.edu/catalog/13074.html.)
34. Barnes R, Koester KA, Waldura JF. Attitudes about providing HIV care: voices from publicly funded clinics in California. Fam Pract 2014;31:714-22.
35. O'Neill M, Karelas GD, Feller DJ, et al. The HIV Workforce in New York State: Does Patient Volume Correlate with Quality? Clin Infect Dis 2015;61:1871-7.

36. Landovitz RJ, Desmond KA, Gildner JL, Leibowitz AA. Quality of Care for HIV/AIDS and for Primary Prevention by HIV Specialists and Nonspecialists. AIDS Patient Care STDS 2016;30:395-408.
37. Kimmel AD, Martin EG, Galadima H, et al. Clinical outcomes of HIV care delivery models in the US: a systematic review. AIDS care 2016;28:1215-22.
38. Dawson-Rose C, Cuca YP, Webel AR, et al. Building Trust and Relationships Between Patients and Providers: An Essential Complement to Health Literacy in HIV Care. The Journal of the Association of Nurses in AIDS Care : JANAC 2016;27:574-84.
39. The American Academy of HIV Medicine. HIV Specialist in Crisis: The HIV Workforce. . 2016. (Accessed July 14, 2017, at https://aahivm.org/wp-content/uploads/2017/03/FINAL-MARCH-2016.pdf.)
40. Wilson IB, Landon BE, Hirschhorn LR, et al. Quality of HIV care provided by nurse practitioners, physician assistants, and physicians. Ann Intern Med 2005;143:729-36.
41. Shaw RJ, McDuffie JR, Hendrix CC, et al. Effects of nurse-managed protocols in the outpatient management of adults with chronic conditions: a systematic review and meta-analysis. Ann Intern Med 2014;161:113-21.
42. van Driel ML, Morledge MD, Ulep R, Shaffer JP, Davies P, Deichmann R. Interventions to improve adherence to lipid-lowering medication. Cochrane Database Syst Rev 2016;12:CD004371.
43. Feinstein MJ, Bogorodskaya M, Bloomfield GS, et al. Cardiovascular Complications of HIV in Endemic Countries. Curr Cardiol Rep 2016;18:113.
44. Vedanthan R, Kamano JH, Lee H, et al. Bridging Income Generation with Group Integrated Care for cardiovascular risk reduction: Rationale and design of the BIGPIC study. Am Heart J 2017;188:175-85.
45. Barasa FA, Vedanthan R, Pastakia SD, et al. Approaches to Sustainable Capacity Building for Cardiovascular Disease Care in Kenya. Cardiol Clin 2017;35:145-52.
46. Naanyu V, Vedanthan R, Kamano JH, et al. Barriers Influencing Linkage to Hypertension Care in Kenya: Qualitative Analysis from the LARK Hypertension Study. J Gen Intern Med 2016;31:304-14.
47. Vedanthan R, Kamano JH, Bloomfield GS, Manji I, Pastakia S, Kimaiyo SN. Engaging the Entire Care Cascade in Western Kenya: A Model to Achieve the Cardiovascular Disease Secondary Prevention Roadmap Goals. Glob Heart 2015;10:313-7.
48. Vedanthan R, Kamano JH, Naanyu V, et al. Optimizing linkage and retention to hypertension care in rural Kenya (LARK hypertension study): study protocol for a randomized controlled trial. Trials 2014;15:143.
49. Huck DM, Nalubwama H, Longenecker CT, Frank SH, Okello E, Webel AR. A qualitative examination of secondary prophylaxis in rheumatic heart disease: factors influencing adherence to secondary prophylaxis in Uganda. Glob Heart 2015;10:63-9 e1.
50. Webel AR, Longenecker CT, Gripshover B, Hanson JE, Schmotzer BJ, Salata RA. Age, stress, and isolation in older adults living with HIV. AIDS Care 2014;26:523-31.
51. Dirajlal-Fargo S, Webel AR, Longenecker CT, et al. The effect of physical activity on cardiometabolic health and inflammation in treated HIV infection. Antivir Ther 2016;21:237-45.
52. Longenecker CT, Hileman CO, Funderburg NT, McComsey GA. Rosuvastatin preserves renal function and lowers cystatin C in HIV-infected subjects on antiretroviral therapy: the SATURN-HIV trial. Clin Infect Dis 2014;59:1148-56.
53. Hileman CO, Longenecker CT, Carman TL, McComsey GA. C-reactive protein predicts 96-week carotid intima media thickness progression in HIV-infected adults naive to antiretroviral therapy. J Acquir Immune Defic Syndr 2014;65:340-4.
54. Longenecker CT, Hileman CO, Carman TL, et al. Vitamin D supplementation and endothelial function in vitamin D deficient HIV-infected patients: a randomized placebo-controlled trial. Antivir Ther 2012;17:613-21.
55. Glasgow RE, Vogt TM, Boles SM. Evaluating the public health impact of health promotion interventions: the RE-AIM framework. Am J Public Health 1999;89:1322-7.

56. Abrams DB, Orleans CT, Niaura RS, Goldstein MG, Prochaska JO, Velicer W. Integrating individual and public health perspectives for treatment of tobacco dependence under managed health care: a combined stepped-care and matching model. Ann Behav Med 1996;18:290-304.
57. Amico KR, Barta W, Konkle-Parker DJ, et al. The information-motivation-behavioral skills model of ART adherence in a Deep South HIV+ clinic sample. AIDS Behav 2009;13:66-75.
58. Fisher JD, Amico KR, Fisher WA, Harman JJ. The information-motivation-behavioral skills model of antiretroviral adherence and its applications. Curr HIV/AIDS Rep 2008;5:193-203.
59. Fisher JD, Fisher WA. Changing AIDS-risk behavior. Psychol Bull 1992;111:455-74.
60. Steinhardt MA, Dishman RK. Reliability and validity of expected outcomes and barriers for habitual physical activity. J Occup Med 1989;31:536-46.
61. Spirig R, Moody K, Battegay M, De Geest S. Symptom management in HIV/AIDS: advancing the conceptualization. ANS Adv Nurs Sci 2005;28:333-44.
62. Ashford S, Edmunds J, French DP. What is the best way to change self-efficacy to promote lifestyle and recreational physical activity? A systematic review with meta-analysis. Br J Health Psychol 2010;15:265-88.
63. Kelly S, Melnyk BM, Belyea M. Predicting physical activity and fruit and vegetable intake in adolescents: a test of the information, motivation, behavioral skills model. Res Nurs Health 2012;35:146-63.
64. Williams SL, French DP. What are the most effective intervention techniques for changing physical activity self-efficacy and physical activity behaviour--and are they the same? Health Educ Res 2011;26:308-22.
65. Webel AR, Perazzo J, Dawson-Rose C, et al. A qualitative investigation of the perspectives and drivers of exercise and dietary behaviors in PLHIV around the globe. . Applied Nursing Research 2017;In Press.
66. Webel AR, Cuca Y, Okonsky JG, Asher AK, Kaihura A, Salata RA. The impact of social context on self-management in women living with HIV. Soc Sci Med 2013;87:147-54.
67. Chang AY, Nabaale J, Nalubwama H, et al. Characteristics and Motivations of Women of Reproductive Age in Uganda with Rheumatic Heart Disease: A Mixed Methods Study. . Annals of Global Health 2017;83:171-2.
68. Slomka J, Prince-Paul M, Webel A, Daly BJ. Multimorbidity With HIV: Views of Community-Based People Living With HIV and Other Chronic Conditions. J Assoc Nurses AIDS Care 2017;28:603-11.
69. Brown T, Katz B. Change by Design. Journal of Product Innovation Management 2011;28:381-3.
70. Leavy B. Design thinking- a new mental model of value innovation. Strategy & Leadership 2010;38:5-14.
71. Brown T, Wyatt J. Design Thinking for Social Innovation. Stanford Social Innovation Review 2010;8:30-5.
72. Saunders RP, Evans MH, Joshi P. Developing a process-evaluation plan for assessing health promotion program implementation: a how-to guide. Health Promot Pract 2005;6:134-47.
73. Devaney B, Rossi P. Thinking through evaluation design options. Children and Youth Services Review 1997;19:587-606.
74. Helitzer D, Yoon SJ, Wallerstein N, Dow y Garcia-Velarde L. The role of process evaluation in the training of facilitators for an adolescent health education program. J Sch Health 2000;70:141-7.
75. Tovar EG, Rayens MK, Clark M, Nguyen H. Development and psychometric testing of the Health Beliefs Related to Cardiovascular Disease Scale: preliminary findings. J Adv Nurs 2010;66:2772-84.
76. Dedoose Version 7.0.23, web application for managing, analyzing, and presenting qualitative and mixed method research data. Los Angeles, CA: SocioCultural Research Consultants, LLC; 2016.
77. De Vries H, Elliott MN, Kanouse DE, Teleki SS. Using pooled kappa to summarize interrater agreement across many items. Field Methods 2008;20:272-82.

78. Bernard H. Research methods in anthropology: Qualitative and quantitative approaches. Walnut Creek, CA: AltaMira Press; 2002.
79. Mogobe KD, Shaibu S, Matshediso E, et al. Language and Culture in Health Literacy for People Living with HIV: Perspectives of Health Care Providers and Professional Care Team Members. AIDS Res Treat 2016;2016:5015707.
80. Nastasi BK, Varjas K, Schensul SL, Tudor Silva K, Schensul JJ, Ratnayake P. The Participatory Intervention Model: A Framework for Conceptualizing and Promoting Intervention Acceptability. School Psychology Quarterly 2000;15:207-32.
81. Grundy SM, Stone NJ, Bailey AL, et al. 2018 AHA/ACC/AACVPR/AAPA/ABC/ACPM/ADA/AGS/APhA/ASPC/NLA/PCNA Guideline on the Management of Blood Cholesterol: A Report of the American College of Cardiology/American Heart Association Task Force on Clinical Practice Guidelines. J Am Coll Cardiol 2018.
82. Guyton JR, Bays HE, Grundy SM, Jacobson TA, The National Lipid Association Statin Intolerance P. An assessment by the Statin Intolerance Panel: 2014 update. J Clin Lipidol 2014;8:S72-81.
83. Rosenson RS, Baker SK, Jacobson TA, Kopecky SL, Parker BA, The National Lipid Association's Muscle Safety Expert P. An assessment by the Statin Muscle Safety Task Force: 2014 update. J Clin Lipidol 2014;8:S58-71.
84. Yarows SA, Amerena JV. Determination of accuracies of 10 models of home blood pressure monitors using an oscillometric simulator. Blood Press Monit 1999;4:45-52.
85. Moller DS, Dideriksen A, Sorensen S, Madsen LD, Pedersen EB. Tele-monitoring of home blood pressure in treated hypertensive patients. Blood Press 2003;12:56-62.
86. Tsuji I, Imai Y, Nagai K, et al. Proposal of reference values for home blood pressure measurement: prognostic criteria based on a prospective observation of the general population in Ohasama, Japan. Am J Hypertens 1997;10:409-18.
87. Cheung A, Weir M, Mayhew A, Kozloff N, Brown K, Grimshaw J. Overview of systematic reviews of the effectiveness of reminders in improving healthcare professional behavior. Syst Rev 2012;1:36.
88. Black AD, Car J, Pagliari C, et al. The impact of eHealth on the quality and safety of health care: a systematic overview. PLoS Med 2011;8:e1000387.
89. Verbeke G, Molenberghs G. Linear mixed models in practice: a SAS oriented approach. New York: Springer-Verlag; 1997.
90. Longenecker CT, Sattar A, Gilkeson R, McComsey GA. Rosuvastatin slows progression of subclinical atherosclerosis in patients with treated HIV infection. AIDS 2016;30:2195-203.
91. Blood Pressure Lowering Treatment Trialists C, Ninomiya T, Perkovic V, et al. Blood pressure lowering and major cardiovascular events in people with and without chronic kidney disease: meta-analysis of randomised controlled trials. Bmj 2013;347:f5680.
92. Goff DC, Jr., Lloyd-Jones DM, Bennett G, et al. 2013 ACC/AHA guideline on the assessment of cardiovascular risk: a report of the American College of Cardiology/American Heart Association Task Force on Practice Guidelines. Circulation 2014;129:S49-73.

EXTRA-CVD: A nurse-led intervention to Extend the HIV Treatment Cascade for Cardiovascular Disease Prevention

Statistical Analysis Plan: Aim 2

Principal Investigators: Christopher Longenecker, MD; Allison Webel, RN, PhD; Hayden Bosworth, PhD

Background

The HIV/AIDS treatment cascade model was developed to assess how people living with HIV infection (PLHIV) access care and treatment. The model includes sequential steps in care including—(1) diagnosis, (2) prescription of appropriate antiretroviral therapy (ART), and (3) suppression of detectable HIV virus in the blood. These metrics are familiar to HIV-providers and integral to continuous quality improvement initiatives at HIV specialty clinics across the United States, where most PLHIV receive care.

PLHIV are known to have a 1.5-2x higher risk of atherosclerotic cardiovascular disease (ASCVD) compared to uninfected individuals, a risk that persists despite viral suppression on ART. Thus, once PLHIV achieve the final step of the HIV treatment cascade, providers have an important opportunity to focus on preventing ASCVD and other non-AIDS comorbidities. We envision extending the treatment cascade for high blood pressure (BP) and high cholesterol, which account for much of the population-level ASCVD risk in PLHIV4, as follows: Step 1, appropriate screening and diagnosis; Step 2, appropriate treatment; and Step 3, achievement of guideline-based treatment targets. Currently, PLHIV are sub-optimally treated for high BP and cholesterol, possibly due to low perceived risk for ASCVD or challenges in primary care coordination between HIV specialists and non-HIV providers. Non-physician led approaches may address these barriers.

Our overarching goal is to improve the BP and cholesterol treatment cascade for PLHIV on suppressive ART to reduce ASCVD risk. Guided by a RE-AIM framework (Reach x Efficacy—Adoption, Implementation, Maintenance), and using a mixed-methods clinical effectiveness trial design, our experienced multi-disciplinary team will test a contextually adapted ASCVD prevention nurse-led intervention (EXTRA-CVD) to reach guideline-based BP and cholesterol targets. The study will be conducted in three racially and ethnically diverse clinic contexts that are broadly representative of HIV specialty care in the US.

Aims

Aim 1: Conduct a baseline assessment of ASCVD preventive care and perceptions of ASCVD risk in the HIV specialty clinic environment.

Sub-Aim 1.1: Adapt the EXTRA-CVD intervention components to the HIV specialty clinic context with key stakeholder input and data from the baseline assessments

Aim 2: **Evaluate the 12-month efficacy of the EXTRA-CVD intervention to improve BP and cholesterol control in PLHIV (Aim 2)**

Aim 3: Conduct a process evaluation of the EXTRA-CVD intervention (Aim 3)

Hypothesis

We hypothesize that our prevention nurse-led intervention will lead to a clinically significant 6mmHg reduction in systolic BP and 15mg/dL reduction in non-HDL cholesterol over 12 months compared to those receiving general prevention education only.

Study Design

Two-arm parallel randomized controlled clinical trial

Data Source

Primary data collection with trial participants.

- Vitals:
    - Blood pressure: measured by RA, twice in each arm 1 minute apart. The mean of the two right arm BP measurements will be used in analysis (systolic BP, primary outcome). (0, 4, 8, 12 months)
    - Lipid profile: non-HDL cholesterol (secondary outcome), total cholesterol, triglycerides, HDL, LDL (exploratory outcomes); measured by lab personnel blinded to treatment arm (0, 4, 8, 12 months)
- Self-report measures
    - Sociodemographic characteristics
    - Medical history
    - Behavioral factors
- ASCVD risk estimator (0, 12 months)
    - ASCVD lifetime risk
    - 10-year risk
        - categorized as <20% 10-year risk vs. ≥20% or prior ASVCD
        - 40-79 year olds only due to calculator
    - Calculated by http://tools.acc.org/ASCVD-Risk-Estimator-Plus using the Pooled Cohort
    - ASCVD Risk Equations (2013 ACC/AHA Guideline on the Assessment of Cardiovascular Risk. doi: 10.1161/01.cir.0000437741.48606.98).
- Clinic site

Study Sample

Patients attending one of the three participating sites will be screened for eligibility. Planned enrollment is 300 participants (100 per site).

Inclusion criteria (electronic medical record (EMR))

- Age ≥18 years
- Confirmed HIV+ diagnosis (HIV+ ELISA with confirmatory PCR)
- Undetectable HIV viral load: defined as the most recent HIV viral load <200 copies/mL, checked within the past year (assessed via chart abstraction)
- Hypertension: defined as systolic BP >130 mmHg on ≥ 2 occasions in the past 12 months or on an antihypertensive medication (assessed via chart abstraction), and
- Hyperlipidemia: defined as a non HDL cholesterol level >130 mg/dL or on cholesterol lowering medication

***Exclusion criteria** (EMR and patient report)*

- On lipid-lowering medication solely for CVD prevention with evidence of pre-medication non-HDL which was already below National Lipid Association (NLA) target (100 mg/dL)
- On anti-hypertensive medications solely for a non-hypertension indication (e.g. systolic heart failure)
- Severely hearing or speech impaired, or other disability that would limit participation in the intervention components

- In a nursing home and/or receiving in-patient psychiatric care
- Terminal illness with life expectancy < 4 months
- No reliable access to a telephone
- Pregnant, breast-feeding, or planning a pregnancy during the study period
- Planning to move out of the area in the next 12 months
- Non-English Speaking

<u>Outcomes</u>

- ***Primary:*** 12-month change in systolic BP, calculated as the mean of two systolic BP readings measured in the right arm, 1 minute apart.
- ***Secondary:*** 12-month change in non-HDL cholesterol, calculated as total cholesterol – HDL.
- ***Tertiary:*** change in the extended cascade categories for hypertension and hypercholesterolemia
    - *Hypertension: 4-level category*
        - Undiagnosed: hypertension is not listed on the EMR problem list
        - Appropriately diagnosed: hypertension listed on the EMR problem list
        - Appropriately managed: at least one medication prescribed for hypertension
        - At treatment goal: <130 systolic
    - *Hypercholesterolemia: 4-level category*
        - Undiagnosed: hypercholesterolemia is not listed on the EMR problem list
        - Appropriately diagnosed: hypercholesterolemia listed on the EMR problem list
        - Appropriately managed: at least one medication prescribed for hypercholesterolemia
        - At treatment goal, based on lifetime ASCVD risk
            - Low-moderate risk: <130 mg/dl
            - High: <100 mg/dl
            - Very high: <100 mg/dl
    - Measured at each time point
        - Diagnosis, management: EMR review by nurse
        - At goal: measurement at that visit

- ***Exploratory:*** change in additional lipid measurements
    - Total cholesterol
    - Triglycerides
    - HDL
    - LDL

<u>Statistical Analysis</u>

Descriptive analyses

Beginning with the chart review process, we will track ineligibility (number of patients, reasons), declining to participate, and agreeing to participate but not proceeding to the initial visit. Once a patient agrees to the phone screen, we will collect age, race, ethnicity, sex at birth, and gender. We will compare demographics for those who enroll in the study to those who decline participation and to those who are not eligible, using two sample t-tests (or nonparametric equivalent) and χ^2 tests. *(Table S1)*

We will further describe participating patients' characteristics (sociodemographic, lifestyle, medical history). Continuous variables will be reported using mean (SD) or median (Q1, Q3) and categorical

variables will be reported as N (%). These statistics will be reported for the overall sample and by study arm. *(Table 1)*

Primary analyses

The primary intervention effect analyses will utilize continuous, linear mixed models (LMM) to test for differences over time between the study arms. The general mean structure of the LMM we will use to examine the hypotheses is:

$$Y\_ij = \beta\_0 + \beta\_1{*}I(month{=}4) + \beta\_2{*}I(month{=}8) + \beta\_3{*}I(month{=}12) + \beta\_5{*}arm{*}I(month{=}4) + \beta\_6{*}arm{*}I(month{=}8) + \beta\_7{*}arm{*}I(month12) + \beta\_8{*}clinic2 + \beta\_9{*}clinic3$$

where Y_ij represents the outcome of interest (i.e., SBP or non-HDL) for patient i at time j. In this model, we fit a common intercept and arm is the intervention group indicator. Similarly, time is classified, where for example, I(month=12) is a dummy variable equal to 1 for the 12 month time point. Random intercepts will be included for each individual to account for correlation among repeated measurements over time. The primary analytic model will adjust for clinic site as fixed effects, represented with indicator variables clinic2 and clinic3, above. The mixed effects model parameters will be estimated and tested using SAS PROC MIXED (SAS Institute, Cary, NC), and the hypothesis of between-arm differences over time will be tested using estimate statements within PROC MIXED. In particular, β_7, the estimated difference in outcome between arms at 12 months, will be the primary effectiveness outcome assessed.

For the tertiary outcome of cascade category, we will calculate an ordinal four-level variable at baseline, 4, 8, and 12 months. We will use a proportional odds model fit via generalized estimating equations to examine differences over time between study arms. The proportional odds assumption will be assessed using score tests, and the model will be relaxed to partial proportional odds if necessary. Robust sandwich standard errors will be used to account for potential overdispersion and correlation among the repeated observations over time. Similarly to the primary analysis, the model will adjust for clinic site and include the same general mean model specification. The primary hypothesis will be tested via SAS PROC GENMOD to assess whether the estimated proportions in each level at 12 months differ between arms.

All analyses will be conducted following an intention to treat (ITT) principle using all available data. Clinically significant changes specified *a priori* are a 5-point change for SBP and a 15-point change for non-HDL cholesterol.

Missing data

We will assess mechanisms for missing data in this study. LMM, implicitly accommodates missingness when the response is Missing At Random (MAR); that is, when missingness is due either to treatment, to prior outcome, or to other baseline covariates included in the LMM.

Our primary analysis will include all available study-collected data. Those with missing SBP or non-HDL cholesterol will remain missing for the corresponding cascade, even if EMR data is available for the first two categories (because it would be missing for the last categories).

Attrition bias

As part of our examination of missing data, we will assess differences in baseline characteristics (e.g., clinic site, demographics, clinical values, medical history) by retention at each follow up time point. We

will use two sample t-tests (or Wilcoxon rank sum tests) for continuous variables and χ^2 tests for categorical variables.

Subgroup analyses and moderation

Pre-specified sub-group analyses of the primary, secondary, and tertiary outcomes will include clinic site, sex, and baseline ASCVD risk (<20% 10-year risk vs. >20% or prior ASCVD). For each category, we will examine the interactions with intervention arm and time. Generally, the modeling approach will mirror that described above for each outcome. Three separate analyses for each outcome will be conducted to assess the effect of each potential moderator. Models will be fit in SAS PROC MIXED and GENMOD, as described above, and the moderating effect of each of the three factors will be assessed via the hypothesis test of the three-way interactions among subgroup, treatment, and time points (4, 8, and 12 months). As these analyses are exploratory and hypothesis generating in nature, we will not adjust the alpha value. Interpretation will focus on trends and consistent results over time.

Given that some of the participants are treated by study investigator physicians, we will conduct an additional subgroup analysis stratified by treating physician (EXTRA-CVD investigator or not). The primary analysis will be repeated with this stratification variable for our primary, secondary, and tertiary outcomes. Intervention effects will be descriptively compared by treating physician status.

Anticipated tables

Table 1. Patient characteristics (demographic, medical history, lifestyle), overall and by treatment arm

Table 2. Primary, secondary, and tertiary outcomes at each time point, by treatment arm

Table 3. Intervention effects (LMM and proportional odds models) on primary, secondary, and tertiary outcomes

Table 4. Moderation effects: clinic site; sex; baseline ASCVD risk

Table 5. Intervention effects stratified by treating physician (EXTRA-CVD investigator or not)

Table 1. Patient characteristics at baseline, overall and by treatment arm

Variable	Overall	Treatment arm	
		Intervention	Control
N			
Demographic characteristics			
Age (years), Mean (SD)			
Gender			
Male			
Female			
Transgender Male/Transman/FTM			
Unknown			
Ethnicity			
Hispanic/Latinx			
Non-Hispanic/Latinx			
Unknown			
Race			
African American/Black			
White			
Other race			
Multiracial			
Unknown			
Clinical characteristics			
Most recent CD4+ count			
Nadir CD4+ count			
Most recent viral load			
Antihypertensive medications			
None			
Thiazide diuretic			
ACE inhibitor			
Angiotension Receptor Blockers			
Beta Blockers			
Dihydropyridine CCB			
Non-dihydropyridine CCB			
Alpha-2-blocker			
Alpha-blocker			
Hydralazine			
Aldosterone antagonist			
Other			
Number of antihypertensive drugs, median (Q1, Q3)			
Cholesterol lowering drugs			
None			
Statin			
Ezetimibe			
Other			
ASCVD History			
Prior myocardial infarction			
Stroke/transient ischemic attack			
Other ischemic heart disease			
Peripheral artery disease			

Variable	Overall	Treatment arm	
		Intervention	Control
10-year ASCVD Risk score, 40+ years old and without prior history of events			
Low (<5)			
Borderline (5-7.4)			
Intermediate (7.5-19.9)			
High (≥20)			
Other medical history			
Diabetes			
Heart failure			
Current smoker			
Mental health disorder			

Table 2. Primary, secondary, and tertiary outcomes at each time point, by treatment arm

	Baseline		4 months		8 months		12 months	
	Intervention Mean (SD)[a]	Control Mean (SD)[a]	Intervention Mean (SD)[a]	Control Mean (SD)[a]	Intervention Mean (SD)[a]	Control Mean (SD)[a]	Intervention Mean (SD)[a]	Control Mean (SD)[a]
Primary outcome								
Systolic blood pressure, mm Hg								
Secondary outcome								
Non-HDL cholesterol, mg/dL								
Tertiary outcomes								
Hypertension[b], N (%)								
Undiagnosed								
Appropriately diagnosed								
Appropriately managed								
At treatment goal								
Hypercholesterolemia[b], N (%)								
Undiagnosed								
Appropriately diagnosed								
Appropriately managed								
At treatment goal								
Exploratory outcomes								
Total cholesterol, mg/dL								
Triglycerides, mg/dL								
HDL, mg/dL								
LDL, mg/dL								

[a]Unless noted otherwise

[b]Categorized into one of the four mutually exclusive categories: undiagnosed (not listed on the EMR problem list), appropriately diagnosed (listed on the EMR problem list), appropriately managed (at least one prescribed medication for hypertension/hypercholesterolemia), or at treatment goal (<130 systolic blood pressure for hypertension and <130 or <100 mg/dL, depending on risk score, for hypercholesterolemia)

Table 3. Intervention effects (LMM and proportional odds models) on primary, secondary, and tertiary outcomes

	4 months	8 months	12 months
Primary outcome	β (95% CI)	β (95% CI)	β (95% CI)
Systolic blood pressure, mm Hg			
Secondary outcome	β (95% CI)	β (95% CI)	β (95% CI)
Non-HDL cholesterol, mg/dL			
Tertiary outcomes			
Hypertension[b], N (%)			
Undiagnosed			
Appropriately diagnosed			
Appropriately managed			
At treatment goal			
Hypercholesterolemia[b], N (%)			
Undiagnosed			
Appropriately diagnosed			
Appropriately managed			
At treatment goal			
Exploratory outcomes	β (95% CI)	β (95% CI)	β (95% CI)
Total cholesterol, mg/dL			
Triglycerides, mg/dL			
HDL, mg/dL			
LDL, mg/dL			

Table 4. Moderation effects: clinic site; sex; baseline ASCVD risk

	**Systolic Blood Pressure**			**Non-HDL cholesterol**		
	**4 months**	**8 months**	**12 months**	**4 months**	**8 months**	**12 months**
Continuous outcomes	**β (95% CI)**<br>$\mathbf{R^2}$	**β (95% CI)**<br>$\mathbf{R^2}$	**β (95% CI)**<br>$\mathbf{R^2}$	**β (95% CI)**<br>$\mathbf{R^2}$	**β (95% CI)**<br>$\mathbf{R^2}$	**β (95% CI)**<br>$\mathbf{R^2}$
**Clinic**						
Step 1: main effects						
Intervention						
Clinic: Clinic 2						
Clinic: Clinic 3						
Step 2: interaction						
Intervention						
Clinic: Clinic 2						
Clinic: Clinic 3						
Intervention x Clinic 2						
Intervention x Clinic 3						
**Sex**						
Step 1: main effects						
Intervention						
Sex: Female						
Step 2: interaction						
Intervention						
Sex: Female						
Intervention x Female						
**Baseline ASCVD risk**						
Step 1: main effects						
Intervention						
ASCVD risk: High						
Step 2: interaction						
Intervention						
ASCVD risk: High						
Intervention x High risk						
Ordinal outcomes	**Extended cascade category: systolic BP**			**Extended cascade category: non-HDL**		
	**4 month**	**8 month**	**12 month**	**4 month**	**8 month**	**12 month**
	**OR (95% CI)**	**OR (95% CI)**	**OR (95% CI)**	**OR (95% CI)**	**OR (95% CI)**	**OR (95% CI)**

	R^2	R^2	R^2	R^2	R^2	R^2
**Clinic**						
Step 1: main effects						
Intervention						
Diagnosed						
Managed						
At treatment goal						
Clinic: Clinic 2						
Diagnosed						
Managed						
At treatment goal						
Clinic: Clinic 3						
Diagnosed						
Managed						
At treatment goal						
Step 2: interaction						
Intervention						
Diagnosed						
Managed						
At treatment goal						
Clinic: Clinic 2						
Diagnosed						
Managed						
At treatment goal						
Clinic: Clinic 3						
Diagnosed						
Managed						
At treatment goal						
Intervention x Clinic 2						
Diagnosed						
Managed						
At treatment goal						
Intervention x Clinic 3						
Diagnosed						
Managed						
At treatment goal						
**Sex**						

Step 1: main effects						
Intervention						
Diagnosed						
Managed						
At treatment goal						
Sex: Female						
Diagnosed						
Managed						
At treatment goal						
Step 2: interaction						
Intervention						
Diagnosed						
Managed						
At treatment goal						
Sex: Female						
Diagnosed						
Managed						
At treatment goal						
Intervention x Female						
Diagnosed						
Managed						
At treatment goal						
**Baseline ASCVD risk**						
Step 1: main effects						
Intervention						
Diagnosed						
Managed						
At treatment goal						
ASCVD risk: High						
Diagnosed						
Managed						
At treatment goal						
Step 2: interaction						
Intervention						
Diagnosed						
Managed						
At treatment goal						

ASCVD risk: High						
Diagnosed						
Managed						
At treatment goal						
Intervention x High risk						
Diagnosed						
Managed						
At treatment goal						

Interaction terms are intervention*[moderator]*timepoint.

Reference groups: clinic 1 (clinic site), male (sex), low (ASCVD risk), and undiagnosed (extended cascade for HTN and DLD)

ASCVD status: High defined as ≥20% 10-year risk or prior ASCVD; low defined as <20% 10-year risk for ASCVD

Table 5. Intervention effects stratified by treating physician (EXTRA-CVD investigator or not)

	**4 months**	**8 months**	**12 months**
Continuous outcomes	**β (95% CI)**	**β (95% CI)**	**β (95% CI)**
Systolic blood pressure (mmHg) **(primary)**			
EXTRA-CVD investigator physician			
Non-EXTRA-CVD physician			
Non-HDL cholesterol **(secondary)**			
EXTRA-CVD investigator physician			
Non-EXTRA-CVD physician			
Ordinal outcomes	**OR (95% CI)**	**OR (95% CI)**	**OR (95% CI)**
Extended cascade category: systolic BP **(tertiary)**			
EXTRA-CVD investigator physician			
Undiagnosed	Ref.	Ref.	Ref.
Appropriately diagnosed			
Appropriately managed			
At treatment goal			
Non-EXTRA-CVD physician			
Undiagnosed	Ref.	Ref.	Ref.
Appropriately diagnosed			
Appropriately managed			
At treatment goal			
Extended cascade category: non-HDL **(tertiary)**			
EXTRA-CVD investigator physician			
Undiagnosed	Ref.	Ref.	Ref.
Appropriately diagnosed			
Appropriately managed			
At treatment goal			
Non-EXTRA-CVD physician			
Undiagnosed	Ref.	Ref.	Ref.
Appropriately diagnosed			
Appropriately managed			
At treatment goal			

Acronyms

HIV, human immunodeficiency virus (HIV+ indicates diagnosis of HIV)
AIDS, acquired immunodeficiency syndrome
PLHIV, people living with HIV infection
ART, antiretroviral therapy
CVD, cardiovascular disease
ASCVD, atherosclerotic cardiovascular disease
BP, blood pressure
RE-AIM, Reach x Efficacy – Adoption, Implementation, Maintenance
EXTRA-CVD,
RA, research assistance
HDL, high-density lipoprotein ("good" cholesterol)
LDL, low-density lipoproteins ("bad" cholesterol)
Non-HDL, non-HDL cholesterol (calculated as total cholesterol – HDL)
ELISA, Enzyme-Linked Immunosorbent Assay (a technique used to detect antibodies or infectious agents in a sample)
PCR, polymerase chain reaction (a technique to make many copies of a DNA segment)
NLA, National Lipid Association (provides guidelines on best lipids clinical management practices)
EMR, electronic medical record
ITT, intention to treat
MAR, missing at random
FTM, female-to-male (transgender individual)
MTF, male-to-female (transgender individual
GED, general educational development (substitute for high school diploma)
OR, odds ratio
CI, confidence interval
