# Supplementary material for: Nurse-Led Strategy to Improve Blood Pressure and Cholesterol Level Among People With HIV: A Randomized Clinical Trial
Source: JAMA Netw Open. 2024 Mar 5;7(3):e2356445. doi: 10.1001/jamanetworkopen.2023.56445 (PMC10915684; doi:10.1001/jamanetworkopen.2023.56445)
Supplement: Supplement 2. — eFigure 1. Study Accrual eTable 1. Characteristics of People with HIV Who Consented to Participate in EXTRA-CVD Compared to Those Who Declined Participation After an Initial Phone Screen eTable 2. Patient Blood Pressure and Cholesterol Values at Baseline, Overall and by Treatment Arm eFigure 2. Stacked Bar Chart of Blood Pressure Values at Baseline, by Anti-Hypertensive Treatment Status eTable 3. Model-Estimated Outcome Means and Standard Errors or Proportions by Study Arm and Time Point eTable 4. Testing Sex as a Moderator of the Intervention Effects on Outcomes by Including an Interaction Term Between Study Arm, Study Visit, and Sex, With Male Sex Compared to Female (Reference Group) eTable 5. Testing ASCVD Risk as a Moderator of the Intervention Effects on Outcomes by Including an Interaction Term Between Study Arm, Study Visit, and ASCVD Risk, With High ASCVD Risk Compared to Low/Moderate Risk (Reference Group) eTable 6. Testing Site as a Moderator of the Intervention Effects on Outcomes by Including an Interaction Term Between Study Arm, Study Visit, and Site, With Sites 2 and 3 Compared to Site 1 (Reference Group) eTable 7. Intervention Effects Stratified by Treating Physician (EXTRA-CVD Investigator or Not) eTable 8. Adverse Events by Treatment Arm eTable 9. Sensitivity Analysis of Intervention Effects on Primary, Secondary, and Tertiary Outcomes, Adjusted for Baseline Covariates Associated With Attrition [file jamanetwopen-e2356445-s002.pdf]

Supplemental Online Content

Longenecker CT, Jones KA, Hileman CO, et al. Nurse-led strategy to improve blood pressure and cholesterol level among people with HIV: a randomized clinical trial. *JAMA Netw Open*. 2024;7(3):e2356445. doi:10.1001/jamanetworkopen.2023.56445

eFigure 1. Study Accrual

eTable 1. Characteristics of People with HIV Who Consented to Participate in EXTRA-CVD Compared to Those Who Declined Participation After an Initial Phone Screen

eTable 2. Patient Blood Pressure and Cholesterol Values at Baseline, Overall and by Treatment Arm

eFigure 2. Stacked Bar Chart of Blood Pressure Values at Baseline, by Anti-Hypertensive Treatment Status

eTable 3. Model-Estimated Outcome Means and Standard Errors or Proportions by Study Arm and Time Point

eTable 4. Testing Sex as a Moderator of the Intervention Effects on Outcomes by Including an Interaction Term Between Study Arm, Study Visit, and Sex, With Male Sex Compared to Female (Reference Group)

eTable 5. Testing ASCVD Risk as a Moderator of the Intervention Effects on Outcomes by Including an Interaction Term Between Study Arm, Study Visit, and ASCVD Risk, With High ASCVD Risk Compared to Low/Moderate Risk (Reference Group)

eTable 6. Testing Site as a Moderator of the Intervention Effects on Outcomes by Including an Interaction Term Between Study Arm, Study Visit, and Site, With Sites 2 and 3 Compared to Site 1 (Reference Group)

eTable 7. Intervention Effects Stratified by Treating Physician (EXTRA-CVD Investigator or Not)

eTable 8. Adverse Events by Treatment Arm

eTable 9. Sensitivity Analysis of Intervention Effects on Primary, Secondary, and Tertiary Outcomes, Adjusted for Baseline Covariates Associated With Attrition

This supplemental material has been provided by the authors to give readers additional information about their work.

© 2024 Longenecker CT et al. *JAMA Network Open*

eFigure 1. Study Accrual

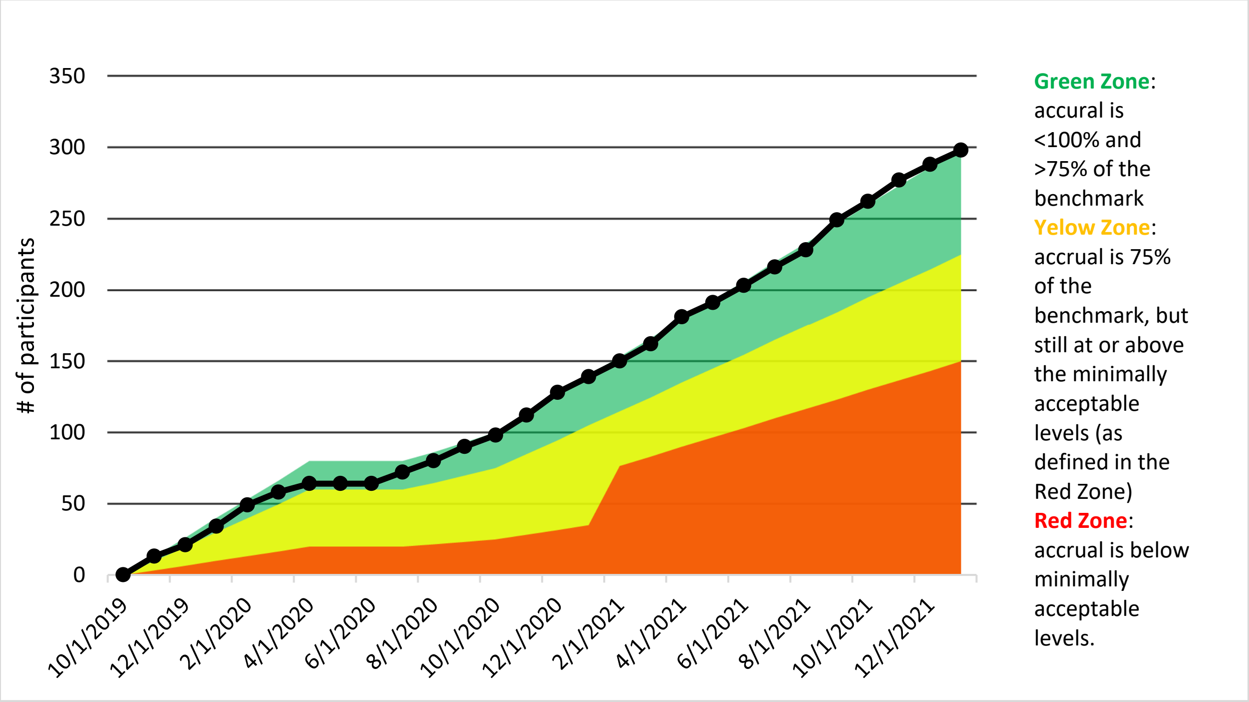

© 2024 Longenecker CT et al. *JAMA Network Open*

eTable 1. Characteristics of People with HIV Who Consented to Participate in EXTRA-CVD Compared to Those Who Declined Participation After an Initial Phone Screen

**Variable**	**Consented to participate in EXTRA-CVD**	**Did not consent to participate in EXTRA-CVD**	**p-value**
N	299[b]	106	
Demographics			
Age (years), Mean (SD)	57.3 (9.6)	53.4 (11.4)	.003
Age (years), Median (Q1, Q3)	59.0 (52.0, 64.0)	56.0 (45.0, 62.0)	.003
Gender			.29
Male	234 (78.3%)	80 (75.5%)	
Female	62 (20.7%)	23 (21.7%)	
Transgender Male/Transman/FTM	1 (0.3%)	0 (0.0%)	
Transgender Female/Transwoman/MTF	2 (0.7%)	1 (0.9%)	
Other gender	0 (0.0%)	1 (0.9%)	
Unreported	0 (0.0%)	1 (0.9%)	
Sex			.85
Female	62 (20.7%)	23 (21.7%)	
Male	232 (77.6%)	82 (77.4%)	
Unreported	5 (1.7%)	1 (0.9%)	
Hispanic or Latino ethnicity			.003
Non-Hispanic	281 (94.0%)	101 (95.3%)	
Hispanic/Latino	17 (5.7%)	1 (0.9%)	
Unreported	1 (0.3%)	4 (3.8%)	
Race			.04
African American/Black	181 (60.5%)	65 (61.3%)	
American Indian/Alaskan Native	0 (0.0%)	1 (0.9%)	
White	100 (33.4%)	35 (33.0%)	
Other race	9 (3.0%)	1 (0.9%)	
Multiracial	7 (2.3%)	0 (0.0%)	
Unknown	2 (0.7%)	4 (3.8%)	

[a] This table includes all patients who were deemed eligible for EXTRA-CVD following the medical record review and participant screening. Demographic information was collected at the participant screening, as approved by the IRB.

[b] As shown in Figure 1 of the manuscript, n=299 is two higher than the n=297 who were included in the final analysis. One participant withdrew after consent but prior to randomization; another participant withdrew after randomization but prior to the baseline visit.

© 2024 Longenecker CT et al. *JAMA Network Open*

eTable 2. Patient Blood Pressure and Cholesterol Values at Baseline, Overall and by Treatment Arm

Variable	Overall N=297	Intervention N=149	Control N=148
Blood Pressure			
Systolic blood pressure, Mean (SD)	135.0 (18.8)	135.9 (18.1)	134.0 (19.5)
Diastolic blood pressure, Mean (SD)	81.1 (12.1)	81.2 (12.2)	81.0 (12.0)
Cholesterol			
Total cholesterol, Mean (SD)	184.7 (46.1)	185.1 (45.3)	184.4 (46.9)
LDL, Mean (SD)	111.8 (39.6)	111.5 (38.6)	112.1 (40.6)
HDL, Mean (SD)	45.2 (14.5)	45.8 (14.7)	44.7 (14.3)
Non-HDL, Mean (SD)	139.9 (44.6)	139.5 (44.6)	140.3 (44.9)
Triglycerides, Median (Q1, Q3)[a]	121.0 (89.0, 184.0)	127.0 (95.0, 182.0)	112.0 (85.0, 191.0)

[a]Median (Q1, Q3) presented due to skewness of the data

© 2024 Longenecker CT et al. *JAMA Network Open*

eFigure 2. Stacked Bar Chart of Blood Pressure Values at Baseline, by Anti-Hypertensive Treatment Status

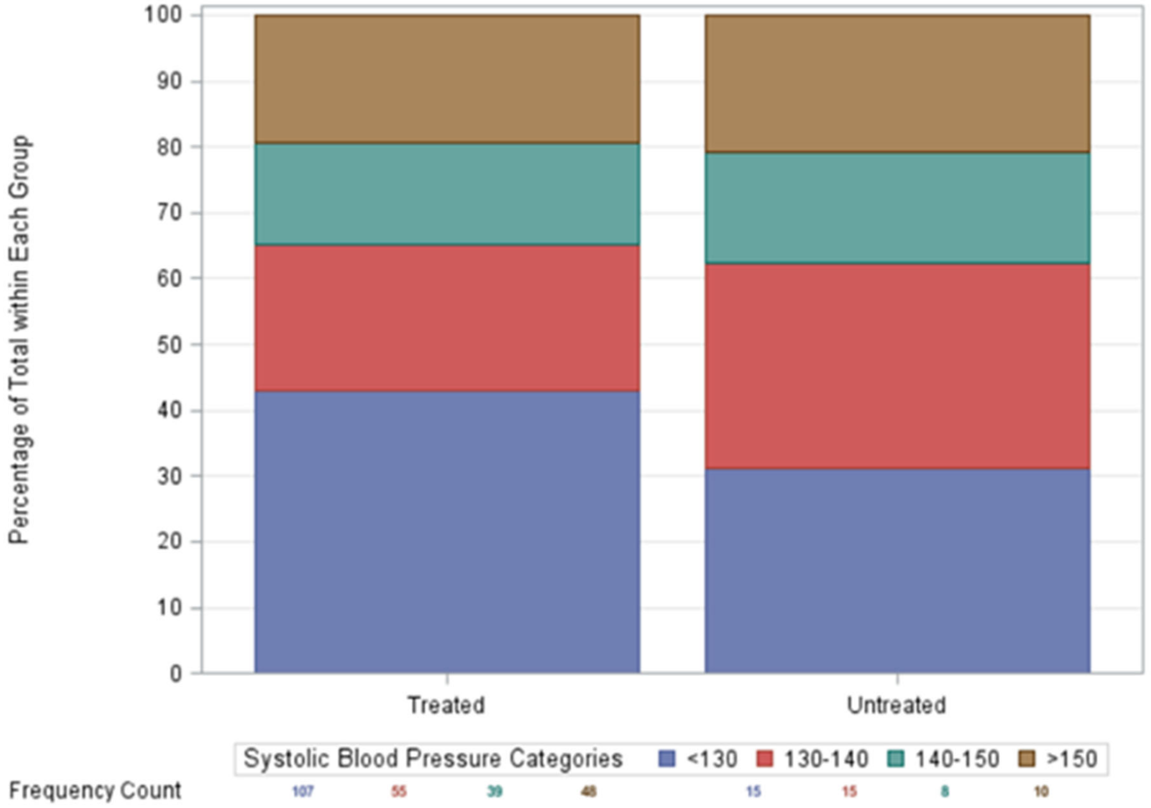

eTable 3. Model-Estimated Outcome Means and Standard Errors or Proportions by Study Arm and Time Point

Outcomes	Baseline		4 months		8 months		12 months	
	Intervention estimate Mean (SE)[a]	Control estimate Mean (SE)[a]	Intervention estimate Mean (SE)[a]	Control estimate Mean (SE)[a]	Intervention estimate Mean (SE)[a]	Control estimate Mean (SE)[a]	Intervention estimate Mean (SE)[a]	Control estimate Mean (SE)[a]
**Primary outcome**								
Systolic blood pressure, mm Hg	134.9 (1.0)	134.9 (1.0)	129.9 (1.5)	136.3 (1.5)	129.3 (1.6)	133.0 (1.4)	129.7 (1.5)	133.9 (1.4)
**Secondary outcome**								
Non-HDL cholesterol, mg/dL	139.9 (2.5)	139.9 (2.5)	127.4 (3.5)	135.7 (3.4)	120.9 (3.5)	131.9 (3.3)	114.7 (3.5)	132.1 (3.3)
**Tertiary outcomes**								
Hypertension treatment cascade[b]	**%**	**%**	**%**	**%**	**%**	**%**	**%**	**%**
Untreated	10.8%	10.8%	7.8%	9.9%	3.2%	7.7%	5.7%	11.6%
Treated	47.3%	47.3%	34.3%	45.7%	33.2%	45.3%	34.4%	48.6%
At treatment goal	42.0%	42.0%	57.9%	44.4%	63.6%	47.0%	59.9%	39.8%
Hypercholesterolemia treatment cascade[b]	**%**	**%**	**%**	**%**	**%**	**%**	**%**	**%**
Untreated	37.5%	37.5%	18.1%	27.5%	5.9%	16.3%	5.3%	27.1%
Treated	20.5%	20.5%	15.7%	18.0%	9.0%	16.3%	3.6%	14.1%
At treatment goal	42.0%	42.0%	66.3%	54.5%	85.1%	67.4%	91.1%	58.7%
**Exploratory outcomes**								
Total cholesterol, mg/dL	184.9 (2.6)	184.9 (2.6)	172.4 (3.6)	179.9 (3.5)	165.8 (3.6)	176.4 (3.4)	161.5 (3.6)	177.5 (3.4)
HDL cholesterol, mg/dL	45.4 (0.9)	45.4 (0.9)	45.2 (1.1)	44.3 (1.0)	45.1 (1.1)	43.5 (1.0)	46.4 (1.1)	45.6 (1.0)
LDL cholesterol, mg/dL	111.5 (3.7)	111.5 (3.7)	107.4 (5.9)	115.4 (5.7)	93.3 (6.0)	104.7 (5.5)	96.4 (5.9)	107.0 (5.6)
Triglycerides, mg/dL	165.7 (7.7)	165.7 (7.7)	152.6 (10.4)	163.7 (10.1)	136.6 (10.5)	169.2 (9.8)	133.6 (10.5)	161.6 (9.9)

[a]Unless noted otherwise

© 2024 Longenecker CT et al. *JAMA Network Open*

[b]Categorized into one of the three mutually exclusive categories: untreated (regardless of whether it has been diagnosed), appropriately managed (at least one prescribed medication for hypertension/hypercholesterolemia), or at treatment goal (<130 systolic blood pressure for hypertension and <130 or <100 mg/dL, depending on risk score, for hypercholesterolemia)

© 2024 Longenecker CT et al. *JAMA Network Open*

eTable 4. Testing Sex as a Moderator of the Intervention Effects on Outcomes by Including an Interaction Term Between Study Arm, Study Visit, and Sex, With Male Sex Compared to Female (Reference Group)

	Intervention effect						
**Outcomes**	**Overall joint test**	**4 months**		**8 months**		**12 months**	
	**p**	**Beta (95% CI)**	**p**	**Beta (95% CI)**	**p**	**Beta (95% CI)**	**p**
**Primary outcome**							
Systolic blood pressure, mm Hg	.06	11.8 (2.0, 21.6)	.02	9.6 (-0.1, 19.3)	.05	5.9 (-3.8, 15.5)	.23
**Secondary outcome**							
Non-HDL cholesterol, mg/dL	.70	-11.1 (-31.6, 9.4)	.29	0.1 (-20.1, 20.4)	.99	0.4 (-19.9, 20.7)	.97
**Tertiary outcomes**							
Hypertension treatment cascade	.81						
Treated		1.4 (0.1, 16.1)	.77	0.5 (0.0, 9.0)	.61	0.5 (0.0, 6.5)	.60
At treatment goal		0.4 (0.0, 4.8)	.49	0.3 (0.0, 4.6)	.35	0.8 (0.1, 9.2)	.85
Hypercholesterolemia treatment cascade	.80						
Treated		1.9 (0.1, 34.7)	.68	0.6 (0.0, 14.3)	.73	0.1 (0.0, 3.0)	.20
At treatment goal		4.2 (0.3, 61.5)	.30	1.2 (0.1, 18.2)	.90	0.5 (0.0, 6.7)	.56
**Exploratory outcomes**							
Total cholesterol, mg/dL	.58	-12.7 (-33.5, 8.2)	.23	2.5 (-18.1, 23.1)	.81	-4.8 (-25.4, 15.9)	.65
HDL cholesterol, mg/dL	.36	-2.5 (-8.1, 3.0)	.37	0.9 (-4.6, 6.4)	.75	-3.8 (-9.3, 1.7)	.18
LDL cholesterol, mg/dL	.93	-7.5 (-46.7, 31.7)	.71	5.6 (-33.2, 44.3)	.78	8.0 (-30.9, 46.9)	.69

© 2024 Longenecker CT et al. *JAMA Network Open*

Outcomes	Intervention effect						
	Overall joint test	4 months		8 months		12 months	
	p	Beta (95% CI)	p	Beta (95% CI)	p	Beta (95% CI)	p
Triglycerides, mg/dL	.40	2.0 (-57.6, 61.5)	.95	-47.1 -106.0, 11.9)	.12	-7.7 (-66.7, 51.3)	.80

Overall joint test tests whether there is an overall moderating effect by sex

© 2024 Longenecker CT et al. *JAMA Network Open*

eTable 5. Testing ASCVD Risk as a Moderator of the Intervention Effects on Outcomes by Including an Interaction Term Between Study Arm, Study Visit, and ASCVD Risk, With High ASCVD Risk Compared to Low/Moderate Risk (Reference Group)

	**Intervention effect**						
**Outcomes**	**Overall joint test**	**4 months**		**8 months**		**12 months**	
	**p**	**Beta (95% CI)**	**p**	**Beta (95% CI)**	**p**	**Beta (95% CI)**	**p**
**Primary outcome**							
Systolic blood pressure, mm Hg	.24	-6.3 (-14.9, 2.3)	.15	-7.9 (-16.3, 0.6)	.07	-3.5 (-12.0, 5.0)	.42
**Secondary outcome**							
Non-HDL cholesterol, mg/dL	.53	5.1 (-12.5, 22.6)	.57	13.0 (-4.3, 30.4)	.14	5.5 (-12.0, 23.1)	.54
**Tertiary outcomes**							
Hypertension treatment cascade	.31						
Treated		1.8 (0.1, 24.0)	.65	0.8 (0.0, 17.5)	.89	0.3 (0.0, 3.6)	.33
At treatment goal		4.2 (0.3, 51.2)	.26	2.2 (0.1, 44.0)	.61	2.5 (0.2, 29.2)	.47
Hypercholesterolemia treatment cascade	.30						
Treated		11.4 (0.9, 147.7)	.06	6.2 (0.4, 99.2)	.20	3.1 (0.2, 62.3)	.45
At treatment goal		1.7 (0.2, 17.7)	.65	2.9 (0.2, 36.0)	.40	6.4 (0.5, 91.1)	.17
**Exploratory outcomes**							
Total cholesterol, mg/dL	.66	2.8 (-15.1, 20.7)	.76	11.3 (-6.5, 29.0)	.21	4.4 (-13.5, 22.3)	.63
HDL cholesterol, mg/dL	.46	-1.7 (-6.5, 3.2)	.50	-2.6 (-7.4, 2.2)	.30	-3.8 (-8.6, 1.0)	.12
LDL cholesterol, mg/dL	.12	38.9 (4.5, 73.4)	.03	12.9 (-21.0, 46.8)	.46	-9.6 (-44.1, 25.0)	.59

© 2024 Longenecker CT et al. *JAMA Network Open*

Outcomes	Intervention effect						
	Overall joint test	4 months		8 months		12 months	
	p	Beta (95% CI)	p	Beta (95% CI)	p	Beta (95% CI)	p
Triglycerides, mg/dL	.63	13.0 (-34.3, 60.3)	.59	13.1 (-33.8, 59.9)	.58	31.7 (-15.5, 79.0)	.19

Overall joint test tests whether there is an overall moderating effect by ASCVD risk

© 2024 Longenecker CT et al. *JAMA Network Open*

eTable 6. Testing Site as a Moderator of the Intervention Effects on Outcomes by Including an Interaction Term Between Study Arm, Study Visit, and Site, With Sites 2 and 3 Compared to Site 1 (Reference Group)

	Intervention effect						
**Outcomes**	**Overall joint test**	**4 months**		**8 months**		**12 months**	
	**p**	**Beta (95% CI)**	**p**	**Beta (95% CI)**	**p**	**Beta (95% CI)**	**p**
**Primary outcome**							
Systolic blood pressure, mm Hg	.70		.57		.80		.42
SBP, Site B		2.8 (-6.7, 12.3)	.57	-2.6 (-12.1, 7.0)	.59	-4.6 (-14.1, 4.9)	.34
SBP, Site C		-2.7 (-12.6, 7.3)	.60	0.5 (-9.3, 10.2)	.92	-6.3 (-16.2, 3.6)	.21
**Secondary outcome**							
Non-HDL cholesterol, mg/dL	.82		.93		.93		.39
Non-HDL, Site B		1.0 (-18.8, 20.8)	.92	-3.0 (-23.0, 16.9)	.77	13.9 (-6.0, 33.8)	.17
Non-HDL, Site C		3.9 (-17.0, 24.8)	.72	-3.5 (-23.9, 17.0)	.74	7.4 (-13.3, 28.1)	.48
**Tertiary outcomes**							
Hypertension treatment cascade	.91		.84		.53		.74
Treated, Site B		Not est.	.94	0.8 (0.0, 30.5)	.90	Not est.	.98
Treated, Site C		Not est.	.94	0.5 (0.0, 12.5)	.64	Not est.	.98
At treatment goal, Site B		Not est.	.93	3.9 (0.1, 138.9)	.45	Not est.	.99
At treatment goal, Site C		Not est.	.94	1.3 (0.1, 32.5)	.86	Not est.	.98

© 2024 Longenecker CT et al. *JAMA Network Open*

Outcomes	Intervention effect						
	Overall joint test	4 months		8 months		12 months	
	p	Beta (95% CI)	p	Beta (95% CI)	p	Beta (95% CI)	p
Hypercholesterolemia treatment cascade	.12		.95		.91		.01
Treated, Site B		0.9 (0.0, 21.4)	.92	1.3 (0.0, 43.0)	.88	61.3 (1.5, 2423.9)	.03
Treated, Site C		2.8 (0.1, 69.3)	.54	0.3 (0.0, 11.5)	.52	0.6 (0.0, 23.1)	.76
At treatment goal, Site B		1.3 (0.1, 22.7)	.86	1.7 (0.1, 41.1)	.75	0.5 (0.0, 11.0)	.64
At treatment goal, Site C		2.1 (0.1, 39.5)	.62	0.6 (0.0, 14.6)	.77	0.2 (0.0, 4.3)	.33
**Exploratory outcomes**							
Total cholesterol, mg/dL	.98		.98		.93		.74
Total cholesterol, Site B		-1.5 (-21.7, 18.7)	.88	-3.5 (-23.9, 16.8)	.73	7.3 (-13.0, 27.6)	.48
Total cholesterol, Site C		0.2 (-21.0, 21.5)	.98	-3.1 (-23.9, 17.7)	.77	6.6 (-14.4, 27.7)	.54
HDL cholesterol, mg/dL	**.02**		.41		.84		.009
HDL, Site B		-2.9 (-8.2, 2.5)	.29	1.5 (-3.9, 6.9)	.59	-8.0 (-13.3, -2.6)	.004
HDL, Site C		-3.5 (-9.1, 2.2)	.23	1.3 (-4.2, 6.8)	.64	-1.3 (-6.9, 4.3)	.64
LDL cholesterol, mg/dL	.67		.66		.90		.17
LDL, Site B		-1.6 (-38.9, 35.6)	.93	-2.6 (-40.4, 35.1)	.89	5.5 (-32.1, 43.0)	.78
LDL, Site C		16.1 (-24.3, 56.4)	.44	6.5 (-32.6, 45.6)	.74	36.1 (-3.8, 76.0)	.08
Triglycerides, mg/dL	.41		.29		.47		.89
Triglycerides, Site B		-7.1 (-64.7, 50.6)	.81	-32.2 (-90.5, 26.0)	.28	14.1 (-43.9, 72.1)	.63

© 2024 Longenecker CT et al. *JAMA Network Open*

Outcomes	Intervention effect						
	Overall joint test	4 months		8 months		12 months	
	p	Beta (95% CI)	p	Beta (95% CI)	p	Beta (95% CI)	p
Triglycerides, Site C		39.8 (-21.0, 100.7)	.20	-31.0 (-90.6, 28.5)	.31	8.1 (-52.1, 68.4)	.79

Overall joint test tests whether there is an overall moderating effect by site. Not est. = not estimable, valid estimates not available in some of the multinomial regression models

© 2024 Longenecker CT et al. *JAMA Network Open*

eTable 7. Intervention Effects Stratified by Treating Physician (EXTRA-CVD Investigator or Not)

**Outcomes**	**Intervention effects**											
	**4 months**				**8 months**				**12 months**			
	**Study clinician**		**None**		**Study clinician**		**None**		**Study clinician**		**None**	
	**Beta (95% CI)**	***p***	**Beta (95% CI)**	***p***	**Beta (95% CI)**	***p***	**Beta (95% CI)**	***p***	**Beta (95% CI)**	***p***	**Beta (95% CI)**	***p***
**Primary outcome**												
Systolic blood pressure, mm Hg	-7.3 (-15.8, 1.2)	.09	**-5.6 (-10.1, -1.2)**	**.01**	**-10.3 (-18.6, -2.0)**	**.01**	-1.0 (-5.4, 3.5)	.67	3.6 (-4.7, 11.8)	.39	**-6.3 (-10.7, -1.9)**	**.006**
**Secondary outcome**												
Non-HDL cholesterol, mg/dL	-14.9 (-33.5, 3.6)	.11	-5.7 (-15.0, 3.6)	.23	-12.2 (-30.4, 5.9)	.19	**-10.3 (-19.5, -1.0)**	**.03**	-14.6 (-32.7, 3.5)	.11	**-17.6 (-26.9, -8.4)**	**< .001**
**Tertiary outcomes**												
Hypertension treatment cascade	OR (95% CI)	*p*	OR (95% CI)	*p*	OR (95% CI)	*p*	OR (95% CI)	*p*	OR (95% CI)	*p*	OR (95% CI)	*p*
Treated	Not est.	**< .001**	0.5 (0.1, 1.7)	.27	Not est.	.82	1.4 (0.4, 5.8)	.61	Not est.	< .001	1.1 (0.3, 3.6)	.90
At treatment goal	Not est.	**< .001**	1.2 (0.4, 3.7)	.76	Not est.	.81	2.0 (0.5, 7.5)	.31	Not est.	< .001	2.5 (0.8, 8.0)	.12
Hypercholesterolemia treatment cascade	OR (95% CI)	*p*	OR (95% CI)	*p*	OR (95% CI)	*p*	OR (95% CI)	*p*	OR (95% CI)	*p*	OR (95% CI)	*p*
Treated	0.7 (0.1, 9.6)	.79	1.5 (0.4, 6.6)	.57	1.9 (0.1, 27.1)	.65	1.4 (0.3, 7.1)	.67	1.3 (0.1, 21.1)	.85	1.1 (0.2, 5.9)	.91
At treatment goal	1.4 (0.1, 19.4)	.79	1.7 (0.5, 5.5)	.41	15.2 (1.0, 233.3)	.05	2.0 (0.5, 7.5)	.31	7.7 (0.5, 116.6)	.14	**6.9 (1.9, 25.7)**	**.004**
**Exploratory outcomes**												

© 2024 Longenecker CT et al. *JAMA Network Open*

Outcomes	Intervention effects											
	4 months				8 months				12 months			
	Study clinician		None		Study clinician		None		Study clinician		None	
	**Beta (95% CI)**	***p***	**Beta (95% CI)**	***p***	**Beta (95% CI)**	***p***	**Beta (95% CI)**	***p***	**Beta (95% CI)**	***p***	**Beta (95% CI)**	***p***
Total cholesterol, mg/dL	-10.3 (-29.7, 9.1)	.30	-6.2 (-15.6, 3.1)	.19	-9.5 (-28.5, 9.5)	.32	**-10.4 (-19.7, -1.1)**	**.03**	-11.4 (-30.4, 7.5)	.24	**-16.7 (-26.1, -7.4)**	**< .001**
HDL cholesterol, mg/dL	3.3 (-0.9, 7.5)	.12	0.1 (-2.6, 2.8)	.93	1.5 (-2.6, 5.6)	.47	1.9 (-0.8, 4.5)	.17	2.3 (-1.8, 6.5)	.26	0.4 (-2.3, 3.1)	.77
LDL cholesterol, mg/dL	-12.4 (-28.6, 3.8)	.13	-1.1 (-21.5, 19.3)	.91	-7.0 (-23.1, 9.0)	.39	-7.1 (-27.3, 13.0)	.49	-9.8 (-25.7, 6.1)	.23	-4.3 (-24.7, 16.1)	.68
Triglycerides, mg/dL	-15.3 (-71.0, 40.4)	.59	-9.9 (-36.7, 17.0)	.47	-49.0 -103.7, 5.7)	.08	**-28.8 (-55.4, -2.1)**	**.03**	-22.1 (-76.8, 32.6)	.43	**-30.5 (-57.3, -3.7)**	**.03**

Not est. = not estimable, valid estimates not available in some of the multinomial regression models

© 2024 Longenecker CT et al. *JAMA Network Open*

eTable 8. Adverse Events by Treatment Arm

Variable	EXTRA-CVD Intervention	Education Control
N, total number of participants	149	148
Total participants with ≥1 AE	60 (40.3%)	55 (37.2%)
Number of AE per participant, among all participants		
Mean (SD)	0.7 (1.2)	0.8 (1.6)
Median (Q1, Q3)	0 (0, 1)	0 (0, 1)
Number of AE per participant, among those with ≥1 AE		
Mean (SD)	1.8 (1.2)	2.3 (1.9)
Median (Q1, Q3)	1 (1, 2)	2 (1, 3)
N, total number of adverse events	107	125
Serious AE	47 (43.9%)	47 (37.6%)
AE unexpected	39 (36.4%)	44 (35.2%)
AE grade		
Mild (Grade 1)	11 (10.3%)	12 (9.6%)
Moderate (Grade 2)	30 (28.0%)	44 (35.2%)
Severe (Grade 3)	56 (52.3%)	53 (42.4%)
Life-threatening (Grade 4)	8 (7.5%)	14 (11.2%)
Death (Grade 5)	2 (1.9%)	2 (1.6%)
AE related to study		
Not related	81 (75.7%)	96 (76.8%)
Unlikely to be related	22 (20.6%)	26 (20.8%)
Possibly related	1 (0.9%)	3 (2.4%)
Probably related	2 (1.9%)	0 (0.0%)
Definitely related	1 (0.9%)	0 (0.0%)

AE, adverse event

© 2024 Longenecker CT et al. *JAMA Network Open*

eTable 9. Sensitivity Analysis of Intervention Effects on Primary, Secondary, and Tertiary Outcomes, Adjusted for Baseline Covariates Associated With Attrition[a]

	Intervention effect					
**Outcomes**	**4 months**		**8 months**		**12 months**	
**Primary outcome**	Beta (95% CI)	*p*	Beta (95% CI)	*p*	Beta (95% CI)	*p*
Systolic blood pressure, mm Hg	**-5.7 (-9.7, -1.7)**	**.005**	-3.1 (-7.0, 0.9)	.13	-3.6 (-7.5, 0.4)	.08
**Secondary outcome**	Beta (95% CI)	*p*	Beta (95% CI)	*p*	Beta (95% CI)	*p*
Non-HDL cholesterol, mg/dL	**-8.7 (-17.1, -0.4)**	**.04**	**-11.2 (-19.5, -2.9)**	**.008**	**-17.6 (-25.9, -9.4)**	**< .001**
**Tertiary outcomes**	OR (95% CI)	*p*	OR (95% CI)	*p*	OR (95% CI)	*p*
Hypertension treatment cascade						
Treated	0.9 (0.3, 2.6)	.80	1.5 (0.4, 5.6)	.50	1.2 (0.4, 3.8)	.70
At treatment goal	1.3 (0.5, 3.7)	.61	2.6 (0.7, 8.9)	.14	2.4 (0.8, 6.9)	.12
Hypercholesterolemia treatment cascade						
Treated	1.4 (0.4, 4.8)	.55	1.6 (0.4, 5.8)	.51	1.3 (0.3, 5.2)	.68
At treatment goal	2.0 (0.7, 6.0)	.20	**3.8 (1.2, 12.2)**	**.03**	**8.8 (2.7, 28.2)**	**< .001**
**Exploratory outcomes**	Beta (95% CI)	*p*	Beta (95% CI)	*p*	Beta (95% CI)	*p*
Total cholesterol, mg/dL	-6.6 (-14.8, 1.5)	.11	**-9.4 (-17.5, -1.3)**	**.02**	**-15.2 (-23.4, -7.1)**	**< .001**
HDL cholesterol, mg/dL	1.1 (-1.2, 3.3)	.36	1.8 (-0.4, 4.1)	.11	1.0 (-1.2, 3.3)	.37
LDL cholesterol, mg/dL	-8.0 (-23.9, 7.8)	.32	-11.4 (-27.0, 4.1)	.15	-11.0 (-26.7, 4.7)	.17
Triglycerides, mg/dL	-12.4 (-36.5, 11.7)	.31	**-34.3 (-58.1, -10.5)**	**.005**	**-29.6 (-53.5, -5.7)**	**.02**

[a] Covariates associated with attrition were race, ethnicity, smoking status, mental health history, insurance status, and employment status. Sex was added as an additional covariate given the results of the moderation analyses. Covariates were tested for multicollinearity before entering into the final model

© 2024 Longenecker CT et al. *JAMA Network Open*
