# Supplementary material for: Nurse-Led Strategy to Improve Blood Pressure and Cholesterol Level Among People With HIV: A Randomized Clinical Trial
Source: JAMA Netw Open. 2024 Mar 5;7(3):e2356445. doi: 10.1001/jamanetworkopen.2023.56445 (PMC10915684; doi:10.1001/jamanetworkopen.2023.56445)
Supplement: Supplement 3. — Data Sharing Statement [file jamanetwopen-e2356445-s003.pdf]

# Data Sharing Statement

Longenecker. Nurse-Led Strategy to Improve Blood Pressure and Cholesterol Level Among People With HIV. *JAMA Netw Open*. Published March 05, 2024.
doi:10.1001/jamanetworkopen.2023.56445

**Data**
**Data available:** Yes
**Data types:** Deidentified participant data
**How to access data:** ctlongen@uw.edu
**When available:** beginning date: 06-01-2025

**Supporting Documents**
**Document types:** None

**Additional Information**
**Who can access the data:** researchers whose proposed use of the data has been approved by the EXTRA-CVD publications committee
**Types of analyses:** for analyses approved by the EXTRA-CVD publications committee
**Mechanisms of data availability:** after approval of a proposal
